# The state of Medusozoa genomics: current evidence and future challenges

**DOI:** 10.1093/gigascience/giac036

**Published:** 2022-05-17

**Authors:** Mylena D Santander, Maximiliano M Maronna, Joseph F Ryan, Sónia C S Andrade

**Affiliations:** Departamento de Genética e Biologia Evolutiva, Instituto de Biociências, Universidade São Paulo, 277 Rua do Matão, Cidade Universitária, São Paulo 05508-090, Brazil; Departamento de Zoologia, Instituto de Biociências, Universidade de São Paulo, São Paulo, 101 Rua do Matão, Cidade Universitária, São Paulo 05508-090, Brazil; Whitney Laboratory for Marine Bioscience, University of Florida, 9505 Ocean Shore Blvd, St. Augustine, FL 32080, USA; Department of Biology, University of Florida, 220 Bartram Hall, Gainesville, FL 32611, USA; Departamento de Genética e Biologia Evolutiva, Instituto de Biociências, Universidade São Paulo, 277 Rua do Matão, Cidade Universitária, São Paulo 05508-090, Brazil

**Keywords:** annotation, completeness, assembly, genome size, chromosome number, collaborative genomics

## Abstract

Medusozoa is a widely distributed ancient lineage that harbors one-third of Cnidaria diversity divided into 4 classes. This clade is characterized by the succession of stages and modes of reproduction during metagenic lifecycles, and includes some of the most plastic body plans and life cycles among animals. The characterization of traditional genomic features, such as chromosome numbers and genome sizes, was rather overlooked in Medusozoa and many evolutionary questions still remain unanswered. Modern genomic DNA sequencing in this group started in 2010 with the publication of the *Hydra vulgaris* genome and has experienced an exponential increase in the past 3 years. Therefore, an update of the state of Medusozoa genomics is warranted. We reviewed different sources of evidence, including cytogenetic records and high-throughput sequencing projects. We focused on 4 main topics that would be relevant for the broad Cnidaria research community: (i) taxonomic coverage of genomic information; (ii) continuity, quality, and completeness of high-throughput sequencing datasets; (iii) overview of the Medusozoa specific research questions approached with genomics; and (iv) the accessibility of data and metadata. We highlight a lack of standardization in genomic projects and their reports, and reinforce a series of recommendations to enhance future collaborative research.

## Background

**Figure 1:**
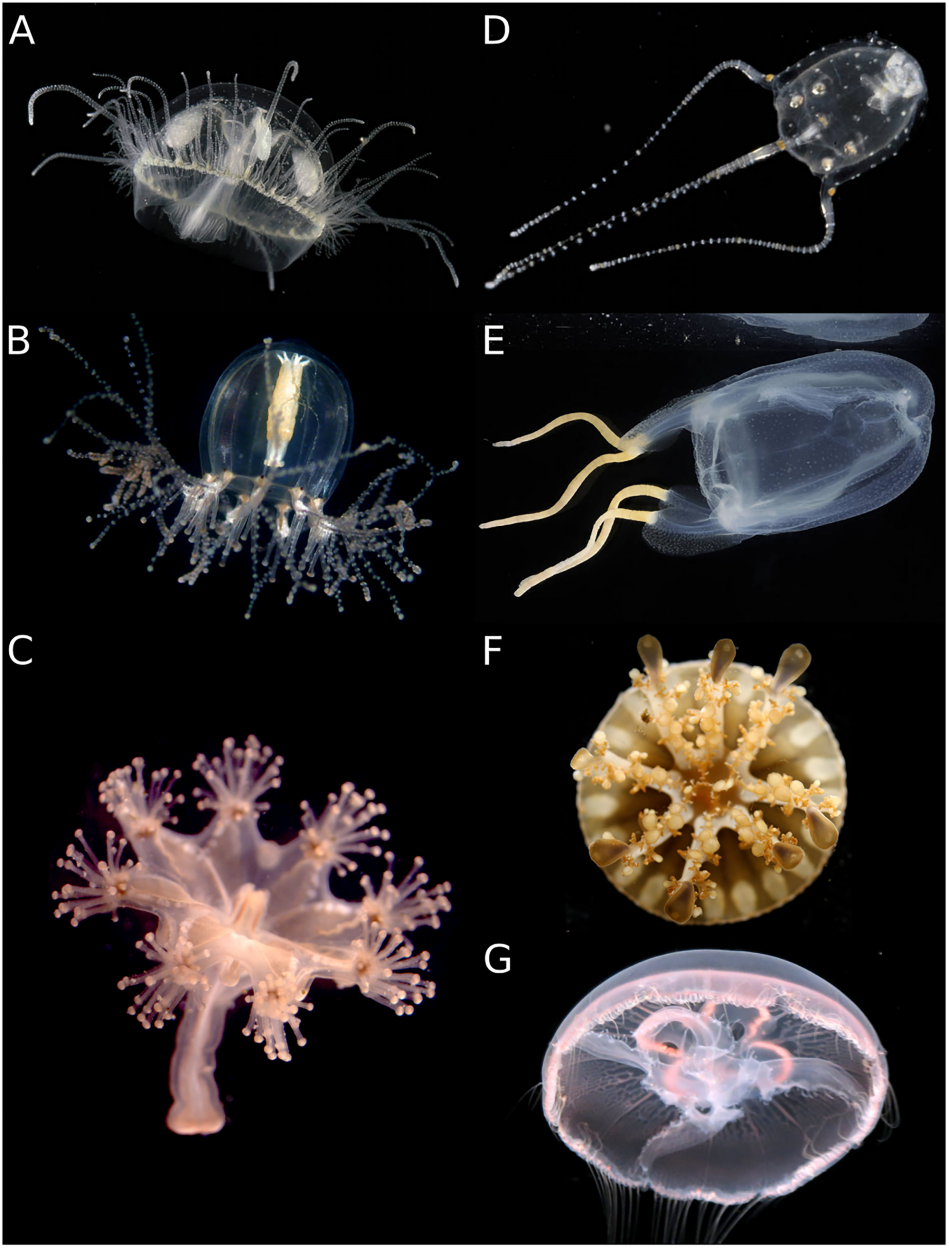
Medusozoa diversity. Examples of different genera covered by this review belong to Hydrozoa (A, B), Staurozoa (C), Cubozoa (D, E), and Scyphozoa (FG). (A) *Craspedacusta sowerbii*, (B) *Cladonema radiatum*, (C) *Haliclystus sanjuanensis*, (D) *Copula sivickisi*, (E) *Tamoya haplonema*, (F) *Cassiopea xamachana*, (G) *Aurelia aurita*. Credits to Alvaro E. Migotto (A, B, E, D), Marta Chiodin (C) and Joseph F. Ryan (F, G). Photographs A, B, D, E were obtained from Cifonauta [27]. Photographs are not to scale.

Medusozoa subphylum includes nearly 4,055 species of invertebrates distributed in the classes Hydrozoa, Cubozoa, Staurozoa, and Scyphozoa [1], which are found at all latitudes in almost all aquatic environments, from freshwater to marine, and from shallow to deep waters (Fig. 1). Medusozoa species, together with the other cnidarian classes (i.e., Anthozoa and Endocnidozoa), harbor some of the most plastic life cycles and diverse body plans among animals [2] and represent one of its early diverging groups, with all major cnidarian lineages already present 500 million years ago [3].

The Medusozoa clade is characterized by different evolutionary novelties, such as the presence of linear mitochondria and the adult pelagic stage, also known as medusa or jellyfish [4–6]. Most medusozoan life cycles are characterized by the succession of different stages, including a larval, benthic asexually reproducing polyp stage and a sexually reproducing jellyfish stage [6,7]. This ancestral metagenic life cycle pattern is highly plastic and in some groups has been extensively modified or even lost. For example, several lineages have lost the pelagic medusae or reduced it to a reproductive structure, or acquired colonial lifestyles during the benthic phase [8–10]. Other novel traits have emerged in Medusozoa such as complex body patterns, neuromuscular systems, and sensory organs [11].

The history of Medusozoa genomics started with pioneer cytogenetics reports (e.g., [12,13]) and was continued later by genome size estimations [14,15]. Over the past 20 years, technological advances and cost reduction of genome-scale sequencing platforms have led to a steady increase in both the number and diversity of sequenced genomes and transcriptomes [16,17]. Medusozoa is not an exception, as numerous genomic resources have become available for model and non-model species, especially in the past 3 years. This advance has enabled the study of the genetic basis of many Medusozoa novel traits (e.g., [18–22]. Previous reviews about cnidaria genomics have focused on the small number of species with sequenced genomes available at the time [11,23,24], on individual cnidarian lineages (i.e., Myxozoa [25]), or on specific topics such as toxins or evolution of novel traits [11, 26]. Given the increasing amount of genomic information available, an update of the state of Medusozoa genomics is warranted.

Here, we provide a comprehensive review of the major advances in Medusozoa genomics over the past century. To shed light on the understanding of the genomic evolution of the group from High-Throughput Sequencing (HTS) datasets, we report the main trends on the number and quality of available genome projects, taking into account basic information of sequencing datasets, genome assemblies, genome annotations, and accessibility of associated data and metadata.

## Methods

**Figure 2:**
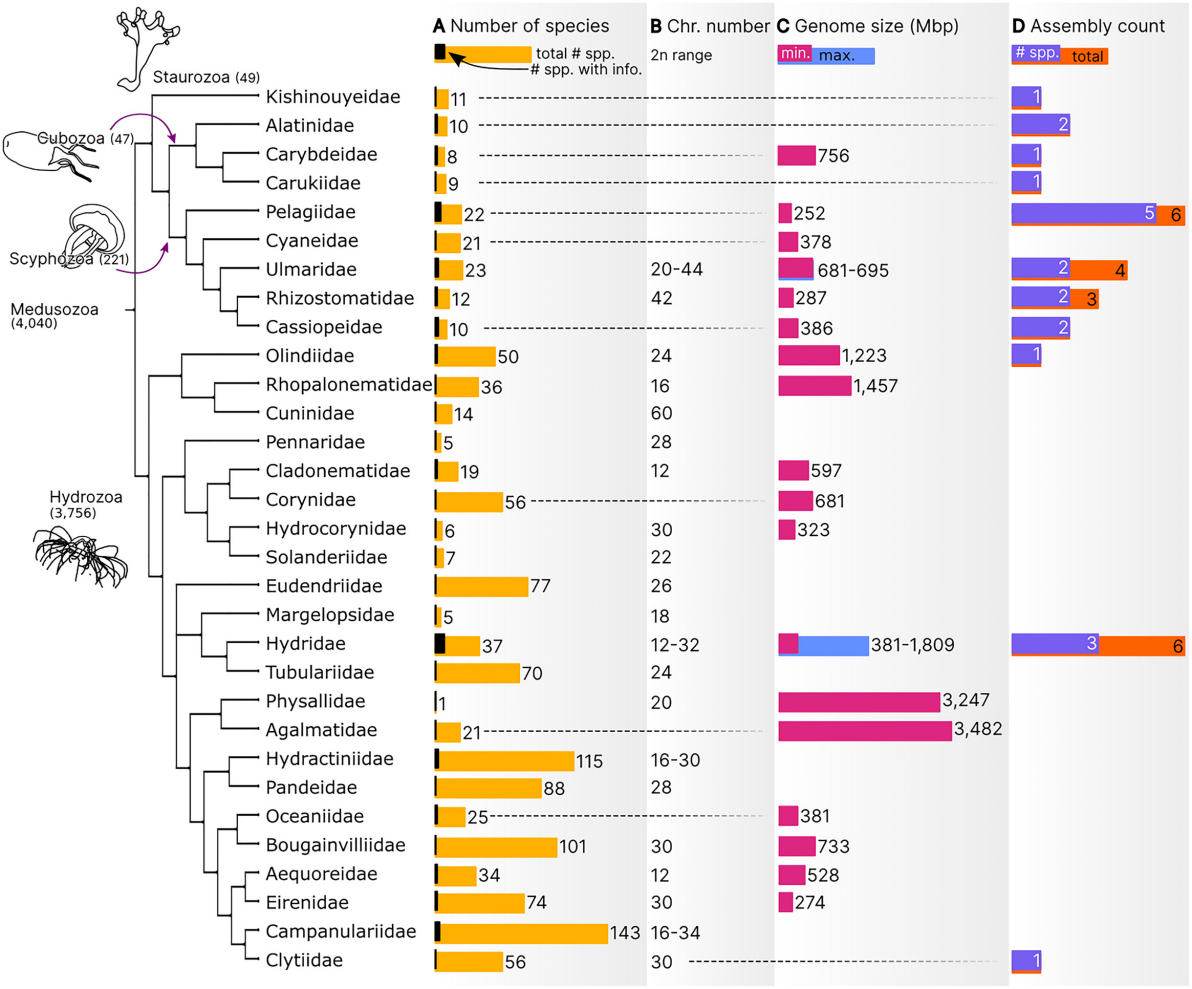
Phylogenetic distribution of genomic information in Medusozoa. (A) Number of described species and number of species with genomic data; (B) chromosome number (2n) range; (C) genome size (Mb) range taking into account flow cytometry and Feulgen densitometry estimations; (D) total number of available assemblies and number of species with assembled genomes. In (B) and (C) single values were also included when only 1 species was characterized. Tree topology is explained in the Methods section. Information used for this graph is available in Supplementary File S5 Table S2.

We surveyed literature and databases for cytogenetic reports and genome size estimations. Our main source of genomic information and metadata was NCBI Genome (Assembly, Genomes, Nucleotide, Taxonomy, and SRA [28]). We retrieved data automatically using entrez-direct v.13.9 and NCBI datasets v. 12.12. For information not present in NCBI, we checked published articles for proper information collection, as well as personal repositories mentioned in the associated articles. Owing to recent updates in taxonomic statuses, we modified the attribution of karyotypes, genome sizes, and assemblies of several species (see main text and Supplementary Materials). Assemblies identified as “preliminary” were not counted (Fig. 2 and Main Text) or reanalyzed, but were detailed in Table 1 and Supplementary File S1.

**Table 1:** Genomic projects related to Medusozoa HTS

Project	Release year (NCBI-SRA)	Class (No. genomes)	Species	Main research topics
Chapman et al. [40]	2008	Hydrozoa (1)	***Hydra vulgaris***	Gene evolution; micro-synteny
IISER Pune	2014–2015	Hydrozoa (1)	***Hydra vulgaris***	Hippo pathway; cell division; cell differentiation
NHGRI [41]	No SRA	Hydrozoa (1)	***Hydra vulgaris***	Regeneration, senescence; metazoan evolution; stem cells
NHGRI [42]	2016	Hydrozoa (1)	*Hydractinia echinata*	Stem cell biology; germ cell evolution; evodevo; evolutionary neuroscience
Gold et al. [19]	2018	Scyphozoa (1)	***Aurelia coerulea***	Life cycle; gene evolution; intraspecies variability; HOX
IRIDIAN GENOMES [43]	2018	Hydrozoa (1)	***Craspedacusta sowerbii***	Genomic documentation; comparative genomics
Kim et al. [44]	2018	Scyphozoa (1)	***Nemopilema nomurai***	Life cycle; jellyfish body patterning; gene evolution; toxins
IRIDIAN GENOMES [43]	2019	Hydrozoa (1)	*Scolionema suvaense*	Genomic documentation; comparative genomics
Khalturin et al. [20]	2019	Scyphozoa (1)	Aurelia aurita**, Aurelia coerulea**	Life cycle; jellyfish body plan; gene evolution; synteny
		Cubozoa (1)	***Morbakka virulenta***	
Leclère et al. [21]	2019	Hydrozoa (1)	***Clytia hemisphaerica***	Life cycle; gene evolution; micro-synteny; TF
Odhera et al. [22]	2019	Scyphozoa (1)	***Cassiopea xamachana***	Gene evolution; micro-synteny; Homeobox; toxins
		Cubozoa (1)	***Alatina alata***	
		Staurozoa (1)	***Calvadosia cruxmelitensis***	
Vogg et al. [45]	2019	Hydrozoa (1)	***Hydra oligactis*; *Hydra viridissima***	Gene evolution; RTKs; developmental genes
Hamada et al. [46]	2020	Hydrozoa (1)	***Hydra viridissima***	Symbiosis; immune response; repetitive DNA; Homeobox
IRIDIAN GENOMES [43]	2020	Cubozoa (1)	***Alatinidae* sp**.	Genomic documentation; comparative genomics
			***Carybdea marsupialis***	
			*Tamoya ohboya*	
		Hydrozoa (2)	*Cladonema radiatum*	
			*Eutima* sp. BMK-2020	
		Scyphozoa (4)	***Aurelia coerulea***	
			***Chrysaora achlyos***	
			***Chrysaora chesapeakei***	
			***Chrysaora fuscescens***	
		Staurozoa (1)	*Calvadosia cruxmelitensis*	
Li et al. [47]	2020	Scyphozoa (1)	***Rhopilema esculentum***	Gene evolution; toxins
Nong et al. [48]	2020	Scyphozoa (2)	***Sanderia malayensis, Rhopilema esculentum***	Gene evolution; small RNAs; micro-synteny; Homeobox
Xia et al. [49]	2020	Scyphozoa (1)	***Chrysaora quinquecirrha***	Gene and gene feature evolution; repetitive DNA
Xia et al. [50]	2020	Scyphozoa (1)	***Chrysaora quinquecirrha***	Assembly improvement report
UMCG	2021	Scyphozoa (1)	***Cassiopea andromeda***	Venom; toxins; evolution

Sequencing projects with no current related publication are remarked with capital letters. Column “Main research topics” describes keywords according to references, restricted to a maximum of 4; “gene evolution” refers to the study of gene gains/losses and also of specific gene families. Species with reported assemblies were re-analyzed in this review (boldface; Supplementary File S5 Table S3). IISER PRune: Indian Institute of Science Education and Research, Pune; NHGRI: National Human Genome Research Institute; RTK: receptor tyrosine kinase; TF: transcription factors; UMCG: University Medical Center Groningen; **species with taxonomic updates. For further details see Supplementary File S1.

Because there have been subtle variations in metrics and statistics between most genome reports, we recalculated some statistics, allowing us to make meaningful comparisons. Briefly, we have generated the following: (i) assembly statistics using the statswrapper.sh script from BBmap v38.73 (BBmap, RRID:SCR_016965) [29]; (ii) gene statistics from the original annotation files with AGAT v0.6.0 [30] and assessment of completeness of all assemblies using BUSCO v5.0.0+galaxy0 (BUSCO, RRID:SCR_015008) [31] in genome mode and Metaeuk software, using 2 Single Orthologs Databases (eukaryota_odb10, number of genes = 255, number of species = 70; metazoa_odb10, number of genes = 954, number of species = 65), available at the public Galaxy server [32,33].

Assembly quality was reported following the metric proposed by Earth Biogenome Project [34] (hereafter BGP-metric). This system avoids the use of ambiguous terminology for quality and uses a logarithmic scale where the first 2 numbers are the exponents of the N50 contig and scaffold (1: 0–99 kb; 2: 1–9.9 Mb; 3: 10–99.9 Mb) and the third number corresponds to the level of chromosomal assembly (1: >90% DNA assigned to chromosomes *in silico*; 2: chromosomal rearrangements validated by 2 data sources; 3: >80% DNA assigned to intra-species maps and experimental validation of all breakpoints; see [34]).

All graphs were generated using Python v.3 with ETE Toolkit v.3 [35], Matplotlib v3.3.1 [36], and Seaborn v.0.11 [37] and modified with Inkscape v.0.92 [38] to improve visualization (e.g., font size and spacing). The tree of Figs2 and 4 represents a simplified phylogenetic hypothesis obtained by combining phylogenies from previous studies (Scyphozoa [39], Medusozoa [5], Hydrozoa [51, 52]), taking into account clades with high congruence and support values. Although the different phylogenetic hypotheses were mostly congruent, no single study nor molecular dataset comprised all the terminals discussed here. We manually compiled all genomic information and HTS metadata referenced in this review using a report model based on previous works and public databases such as NCBI (Supplementary File S1 [30,53, 54]). The command line used for retrieving genetic information and metadata, for statistics calculation, and the code used for graph generation are available at Supplementary Files S2 and S3. All collected data were updated until 1 May 2021.

## Genomic Projects: Whos and Hows of Medusozoa

Chromosome numbers are known for 34 hydrozoan species and 5 scyphozoan, including 3 lineages of the *Aurelia aurita* sp. complex species ([12,13,21,55–63]; Supplementary File S4). Older chromosome descriptions for 25 species do not include information about chromosome morphology and often lack photographic records or schematic representations [12,13, 55–59].

Genome size, a fundamental feature in any genome sequencing project, has been experimentally estimated by flow cytometry or Feulgen densitometry techniques for 24 medusozoan species (Scyphozoa: 7 spp.; Cubozoa: 1 sp.; Hydrozoa: 16 spp.; Supplementary File S4). Genome sizes are highly variable, ranging from 254 to 3,481.68 Mb in *Sanderia malayensis* (Scyphozoa) and *Agalma elegans* (Hydrozoa), respectively [15]. Moreover, an additional 12 genome size estimates are available when considering *k*-mer–based computational assessments, increasing the number of species with genome size information to 30, and including 2 cubozoans (913–2,673 Mb) and 1 staurozoan (230 Mb) (Supplementary Files S1 and S4). These estimates are considered less accurate, especially for genomes with high heterozygosity, high repetitive content, and large genome size [64]. In fact, *k*-mer–based and experimental estimations from the same species differed by 13–33%.

A total of 34 HTS projects were identified. Of these, 32 had sequencing reads accessible through the NCBI-SRA database, but not all of them were associated with a genome assembly (Table 1, Supplementary File S1). The taxonomic coverage of the assemblies encompassed 7 of the 13 Medusozoa orders and represented ≥1 species per class (Fig. 2): 28 assemblies were accessible for 21 species, representing 0.5% of Medusozoa (Fig. 2, Table 1, Supplementary File S1). Of these 21 species, 12 were Scyphozoa, 4 were Hydrozoa, 4 were Cubozoa, and 1 was Staurozoa. Scyphozoa had the highest number of sequenced families (4 of 22), of which Pelagiidae contained the highest number of sequenced species so far (5 spp.), followed by Ulmaridae, Rhizostomatidae, and Cassiopeiidae with 2 spp. each (Fig. 2), all belonging to subclass Discomedusae (none from Coronamedusae). The remaining assemblies represent 3 of the 8 Cubozoa families and 3 of 135 Hydrozoan families (Fig. 2). In addition to the small fraction of family representation in the hydrozoan genomes, the underrepresentation of Leptothecata is particularly unfavorable because it harbors more than half of Medusozoa species (2,059 spp. [1]).

Much of the assembly effort is biased towards a small number of species. For example, 3 species of Hydrozoa and Scyphozoa presented 2 assemblies each, of which *Hydra viridissima* and *Rhopilema esculentum* were sequenced twice independently; meanwhile *Chrysoaora quinquecirrha* presents 2 versions of the same assembly. Moreover, 3 assemblies were available for 2 different strains of *Hydra vulgaris* (former *Hydra magnipapillata*), 1 of them published as an update of the reference genome called Hydra 2.0. In *Aurelia*, the genomes of 3 different lineages were sequenced and assembled: Baltic sea, Roscoff, and *Aurelia* sp1. strains [19,20]. Based on a recent taxonomic update of this genus [65], locality and genetic information described in the original articles [19,20], we decided to refer to these genomic datasets as Baltic sea strain = *Aurelia aurita;* Roscoff strain and *Aurelia* sp1. strains = *Aurelia coerulea*.

Most of the assemblies were deposited in the NCBI Assembly database, 1 was only found in a journal-specific database (i.e., GigaDB [66]), 1 assembly was only in a personal repository (Google Drive), and 1 in the National Human Genome Research Institute site [41]. Some assemblies were additionally deposited in institute-centered repositories such as OIST Marine Genomics Unit [67] and the Marine Invertebrate Models Database (MARIMBA [68]). A significant portion of the publicly available assemblies (total of 8, ∼30%) are not yet associated with a formal publication and belong to the IRIDIAN GENOMES project [43]. The most frequent sequencing technology was Illumina (26 assemblies, ∼93%), but leaving aside unpublished ones, most works include a combination of different sequencing techniques, library sizes, and platforms (i.e., Sanger, 454, Illumina, long reads, linked reads, and Hi-C sequencing; Supplementary File S1).

**Figure 3:**
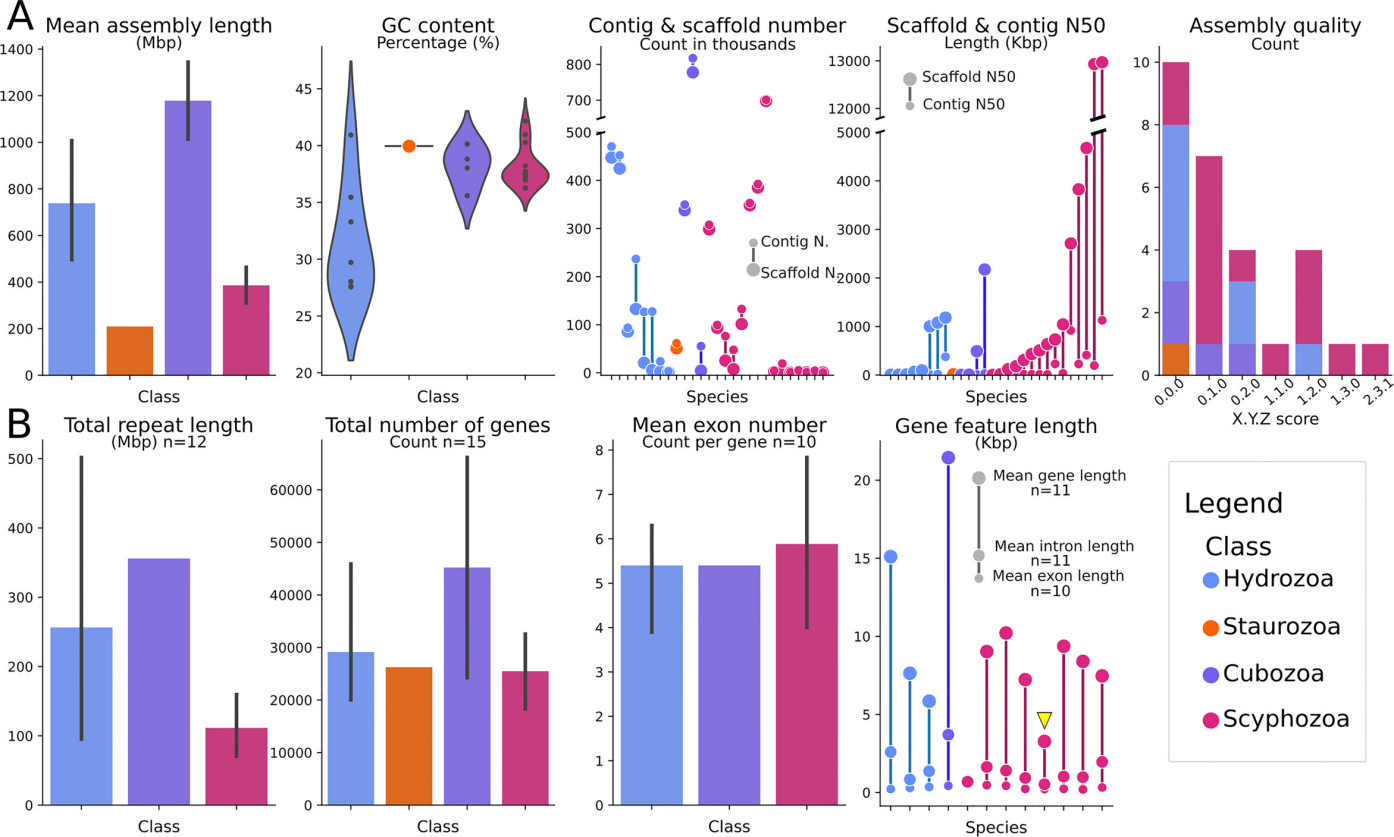
Assembly and genome features. (A) (from left to right): mean assembly length per class, GC content (%) per class, number of contigs and scaffolds per assembly colored by class, contig, and scaffold N50 (in kb) per assembly coloured by class, and count of assemblies of each class corresponding to the different BGP-metric values, where X and Y correspond to contig and scaffold N50, respectively, and Z to chromosome assignment (see Methods section). (B) (from left to right): mean repeat length (Mb) in assembly per class, mean total number of genes per class, mean exon number (count per gene) per class, and mean gene, intron, and exon length (kb) per assembly colored by class. The yellow arrowhead indicates *S. malayensis* gene features (see Box). Within the dotplots, a data point is indicated for each species. When more than one species per class was available, vertical lines were added to barplots to indicate the value dispersion around the mean. All other keys are specified in the figure. Information used for this graph is available in Supplementary File S5 Tables S4–S6.

Almost all medusozoan genome assemblies were at draft contig or scaffold level, with 1 exception, *R. esculentum*, where chromosome-level scale assembly was reported [47]. The total length, contig and scaffold number, N50, and GC% varied across species and classes (Fig. 3A; references in Supplementary File S5). The assembly continuity and quality was higher in Scyphozoa than in the other classes, as observed by the distribution of contig and scaffold N50 (Fig. 3A) and the BGP-metric for assembly quality (Fig. 3A). In general, they are fragmented (75%) and have contig N50 of <40 kb (Fig. 3A; BGP-metric values of 0.0.0, 0.1.0, and 0.2.0). Staurozoa, Cubozoa, and Scyphozoa assemblies have similar percentages of base composition, ∼35–43% GC. Consistent with previous reports [69], Hydrozoa genomes have a higher dispersion of GC%, with the GC values of 5 assemblies <35%.

In relation to gene content (Fig. 3B), 17 genomes were annotated using ≥1 source of information (Supplementary File S1) and their total number of genes or total number of protein-coding genes were reported. Further description of coding information was variable among works, and as more detailed information was considered, the number of genomes with reported information decreased. Annotation tracks and gene models were available for only 11 of the 17 datasets. Recalculations of gene features, together with the information recovered from original articles, allowed us to analyze the distribution of 5 different features in 15 genomes of Scyphozoa, Hydrozoa, and Cubozoa (Fig. 3B; Box): number of genes (n = 15), mean exons per CDS (n = 10), mean gene length (n = 11), mean exon length (n = 11), and mean intron length (n = 12). For 3 species, *Cassiopea xamachana* (Scyphozoa; 31,459), *Alatina alata* (Cubozoa; 66,156), and *Calvadosia cruxmelitensis* (Staurozoa; 26,258), the available information was restricted to the number of predicted genes. Some small inconsistencies were detected between original data reported in some articles and our recalculations (Table S5 and S6), and others between data reported in the main text and supplementary materials of some articles.

The determination of repetitive DNA has been an integral step before gene annotation in most genomic projects. Frequently, repeat diversity was not properly reported and the degree of detail also varied between articles: e.g., some published works only referred to the most abundant class of repetitive DNA, meanwhile others described only results at class or family level. Repetitive libraries—consensus sequences representing repeat families—were not properly saved in repositories with the exception of 2 independent articles, and RepeatMasker results were reported in 4 articles (1 reporting only classified repeats). Total repetitive length of 12 species for which coding information was also available is presented in Fig. 3B and discussed in the Box.

**Figure 4:**
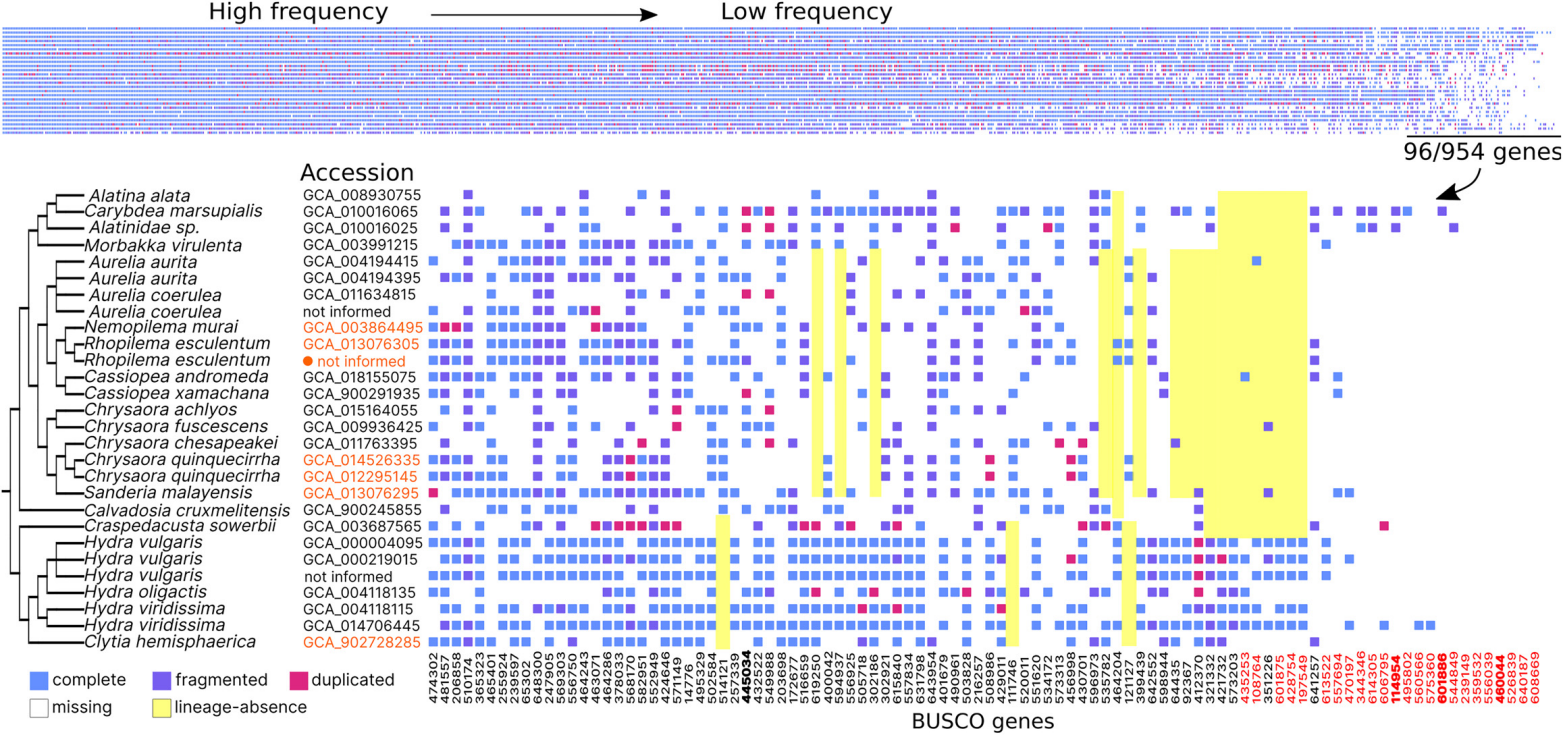
BUSCO Metazoa gene distribution in Medusozoa assemblies. Each column corresponds to a gene and each row an assembly. Columns were ordered based on presence from left to right and the least present genes (n = 96) are shown in detail. Genes absent in all or almost all assemblies (>80% of absence) are indicated in red; genes also reported absent [20] are indicated in boldface; genes absent in specific lineages are indicated with yellow rectangles. Higher quality assemblies are indicated in orange (BGP-metric > 1.0.0). The assembly with the highest quality score for BGP-metric is indicated by an orange circle and corresponds to *Rhopilema esculentum* [47]. Information used for this graph and full BUSCO gene names are available in Supplementary File S5 Table S7.

The degree of completeness of these datasets also varied substantially, as estimated by BUSCO (metazoa_odb10 and eukaryota_odb10; Fig. 4). While all Eukaryota genes were present in ≥1 assembly (Supplementary Files S5 and S6), the level of absence and fragmentation of Metazoa genes was higher (Fig. 4; Supplementary File S5). Seven Metazoa genes were absent in all assemblies and 17 were absent in >20% of them (Fig. 4, indicated in red). Some Metazoa BUSCO genes were absent in lineages with the higher number of assemblies, such as Scyphozoa and Hydrozoa (Fig. 4, indicated in yellow rectangles; Supplementary File S5). This condition was suggested by [20], after detecting the absence of 14 genes in 5 species (version metazoa_o9db), 3 of which coincided with the genes detected as absent here (Orthodb IDs: 460044at33208, 601886at33208, 114954at33208), 1 of which (445034at33208) has a patchy distribution in Medusozoa and 9 of which were removed in later versions of the database (Fig. 4 in boldface).

Moreover, 27 genes were simultaneously recovered as undetectable or fragmented in >80% of the assemblies (Supplementary File S5 Table S7). Based on BUSCO completeness assessment with metazoa_o10db, 13 assemblies present 90–95% of genes (fragmented + complete), while only 1 assembly includes >90% of complete genes; the remaining 15 assemblies present between 57% and 87% of genes (complete + fragmented) or 16–77% complete genes. While the Metazoa database might include genes that are absent, fragmented, or have non-conventional features in all medusozoa species, the utility of the Eukaryota database in the completeness assessment is limited by its low number of genes. Until more specific databases are developed, the combination of both BUSCO databases should be used taking into account their limitations.

Differences in sequencing strategy and platforms are expected to be linked with assembly quality, in terms of both continuity and completeness. For example, hybrid sequencing plus proximity ligation maps and combined evidence-based annotation should generate better results than a short-read sequencing and single-evidence annotation [70, 71]. Although this general trend was observed in this review, with most Illumina-only datasets showing lower BGP-metric (Fig. 3) and lower completeness (Fig. 4), it is not a granted condition. Certain specific cases can exemplify biological and methodological issues that impose limitations to genome sequencing and assembly: e.g., the difficulty in obtaining chromosome-scale assemblies despite small genome sizes and combined sequencing strategies (Hi-C + short reads + long reads) [48,50] or the difficulty in extracting high molecular weight DNA [20]. Because of the heterogeneity of Medusozoa genomic projects in terms of time periods, objectives, methods, and resources, a proper quantitative analysis of the relationship between methods and outcome quality would not be feasible, and we prefer to refer to articles specialized in assessing methods (e.g.,[70, 71]).

## The State of Medusozoa Genomics: Inner and Derived Knowledge

The first glimpse of the Medusozoa genomic organization was obtained by cytogenetic studies [12,13, 21, 55–63], but in contrast to other animals, the available information is still sparse. Many cytogenetic questions essential to the understanding of genome evolution are unanswered in Medusozoa, either at species or population scale, including the distribution of the chromosome number (2n), fundamental number of chromosome arms (FN), genome size, ploidy level, and heterochromatin content. These are questions that have gained renewed interest since the arrival of the genomic era.

Regarding the phylogenetic distribution of the chromosome number, no inferences can yet be made on the sparse available information, apart from the presence of some chromosome variation throughout Medusozoa. A special case was reported in *Hydra*, where, according to recent descriptions, many species shared a 2n = 30 karyotype with metacentric or submetacentric chromosomes ([63]; Supplementary File S4). This suggests that the 2n = 30 karyotype could be widely distributed in the genus and even in other Hydrozoa groups because it was also described for 1 species of Hydrocorynidae, Hydractiniidae, Campanulariidae, Bougainvilliidae, and Clytiidae, and 3 Eirenidae (Supplementary File S4; references therein). Interestingly, in Anthozoa, a few sea anemones and several scleractinian corals have karyotypes between 2n = 28 and 2n = 30 [72–74]. Nevertheless, a higher sampling effort should be conducted to test the extent of this apparent karyotype stability.

Scyphozoa genomes tend to be smaller (∼250 to ∼700 Mb) than those of Hydrozoa, which encompass a larger range (∼380 to ∼3,500 Mb) (Fig. 2; Supplementary File S4, references therein), but owing to the scarcity of estimations that represent ∼1% of the subphylum, these ranges should be considered preliminary. The evolution of eukaryotic genome size is a long-standing question that has been called the “C-value enigma” [53]. This name stems from the difficulty elucidating the evolutionary forces (e.g., drift and natural selection) that have given rise and serve to maintain variations in genome size, the mechanisms of genome size change, and the consequences of these variations at an organismal level [53]. Several conflicting hypotheses have been postulated to explain this puzzle, with most having experimental support in some but not all lineages (reviewed in [75]). The molecular basis of these variations in Medusozoa has only been studied in detail for *Hydra* [76] and for *S. malayensis* [48]; their trends have been related to repetitive DNA and gene length, respectively (Box). Meanwhile, the ecological and historical factors underlying genome size diversity and its extent in Medusozoa are topics that remain to be elucidated.

Box. Genome content

- **Gene content and length:** it is straightforward to imagine that the evolution of these 2 characteristics has potential impacts in macroevolution of organisms. The distribution of gene number in Medusozoa (Fig. 3B) ranged from 17,219 in the Scyphozoan *Rhopilema esculentum* [47] to 66,156 in the Cubozoan *Alatina alata* [22], which is higher than the range (18,943 ± 451.82) described for animals [42]; however, most species of all classes have gene counts near the median (26,258). The upper limit described in the highly fragmented *A. alata* genome deviates from that observed in *Morbakka virulenta (*24,278 genes), the only other sequenced Cubomedusae [20,22]. Species with varying genome sizes of Hydrozoa, Scyphozoa, and *M. virulenta* (Cubozoa) had similar mean CDS lengths (1,414, 1,214, 1,387 bp), mean numbers of exons per gene (5, 6, 5.4), and mean exon lengths (306, 293, 432 bp) but had different gene lengths (9,530, 7,855, and 21,444 bp, respectively) owing to the presence of longer introns in Hydrozoa and Cubozoa when compared to Scyphozoa (Hydrozoa: 1,600; Cubozoa: 3,705 vs 1,146 bp in Scyphozoa). This is best exemplified in the genome of the scyphozoan *S. malayensis*, which has the smallest cnidarian genome reported to date [48] and has also the smallest introns of any sequenced medusozoan genome (Fig. 3B, yellow arrowhead). Nevertheless, these ranges are rough estimates and sometimes heterogeneous, e.g., resulting from different filtering parameters, and their implications should be tested as new assemblies and annotations become available.
- **Repetitive content:** repetitive DNA represents a significant part of eukaryotic genomes and is highly diverse, composed by different kinds of transposable elements (TEs), tandem repeats, and multigene families (e.g., rRNA and tRNA). Many of these sequences, especially TEs and satellite DNA, were initially considered as an expendable sector of the genome, although their impact on genomic evolution has since been recognized (reviewed in [77]). For example, fusions between TEs and host genes have occurred multiple times in vertebrates and have contributed to the evolution of novel features [78]. Likewise, TEs and other repetitive DNA have been associated with genomic rearrangements and changes in DNA content (e.g., [76, 77]). The *Hydra* genus, which has been more extensively studied from this point of view, has experienced a rapid genomic evolutionary rate and presents a 3-fold genome size increase resulting from the amplification of a single long interspersed nuclear element family [76]. Moreover, *Hydra* genomes include an over-representation of transposase-related domains [46]. It is interesting to note that many of the Medusozoa species studied so far have relatively small genomes but unusually high proportions of repetitive DNA [20,44,48, 49]. Nevertheless, the lack of standardization in the description of its diversity, and the discrepancy in the degree of detail in which these have been described, limits the potential to make inferences. Repetitive DNA is a complex study subject, limited by assembly continuity and annotation effort, but restricting genomic studies to the “functional” part of the genome (sensu [79]) may lead us to a narrowed view of the Medusozoa genome evolution.

Modern Medusozoa genomics formally started with the sequencing and publication of the *H. vulgaris* genome, which in Cnidaria was only preceded by *Nematostella vectensis* [40,72]. *H. vulgaris* is one of the earliest models in biology, mainly used for the study of development, regeneration, and more recently, of aging (reviewed in [80, 81]). The study of these 2 early genomes was fundamental for the reconstruction of a more complex ancient eumetazoan genome than first suggested by the comparison of vertebrates and insects [16,23,40,72].

Unlike most other medusozoan species, *Hydra* lives in fresh water, lacks a medusa, and has a genome that has experienced a very rapid rate of evolution [21]. It therefore is not the ideal species for reconstructing historical nodes on the Medusozoa tree of life. As such, more recent medusozoa genomes have led to important updates in our understanding of Medusozoa-relevant research topics, including phylogenetic reconstructions, the genetic basis of the medusae, the evolution of symbiosis, toxin characterization, and Homeobox gene evolution, to name a few examples (Table 1). Nevertheless, Medusozoa genomes include thousands of single-copy genes and repetitive elements; however, only a limited number of them have been analyzed in detail.

The complex nature of Medusozoa venom has been investigated by a number of transcriptomic, proteomic, and genomic studies (reviewed in [26]). Several putative toxin genes and domains have been identified, covering a significant part of the wide range of known toxins [20,22,44,47]. In Scyphozoa, toxin-like genes were often recovered as multicopy sets [20,47]. Moreover, in *R. esculentum* toxin-like genes were also tandemly arranged and several of them were located nearby in chromosome 7, suggesting that the observed organization might influence toxin co-expression [47]. Minicollagens, which are major components of nematocysts, also had a clustered organization and a pattern of co-expression in *Aurelia* [20]. These examples add to various clustered genes described in Cubozoa, Hydrozoa, and Anthozoa and would indicate that gene clustering and operon-like expression of toxin genes is widespread in Cnidaria ([20] and references therein).

The determination of lineage-specific genes and increases and decreases of gene content is one of the recurrent questions found in Medusozoa genomic studies (e.g., [20,21]), and it has been conducted using different methodologies and sets of species. Recent evidence proved that the detection of lineage-specific genes, and other analyses relying on accurate annotation and orthology prediction, can be significantly biased by methodological artifacts [82–86]; several problems have been identified, such as low taxon sampling, heterogeneous gene predictions, and failure of detecting distant homology and fast-evolving orthologues. These considerations are highly relevant in Medusozoa because comparisons are often made, by necessity, with distantly related species (e.g., Anthozoa has been estimated to have diverged from Medusozoa ∼800 million years ago [87]). In Cnidaria, the most elevated rates of loss have been estimated in the hydrozoan branch leading to *Clytia hemisphaerica* and *Hydra* [21, 40], followed by slightly lower rates of gene loss in Scyphozoa and substantially lower rates in Anthozoa [19]. Gene families that have experienced expansion and contraction have been studied in relation to complex life cycle patterns [19,21], simplification of the body plan [40,46], and the evolution of symbiosis [46], among others (Table 1). Expression patterns of identified taxonomically restricted medusozoan genes have been mainly studied in the context of life cycle stages (e.g., [20,21]).

The complex life cycle of Medusozoa has resulted from the combination of both ancestral and novel features. *Aurelia, Morbakka virulenta*, and *C. hemisphaerica* have significantly different patterns of gene expression across stages and during transitions [19–21]. Differentially expressed genes include many conserved ancestral families of transcription factors [19–21]; there is also a considerable amount of the putative lineage-restricted genes that show differential expression in the adult stages [20,21]. A few of these “novel” medusozoan genes have been described, such as novel myosin-tail proteins that are absent from Anthozoa and represent markers of the medusae striated muscles [20]. It was suggested that the evolution of the Medusozoa complex life cycle would therefore have involved the rewiring of regulatory pathways of ancestral genes and the contribution of new ones [19–21]. As such, the body plan and life cycle simplifications observed in Clytia and Hydra, respectively, would be the result of loss of transcription factors involved in their development [21]. Finally, the significance of many of the putative Medusozoa and species-specific genes remains to be elucidated.

On the other hand, synteny was also analyzed several times, including species of Hydrozoa, Cubozoa, and Scyphozoa, and analyses were carried on at different scales depending on assembly continuity (i.e., microsynteny and macrosynteny), and often comparing the focus species to species from sister clade Anthozoa [19–21,40, 77]. High synteny conservation was found within Anthozoa (*N. vectensis* vs *Scolanthus callimorphus* [72–74]) and within Hydrozoa (*H. vulgaris* vs *C. hemisphaerica* [21]). Meanwhile, conservation of synteny at a lesser degree was also observed between Anthozoa and Scyphozoa (*N. vectensis* vs *R. esculentum*; *N. vectensis* vs *Aurelia* strains [19, 20,74]) and only a few shared syntenic blocks between Hydozoa and Anthozoa (*H. vulgaris* vs *N. vectensis* [21,40,74]), Hydrozoa and Scyphozoa (*H. vulgaris* vs *A. aurita* [19]), and Scyphozoa and Cubozoa (*A. aurita*vs*M. virulenta* [20]). It is particularly interesting to note that *H. vulgaris, N. vectensis*, and *S. callimorphus* present 2n = 30 but shared fewer syntenic blocks than either of the 2 anthozoans with *R. esculentum*, which has a different karyotype (2n = 22) ([74] [non peer-reviewed]). These results suggest that there is evidence for the conservation of an ancient genome architecture in Anthozoa and Scyphozoa, but less conservation in Hydrozoa and Cubozoa, coincident with a more rapid rate of genome reorganization in the last 2 classes [21,74].

## Prospects on Genomic Data and General Resources

The increasing amount of genomic information available for diverse organisms has enabled statistical inferences of trends in eukaryotic genomic evolution. Examples of such studies are available at small and large phylogenetic scales and have enabled evolutionary analyses of the distribution of gene numbers, gene features (e.g., intron size), and repetitive content (e.g., [53]). Nevertheless, the power of eukaryotic genomic comparative analyses is hindered by a lack of data and metadata standardization [53, 88], which is especially evident in Medusozoa.

There is much to learn from decades-old references of cytogenetic studies, but some studies, especially older ones, lack complete material and methods (e.g., pretreatment, references, designs and photographs; general metadata as locality, taxonomic identification) and therefore should be considered carefully in a comparative framework (e.g., [89]).

Similar problems can be expected in relation to genomic data because metadata are often not specified in great detail. We analyzed hundreds of fields including genetic information and metadata (methods, metrics and registry codes; table in Supplementary File S1), of which no dataset presents most of them, whatever the area or section (e.g., processing area, section trimming). This could be a future problem because reusing previously published datasets is becoming routine, and tracking of information (e.g., BioProjects, Biosamples, methodologies, filtering parameters) would be misleading [88, 90].

Descriptions of bioinformatic methods in genome studies are often even less comprehensive than database metadata. For example, we identified ≥3 independent projects, each of which applied different criteria for gene model filtering, and another 3 articles applied slightly different criteria for repeat library filtering (Supplementary File S1). Although differences at this stage can seem small on the surface, they can result in hard-to-detect biases downstream that can lead to flawed biological conclusions. For example, resistance genes have been underestimated in some flowering plant genomes owing to inconsistencies of genome annotation stemming from differences in repeat masking [91]. Likewise, in the present review, we identify discrepancies in BUSCO genome completeness comparisons that are caused by differences in database versions, which are frequently unspecified in the associated articles.

An alternative solution for comprehensive comparative analyses is to (re)annotate all genomes with the same pipeline, a task that is laborious and time consuming. Some programs were designed for achieving this task simultaneously in many related species (e.g., [92, 93]). Another alternative is to use specific software developed to improve genome annotations by leveraging data from multiple species (e.g., [94, 95]) or targeting specific gene families [96, 97]. Finally, differences in annotation due to methodological artifacts can be accommodated in comparative analysis if considered as a variable in the statistical tests (e.g., comparing tRNA genes in high- and low-quality avian genomes [98]).

The submission of raw sequencing data and fundamental metadata to the NCBI-SRA or EMBL-ENA remains a vital step in ensuring the usability and transparency of genome data [99, 100]. Also, project-centric repositories serve to store assemblies and associated datasets, and enable comparative studies by basic tools. Taxon-restricted databases including cnidarian data have been used in the past, but these are often not maintained owing to lack of upkeep funding and other factors (e.g., [101, 102]). In addition, submission to the large databases like SRA and GenBank can lead to the automatic detection of specific issues such as contamination or annotation errors that might otherwise not be detected. For these reasons, the large general databases should remain the primary repositories for sequence and metadata [103]. Nevertheless, this is not always the case. For example, the assembly with the highest continuity as estimated by the BGP-metric, corresponding to *R. esculentum* [47], is only found in a journal-specific database and lacks a stable identifier (e.g., NCBI accession). A similar situation is observed for 1 of the *H. vulgaris* assemblies (Hydra 2.0), which is only found in a project-specific database [41].

There is a growing number of community-driven guidelines, standards, databases, and resources based on the Findable, Accessible, Interoperable, and Reusable principles (FAIR principles) for digital research outputs [103]. Furthermore, global initiatives of large-scale genome sequencing included in Earth Biogenome Project have adopted a set of standardized protocols for the different stages of the genome projects, such as specimen collection, DNA extraction, sequencing, assembly and annotation methods, and reporting, in order to generate datasets that could “be useful to the broadest possible scientific community” [34]. Standards should also be implemented by independent research groups publishing genomes. The main goal of standardization is to promote evaluation, discovery, and reuse of genomic information, providing long-term benefits for science.

The following are suggestions to enhance genome projects and outcomes, and to promote open and collaborative research. These suggestions can be broadly applied to any genome project and are in line with those proposed by many initiatives and consortia (e.g., [34, 103, 104]). Nevertheless, it is worth reinforcing and discussing them in the context of this review because genome projects are more and more often being initiated in research laboratories that have historically been more focused on other aspects of medusozoan biology and may not be as familiar with these general practices:

1. Deposit all data and metadata in public specialized databases (e.g., NCBI), at least once associated articles are accepted for publication. Provide comprehensive metadata, including those not considered as priority for the aforementioned project. Frequently, data and metadata that are described in the original articles or deposited in repositories are not submitted to public databases. Tracking information from multiple sources is time consuming and prone to error. Databases and repositories enable the improvement of metadata after the initial releases, by the addition of new or corrected information (e.g., publication information) from the authors. We believe that this kind of data curation would improve the state of Medusozoa genomics not only by enabling downstream analysis after the publication but also by enabling the detection of methodological options (e.g., tissue selection; sequencing technology) that would improve the quality of the results.
2. Consider providing standardized genome statistics in an easily accessible format (e.g., Supplementary File S1 presented here). Alternatively, use specialized tools that standardize reports for multiple samples and datasets (e.g., [54,105, 106]). This will facilitate meta-analyses, prompt new genome studies to make accurate comparisons to previously published studies, and prevent the propagation of erroneous information.
3. Deposit output results that were fundamental in any of the steps of the analysis (e.g., gene models, repetitive libraries, and annotation tracks). A Medusozoa-centric database with long-term maintenance is still lacking for the community (e.g., Mollusca clade [107]), but many open repositories can serve this purpose with low or no costs considering the size of the aforementioned outputs. There are open topic-centric repositories (e.g., Dfam [108] for repetitive DNA), general repositories (e.g., FigShare, Zenodo; or even NCBI for annotation tracks), as well as personal or institutional ones. Many of the reviewed genomic projects already made use of these repositories but failed to deposit some of the outputs. A solution for this inconvenience is to update submissions or create novel ones (e.g., submit annotations to NCBI or ENA) to deposit the missing outputs.
4. Inform as much as possible if a dataset was edited (e.g., removal of exogenous DNA; gene and repetitive sequence filtering criteria).
5. Use and clearly identify software, database versions, and references in all instances (e.g., RRID, BUSCO version, and repetitive database version).
6. Deposit command lines and scripts used to handle data (from reads to full annotation).

The latter suggestions (3–6) are mainly related to providing detailed methodologies of bioinformatic analyses. First, proper method and results descriptions can help to recover metadata and criteria usually not available in large sequence repositories. Second, comparative analyses depend upon standardization at different levels and significant sample sizes. The inclusion of species in downstream analyses is limited by data availability and proper description of previous analyses, custom software, and results.

7. Engage in community-driven conversations about standards, guidelines, and species priorities. There are a number of taxon-specific meetings that would be appropriate venues to engage in these conversations including the International Conference on Coelenterate Biology (∼decennial [109]), the International Jellyfish Blooms Symposium (∼triennial), Cnidofest (∼biennial [110]), Tutzing workshop (∼biennial [111]), and Cnidofest zoom seminar series. In addition, satellite meetings at larger annual meetings (e.g., the Society for Integrative and Comparative Biology [SICB] or the Global Invertebrate Genomics Alliance [GIGA] [104]) could provide appropriate venues to facilitate discussions on how the community can best move forward as more and more genomic data come online.

The adoption of best practices in the Medusozoa genomics community will pave the way for major breakthroughs regarding understanding the genomic basis for several evolutionary innovations that arose within and in the stem lineage of Medusozoa. Similar advances were achieved with extensive taxon sampling at broader scales, where 25 novel core gene groups enriched in regulatory functions might be underlying the emergence of animals [112, 113]. Medusozoa innovations have puzzled the community for decades [5, 7,11,114] and include the origin of the medusa, the loss of polyp structures, the establishment of symbiosis, the blooming potential, and the evolution of an extremely potent venom. A deeper understanding of the genomic events driving these innovations will require accurate identifications of a number of key genomic features including (but not limited to) single-copy orthologs, gene losses, lineage-specific genes, gene family expansions, and non-coding regulatory sequences.

## Conclusions

The pace of genomic development in Medusozoa is far more rapid than more traditional disciplines such as cytogenetics, where gaps still remain. As the effect of chromosome structural variants in evolution is increasingly tested and recognized, it is expected that these disciplines will gain a revived interest as has been seen in other animal groups [115]. In spite of the great advances in Medusozoa genomics, we found a general lack of standardization in methodologies and genome reports across independent sequencing projects. Efforts to incorporate standards would benefit future studies and could promote the identification of hitherto undiscovered evolutionary patterns.

It is safe to anticipate that standardization will become increasingly easier as chromosome-level assemblies become more commonplace and as new integrated workflows of data reporting and submission are developed (e.g., [116]). It will be possible to perform standardized annotation and analyses to identify patterns in medusozoa genome evolution.

The distribution of genetic and genomic information presented significant taxonomic gaps in Medusozoa. It is a reasonable scenario because genomic sequencing data are accumulating in many medusozoan lineages. Even so, some of the most species-rich clades with a diverse array of phenotypic and ecological traits have not yet had their genomes sequenced (e.g., Scyphozoa:Coronamedusae, Hydrozoa:Macrocolonia). These, and other, heretofore genomically underexplored lineages provide golden opportunities from which to make major contributions to understanding the evolution of Medusozoa genomes and would be a wonderful contribution to the rest of the Medusozoa research community. Defining candidate species for sequencing can avoid unnecessary doubled efforts. Different international projects recognized this situation and proposed a set of criteria for prioritizing species at other scales, such as the GIGA ([104]).

Conversations about how best to promote such efforts and best practices for medusozoan genomics will help move the field forward. Such conversations could lead to new standards and potentially a powerful cnidarian genomics database. This latter goal would be most effective if accompanied by a strong alliance that spans the growing cnidarian genomics community.

## Data Availability

All collected information, outputs, and scripts supporting new results are available in the Supplementary Files S1–S9 in Figshare [117]. All genomic resources from previous articles and projects are publicly available and their sources are referenced in Supplementary File S4 Table S3. The most up-to-date copy of Table S1 is available in Figshare [117] and can be updated upon the original author's request.

## Additional Files

Supplementary File S1. Dataset 1. Genome report sheet.

Supplementary File S2. Dataset 2. Command line to retrieve data from NCBI and to generate new results.

Supplementary File S3. Dataset 3. Scripts used for graph construction.

Supplementary File S4. Table S1. Species information considering chromosome number, genome size, and genomic datasets.

Supplementary File S5. Tables S2–S8. All information used for constructing graphs presented in this work. Includes summary information of Fig. 2 (Table S2), genome resources used in this study (Table S3), assembly statistics for Fig. 3A (Table S4), genome features of Fig. 3B (Tables S5 and S6), and BUSCO results for Fig. 4 and Supplementary Fig. S1 (Tables S7 and S8).

Supplementary File S6. Figure S1. BUSCO Eukaryota gene distribution in Medusozoa assemblies. Each column corresponds to a gene and each row an assembly. Information used for this graph is available in Supplementary File S5 Table S8.

Supplementary File S7. Dataset 4. Original metadata from NCBI.

Supplementary File S8. Dataset 5. Original results from AGAT and Galaxy server (BUSCO).

Supplementary File S9. Dataset 6. Figures in vectorial format.

giac036_GIGA-D-21-00404_Original_Submission

giac036_GIGA-D-21-00404_R1_Revision_1

giac036_Response_to_Reviewer_Comments_Original_Submission

giac036_Reviewer_1_Report_Original_Submission David A. Gold -- 1/9/2022 Reviewed

giac036_Reviewer_2_Report_Original_Submission Lucas LeclÃ¨re -- 1/13/2022 Reviewed

giac036_Reviewer_3_Report_Original_Submission Sheila Kitchen -- 1/17/2022 Reviewed

giac036_Supplemental_Files

## Abbreviations

BUSCO: Benchmarking Universal Single-Copy Orthologs; CDS: coding sequence; HTS: high-throughput sequencing; kb: kilobase pairs; Mb: megabase pairs; NCBI: National Center for Biotechnology Information; rRNA: ribosomal RNA; SRA: Sequence Read Archive; tRNA: transfer RNA.

## Competing Interests

The authors declare that they have no competing interests.

## Funding

This work was supported by Coordenação de Aperfeiçoamento de Pessoal de Nível Superior 88882.377420/2019-01 to M.D.S., Fundação de Amparo à Pesquisa do Estado de São Paulo FAPESP 2016/04560-9 to M.M.M., National Science Foundation 1935672 to J.F.R., and Fundação de Amparo à Pesquisa do Estado de São Paulo FAPESP 2015/20139-9 to S.C.S.A.

## Authors' Contributions

M.D.S. collected the information, ran the analysis, conceived the study, and drafted the manuscript; M.M.M. collected the information, conceived the study, and drafted and reviewed the manuscript; J.F.R. drafted and reviewed the manuscript; S.C.S.A. conceived the study and drafted and reviewed the manuscript. All authors gave final approval for publication.
