# **Correction to:** The state of Medusozoa genomics: current evidence and future challenges

**DOI:** 10.1093/gigascience/giac115

**Published:** 2022-12-12

This is a correction to: Mylena D Santander, Maximiliano M Maronna, Joseph F Ryan, Sónia C S Andrade, The state of Medusozoa genomics: current evidence and future challenges, GigaScience, Volume 11, 2022, giac036, https://doi.org/10.1093/gigascience/giac036

In the originally published version of this manuscript, caption to Figure 1 was incorrect. The word “*Carybdea” should be replaced with “Copula.”*

The phrase “Credits to Alvaro E. Migotto (A, B, E), Marta Chiodin (C), Joseph Ryan (F, G), and Cheryl Ames Lewis (D)” should be replated with “Credits to Alvaro E. Migotto (A, B, E, D), Marta Chiodin (C) and Joseph F. Ryan (F, G).”

The following text should be added at the end of the first paragraph in the “Methods” section:

“Assemblies identified as “preliminary” were not counted (Fig 2 and Main Text) or reanalyzed, but were detailed in Table 1 and Supplementary File S1.”

A corrected version “Supplementary File S1. Dataset 1. Genome report sheet” was uploaded.

These errors have been corrected

