# Supplementary material for: The state of Medusozoa genomics: current evidence and future challenges
Source: Gigascience. 2022 May 17;11:giac036. doi: 10.1093/gigascience/giac036 (PMC9112765; doi:10.1093/gigascience/giac036)
Supplement: giac036_GIGA-D-21-00404_Original_Submission [file giac036_giga-d-21-00404_original_submission.pdf]

GigaScience

The state of Medusozoa genomics: past evidence and future challenges

--Manuscript Draft--

**Manuscript Number:**	GIGA-D-21-00404
**Full Title:**	The state of Medusozoa genomics: past evidence and future challenges
**Article Type:**	Review
**Funding Information:**	Coordenação de Aperfeiçoamento de Pessoal de Nível Superior (88882.377420/2019-01) — Miss Mylena Daiana Santander<br>Fundação de Amparo à Pesquisa do Estado de São Paulo (FAPESP 2016/04560-9) — Mr. Maximiliano Manuel Maronna<br>Fundação de Amparo à Pesquisa do Estado de São Paulo (FAPESP 2015/20139-) — Miss Sónia Cristina da Silva Andrade
**Abstract:**	Medusozoa is a widely distributed ancient lineage that harbors one-third of Cnidaria diversity divided into four classes. This clade is characterized by the succession of stages and modes of reproduction during metagenic lifecycles, and includes some of the most plastic body plans and life cycles among animals. The characterization of traditional genomic features, such as chromosome numbers and genome sizes, was rather overlooked in Medusozoa and many evolutionary questions still remain unanswered. Modern genomic DNA sequencing in this group started in 2010 with the publishing of the Hydra vulgaris genome has experienced an exponential increase in the past three years. Therefore, an update of the state of Medusozoa genomics is warranted. We reviewed different sources of evidence, including cytogenetic records and high-throughput sequencing (HTS) projects. We focused on four main topics that would be relevant for the broad Cnidaria research community: 1) taxonomic coverage of genomic information; 2) continuity, quality and completeness of HTS datasets; 3) overview of the Medusozoa specific research questions approached with genomics; and 4) the accessibility of data and metadata. We highlight a lack of standardization in genomic projects and their reports, and reinforce a series of recommendations to enhance future collaborative research.
**Corresponding Author:**	Mylena Daiana Santander, Bachelor<br>Universidade de Sao Paulo Instituto de Biociencias<br>São Paulo, SP BRAZIL
**Corresponding Author Secondary Information:**	
**Corresponding Author's Institution:**	Universidade de Sao Paulo Instituto de Biociencias
**Corresponding Author's Secondary Institution:**	
**First Author:**	Mylena Daiana Santander, Bachelor
**First Author Secondary Information:**	
**Order of Authors:**	Mylena Daiana Santander, Bachelor<br>Maximiliano Manuel Maronna, PhD<br>Joseph F Ryan, PhD<br>Sónia Cristina da Silva Andrade, PhD
**Order of Authors Secondary Information:**	
**Additional Information:**	
**Question**	**Response**
Are you submitting this manuscript to a special series or article collection?	No

Powered by Editorial Manager® and ProduXion Manager® from Aries Systems Corporation

**Experimental design and statistics**<br><br>Full details of the experimental design and statistical methods used should be given in the Methods section, as detailed in our Minimum Standards Reporting Checklist. Information essential to interpreting the data presented should be made available in the figure legends.<br><br>Have you included all the information requested in your manuscript?	Yes
**Resources**<br><br>A description of all resources used, including antibodies, cell lines, animals and software tools, with enough information to allow them to be uniquely identified, should be included in the Methods section. Authors are strongly encouraged to cite Research Resource Identifiers (RRIDs) for antibodies, model organisms and tools, where possible.<br><br>Have you included the information requested as detailed in our Minimum Standards Reporting Checklist?	Yes
**Availability of data and materials**<br><br>All datasets and code on which the conclusions of the paper rely must be either included in your submission or deposited in publicly available repositories (where available and ethically appropriate), referencing such data using a unique identifier in the references and in the "Availability of Data and Materials" section of your manuscript.<br><br>Have you have met the above requirement as detailed in our Minimum Standards Reporting Checklist?	Yes

Manuscript Click here to access/download;Manuscript;Main_Text.docx

The state of Medusozoa genomics: past evidence and future challenges

Santander, Mylena D. 1 ORCID 0000-0001-6750-4180

Maronna, Maximiliano M. 2 ORCID 0000-0002-2590-639X

Ryan, Joseph F. 3,4 ORCID 0000-0001-5478-0522

Andrade, Sónia CS. 1 ORCID 0000-0002-1302-5261

Affiliation

1. Departamento de Genética e Biologia Evolutiva, Instituto de Biociências, Universidade São Paulo, 277 Rua do Matão, Cidade Universitária, São Paulo, Brazil. ZIP CODE 05508-090.
2. Departamento de Zoologia, Instituto de Biociências, Universidade de São Paulo, São Paulo, 101 Rua do Matão Cidade Universitária, São Paulo, Brazil. ZIP CODE 05508-090.
3. Whitney Laboratory for Marine Bioscience, University of Florida, 9505 Ocean Shore Blvd, St. Augustine, Florida, 32080, USA
4. Department of Biology, University of Florida, 220 Bartram Hall, Gainesville, FL, 32611, USA

Corresponding authors: MDS mylena.santander@gmail.com, MMM maxmaronna@gmail.com

Abstract

Medusozoa is a widely distributed ancient lineage that harbors one-third of Cnidaria diversity divided into four classes. This clade is characterized by the succession of stages and modes of reproduction during metagenic lifecycles, and includes some of the most plastic body plans and life cycles among animals. The characterization of traditional genomic features, such as chromosome numbers and genome sizes, was rather overlooked in Medusozoa and many evolutionary questions still remain unanswered. Modern genomic DNA sequencing in this group started in 2010 with the publishing of the *Hydra vulgaris* genome has experienced an exponential increase in the past three years. Therefore, an update of the state of Medusozoa genomics is warranted. We reviewed different sources of evidence, including cytogenetic records and high-throughput sequencing (HTS) projects. We focused on four main topics that would be relevant for the broad Cnidaria research community: 1) taxonomic coverage of genomic information; 2) continuity, quality and completeness of HTS datasets; 3) overview of the Medusozoa specific research questions approached with genomics; and 4) the accessibility of data and metadata. We highlight a lack of standardization in genomic projects and their reports, and reinforce a series of recommendations to enhance future collaborative research.

Keywords

Annotation, completeness, assembly, genome size, chromosome number, collaborative genomics

Background

Medusozoa subphylum includes nearly 4,055 species of invertebrates distributed in the classes Hydrozoa, Cubozoa, Staurozoa and Scyphozoa [1], which are found at all latitudes in almost all aquatic environments, from freshwater to marine, and from shallow to deep waters. Medusozoa species, together with the other cnidarians classes (i.e. Anthozoa and Endocnidozoa), harbor some of the most plastic life cycles and diverse body plans among animals [2], and represent one of its early diverging groups, with all major cnidarian lineages already present 500 million years ago [3].

The Medusozoa clade is characterized by different evolutionary novelties, such as the presence of linear mitochondria and the adult pelagic stage, also known as medusa or jellyfish [4–6]. Most medusozoan life-cycles are characterized by the succession of different stages, including a larval, benthic asexually reproducing polyp stage, and a sexually reproducing jellyfish stage [6,7]. This ancestral metagenic life-cycle pattern is highly plastic and in some groups has been extensively modified or even lost. For example, several lineages have lost the pelagic medusae or reduced it to a reproductive structure, or acquired colonial lifestyles during the benthic phase [8–10]. Other novel traits have emerged in Medusozoa such as complex body patterns, neuromuscular systems and sensory organs [11].

The history of Medusozoa genomics started with pioneer cytogenetics reports (e.g., [12,13]) and was followed later by genome size estimations [14,15]. Over the past 20 years, technological advances and cost reduction of genome-scale sequencing platforms have led

to a steady increase in both number and diversity of sequenced genomes and transcriptomes [16,17]. Medusozoa is not an exception, as numerous genomic resources have become available for model and non-model species, especially in the last 3 years. This advance has enabled the study of the genetic basis of many Medusozoa novel traits (e.g. [18–22]. Previous reviews about cnidaria genomics have focused on the small number of species with sequenced genomes available at the time [11,23,24], on individual cnidarian lineages (i.e. Myxozoa; [25]), or on specific topics such as toxins or evolution of novel traits [11,26]. Given the increasing amount of genomic information available, an update of the state of Medusozoa genomics is warranted.

Here, we provide a comprehensive review of the major advances in Medusozoa genomics over the past century. In order to shed light in the understanding of the genomic evolution of the group from high throughput sequencing (HTS) datasets, we report the main trends on the number and quality of available genome projects, taking into account basic information of sequencing datasets, genome assemblies, genome annotations, and accessibility of associated data and metadata.

Main text

1. Methods

We surveyed literature and databases for cytogenetic reports and genome size estimations. Our main source was NCBI Genome (Assembly, Genomes, Nucleotide, Taxonomy and SRA; [27]). For the information not present in NCBI, published articles were checked for proper information collection, as well as personal repositories mentioned in the associated articles. Due to recent updates in taxonomic statuses, we modified the attribution of karyotypes, genome sizes and assemblies of several species (see main text and Supplementary Materials).

Because there have been subtle variations in metrics and statistics between most genome reports, we recalculated some statistics, allowing us to make meaningful comparisons. Briefly, we have generated the following: i) assembly statistics statswrapper.sh script from BBmap (v38.73; RRID:SCR_016965; [28]); ii) gene statistics from the original annotation files with AGAT (v0.6.0; [29]) and assessment of completeness of all assemblies using BUSCO (v5.0.0+galaxy0; RRID:SCR_015008; [30]) in genome mode and Metaeuk software, using two Single Orthologs Databases (eukaryota_odb10, number of genes=255, number of species=70; metazoa_odb10, number of genes=954, number of species=65), available at the public Galaxy server [31,32].

Assembly quality was reported following the metric proposed by Earth Biogenome Project [33] (hereafter BGP-metric). This system avoids the use of ambiguous terminology for quality and uses a logarithmic scale where the first two numbers are the exponents of the N50 contig and scaffold (1: 0-99Kb; 2: 1-9.9Mb; 3: 10-99.9Mbp), and the third number corresponds to the level of chromosomal assembly (1: 90% DNA > assigned to chromosomes in silico; 2: chromosomal rearrangements validated by two data sources; 3: >80% DNA assigned to intra-species maps and experimental validation of all breakpoints; see [33]).

All graphs were generated using Python v.3 with ETE Toolkit v.3 [34], Matplotlib v3.3.1 [35] and Seaborn v.0.11 [36]. The tree of figures 1 and 3 represent a simplified phylogenetic hypothesis obtained by combining phylogenies from previous studies (Scyphozoa [37], Medusozoa [5], Hydrozoa [38,39]), taking into account clades with high congruence and support values. Although the different phylogenetic hypotheses were mostly congruent, no single study nor molecular dataset comprised all the terminals discussed here. To compile all genomic information and HTS metadata referenced in this review, we created a report model, based on previous works and public databases such as NCBI (Supplementary file S1; [29,40,41]). All collected data was updated until May 1st 2021.

2. Genomic projects: whos and hows of Medusozoa

Chromosome numbers are known for 34 hydrozoan species and 5 scyphozoan, including 3 lineages of the *Aurelia aurita* sp. complex species ([12,13,21,42–50]; Supplementary file S2). Older chromosome descriptions for 25 species do not include information about chromosome morphology and often lack photographic records or schematic representations [12,13,42–46].

Genome size, a fundamental feature in genome sequencing project, has been experimentally estimated by Flow Cytometry or Feulgen Densitometry techniques, for 24 medusozoan species (Scyphozoa: 7spp.; Cubozoa: 1spp.; Hydrozoa: 16 spp.; Supplementary file S2). Genome sizes are highly variable ranging from 254 Megabases (Mbp) to 3,481.68 Mbp in *Sanderia malayensis* (Scyphozoa) and in *Agalma elegans* (Hydrozoa), respectively [15]. Moreover, an additional 12 genome size estimates are available when considering k-mer-based computational assessments, increasing the number of species with genome size information to 30, and including two cubozoans (913-2,673Mbp) and one staurozoan (230 Mbp) (Supplementary file S1; Supplementary file S2). These estimates are considered less accurate, especially for genomes with high heterozygosity, high repetitive content and large genome size [51]. In fact, kmer based and experimental estimations from the same species differed by 13-33%.

A total of 34 HTS projects were identified. Of these, 32 had sequencing reads accessible through the NCBI-SRA database but not all of them were associated with a genome assembly (Table 1; Supplementary file S1). The taxonomic coverage of the assemblies encompassed 7 of the 13 Medusozoa orders, and represented at least one species per class (Figure 1): 28 assemblies were accessible for 21 species, representing 0.5 % of Medusozoa (Figure 1; Table 1; Supplementary file S1). Of these 21 species, 12 were Scyphozoa, 4 were Hydrozoa, 4 were Cubozoa, and one was Staurozoa. Scyphozoa had the highest number of sequenced families (4 of 22), of which Pelagiidae contained the highest number of sequenced species so far (5 spp.), followed by Ulmaridae, Rhizostomatidae and Cassiopeiidae with 2 spp. each (Figure 1), all belonging to subclass Discomedusae (none from Coronamedusae). The

remaining assemblies represent three of the eight Cubozoa families and three of 135 Hydrozoan families (Figure 1). In addition to the small fraction of family representation in the hydrozoan genomes, the underrepresentation of Leptothecata is particularly unfavorable as it harbors more than half of Medusozoa species (2,059 sp; [1]).

------------TABLE 1 SHOULD BE LOCATED HERE------------

Much of the assembly effort is biased towards a small number of species. For example, three species of Hydrozoa and Scyphozoa presented two assemblies each, of which *Hydra viridissima* and *Rhopilema esculentum* were sequenced twice independently, meanwhile *Chrysoaora quinquecirrha* presents two versions of the same assembly. Moreover, three assemblies were available for two different strains of *Hydra vulgaris* (former *Hydra magnipapillata*), one of them published as an update of the reference genome called Hydra 2.0. In *Aurelia,* the genomes of three different lineages were sequenced and assembled: Baltic sea, Roscoff and *Aurelia* sp1. strains [19,20]. Based on a recent taxonomic update of this genus [52], locality and genetic information described in the original articles [19,20], we decided to refer to these genomic datasets as: Baltic sea strain = *Aurelia aurita;* Roscoff strain and *Aurelia* sp1. strains = *Aurelia coerulea*.

Most of the assemblies were deposited in NCBI Assembly database, one was only found in a journal-specific database (i.e. GigaDB [53]), one assembly was only in a personal repository (Google Drive) and one in the National Human Genome Research Institute site [54]. Some assemblies were additionally deposited in Institute-centered repositories such as OIST Marine Genomics Unit [55], the Marine Invertebrate Models Database (MARIMBA, [56]). A significant portion of the publicly available assemblies (total of 8, ~30%) are not yet associated with a formal publication and belong to the IRIDIAN GENOMES project [57]. The most frequent sequencing technology was Illumina (26 assemblies, ~93%), but leaving aside unpublished ones, most works include a combination of different sequencing techniques, library sizes and

platforms (i.e Sanger, 454, Illumina, long reads, linked-reads and Hi-C sequencing; Supplementary file S1).

Almost all medusozoan genome assemblies were at draft contig or scaffold level, with one exception, *Rhopilema esculentum*, where chromosome-level scale assembly was reported [58]. The total length, contig and scaffold number, N50, and GC% varied across species and classes (Figure 2A; references in Supplementary file S3). The assembly continuity and quality was higher in Scyphozoa than in the other classes, as observed by the distribution of contig and scaffold N50 (Figure 2A) and the BGP-metric for assembly quality (Figure 2A). In general, they are fragmented (75%), and have contig N50 of less than 40 Kbp (Figure 2A; BGP-metric values of 0.0.0, 0.1.0 and 0.2.0). Staurozoa, Cubozoa and Scyphozoa assemblies have similar percentages of base composition, around 35% to 43% GC. Consistent with previous reports [59], Hydrozoa genomes have a higher dispersion of GC%, with the GC values of five assemblies below 35%.

In relation to gene content (Figure 2B), 17 genomes were annotated using at least one source of information (Supplementary file S1) and their total number of genes or total number of protein-coding genes were reported. Further description of coding information was variable among works and as more detailed information was considered, the number of genomes with reported information decreased. Annotation tracks and gene models were available for only 11 of the 17 datasets. Recalculations of gene features together with the information recovered from original articles, allowed us to analyze the distribution of 5 different features in 15 genomes of Scyphozoa, Hydrozoa and Cubozoa (Figure 2B; Box): Number of genes (n=15), Mean exons per cds (n=10), Mean gene length (n=11), Mean exon length (n=11), Mean intron length (n=12). For three species, *Cassiopea xamachana* (Scyphozoa; 31,459), *Alatina alata* (Cubozoa; 66,156) and *Calvadosia cruxmelitensis* (Staurozoa; 26,258), the available information was restricted to the number of predicted genes.

The determination of repetitive DNA has been an integral step before gene annotation in most genomic projects. Frequently, repeat diversity was not properly reported and the degree of detail also varied between articles: e.g. some published works only referred to the most abundant class of repetitive DNA, meanwhile others described only results at class or family level. Repetitive libraries —consensus sequences representing repeat families— were not properly saved in repositories with the exception of two independent articles, and RepeatMasker results were reported in 4 articles (one reporting only classified repeats). Total repetitive length of 12 species for which coding information was also available is presented in Figure 2B and discussed in Box.

The degree of completeness of these datasets also varied substantially, as estimated by BUSCO (metazoa_odb10 and eukaryota_odb10; Figure 3). While all Eukaryota genes were present in at least one assembly (Supplementary file S3, Supplementary file S4), the level of absence and fragmentation of Metazoa genes was higher (Figure 3. Supplementary file S3). Seven Metazoa genes were absent in all assemblies and 17 were absent in more than 20% of them (Figure 3, indicated in red). Some Metazoa BUSCO genes were absent in lineages with the higher number of assemblies, such as Scyphozoa and Hydrozoa (Figure 3. indicated in yellow rectangles; Supplementary file S3). This condition was suggested by [20], after detecting the absence of 14 genes in 5 species (version metazoa_o9db), 3 of which coincided with the genes detected as absent here (Orthodb IDs: 460044at33208, 601886at33208, 114954at33208), one of which (445034at33208) that has a patchy distribution in Medusozoa and 9 of which were removed in later versions of the database (Figure 3 in bold).

Moreover, 27 genes were simultaneously recovered as undetectable or fragmented in more than 80% of the assemblies (Supplementary file S3). Based on BUSCO completeness assessment with metazoa_o10db, 13 assemblies present 90-95% of genes (fragmented+complete), while only one assembly includes over 90% of complete genes; the remaining 15 assemblies present between 57-87% of genes (complete+fragmented) or 16-

77% complete genes. While the Metazoa database might include genes that are absent, fragmented, or have non-conventional features in all medusozoa species, the utility of the Eukaryota database in the completeness assessment is limited by its low number of genes. Until more specific databases are developed, the combination of both BUSCO databases should be used taking into account their limitations.

3. The state of Medusozoa genomics: inner and derived knowledge

The first glimpse of the Medusozoa genomic organization was obtained by cytogenetic studies [12,13,21,42–50], but in contrast to other animals, the available information is still sparse. Many cytogenetic questions essential to the understanding of genome evolution are unanswered in Medusozoa, either at species or population scale, including the distribution of the chromosome number (2n), fundamental number of chromosome arms (FN), genome size, ploidy level, heterochromatin contente. These are questions that have gained renewed interest since the arrival of the genomic era.

Regarding the phylogenetic distribution of the chromosome number, no inferences can yet be made on the sparse available information, apart from the presence of some chromosome variation throughout Medusozoa. A special case was reported in *Hydra* where, according to recent descriptions, many species shared a 2n=30 karyotype with metacentric or submetacentric chromosomes ([50]; Supplementary file S2). This suggests that the 2n=30 karyotype could be widely distributed in the genus and even in other Hydrozoa groups, since it was also described for one species of Hydrocorynidae, Hydractiniidae, Campanulariidae, Bougainvilliidae, and Clytiidae, and 3 Eirenidae (Supplementary file S2; references therein). Interestingly, in Anthozoa, a few sea anemones and several scleractinian corals have karyotypes between 2n=28 and 2n=30 [60–62]. Nevertheless, a higher sampling effort should be conducted in order to test the extent of this apparent karyotype stability.

Scyphozoa genomes tend to be restricted to smaller sizes (~250 to ~700 Mbp) than those of Hydrozoa, which encompass a larger range (~380 to ~3,500 Mbp) (Figure 1; Supplementary file S2, references therein), but due to the scarcity of estimations that represent around 1% of the subphylum, these ranges should be considered preliminary. The evolution of genome size is a long-standing question that is included in the so-called C-value Enigma [40]. The latter cover several widely discussed frameworks and hypotheses that try to explain the causes and consequences of genome size variation and that have found support in different organisms (reviewed in [63]. The molecular basis of these variations in Medusozoa have only been studied in detail for *Hydra* [64] and for *S. malayensis* [65]; their trends have been related to repetitive DNA and gene length respectively (Box). Meanwhile, the ecological and historical factors underlying genome size diversity and its extent in Medusozoa, are topics that remain to be elucidated.

Box. Genome content

Gene content and length: it is straightforward to imagine that the evolution of these two characteristics have potential impacts in macroevolution of organisms. The distribution of gene number in Medusozoa (Figure 2B) ranged from 17,219 in the Scyphozoan *Rhopilema esculentum* [58] to 66,156 in the Cubozoan *Alatina alata* [22], but most species of all classes have gene counts near the median (26,258), which is higher than the range (18,943 ± 451.82) described for animals [40]. The upper limit described in the highly fragmented *A. alata* genome deviates from the observed in *Morbakka virulenta (*24,278 genes), the only other sequenced Cubomedusae [66]. Species with varying genome sizes of Hydrozoa, Scyphozoa and *M. virulenta* (Cubozoa) had similar mean CDS lengths (1,414, 1,214, 1,387 base pairs), mean numbers of exons per gene (5, 6, 5.4), mean exon lengths (306, 293, 432

bp), but had different gene lengths (9,530, 7,855 and 21,444 bp respectively) due to the presence of longer introns in Hydrozoa and Cubozoa when compared to Scyphozoa (Hydrozoa: 1,600; Cubozoa: 3,705 vs. 1,146 bp in Scyphozoa). This is best exemplified in the genome of the scyphozoan *S. malayensis*, which has the smallest cnidarian genome reported to date [65], and has also the smallest introns of any sequenced medusozoan genome (Figure 2B. yellow arrowhead). Nevertheless, these ranges are rough estimates and sometimes heterogeneous, e.g. resulting from different filtering parameters, and their implications should be tested as new assemblies and annotations become available.

Repetitive content: repetitive DNA represents a significant part of eukaryotic genomes and is highly diverse, composed by different kinds of transposable elements (TEs), tandem repeats and multigene families (e.g. rRNA and tRNA). Many of these sequences, especially TEs and satellite DNA, were initially considered as an expendable sector of the genome, although their impact on genomic evolution has since been recognized (reviewed in [67]). For example, fusion between TEs and host genes have occurred multiple times in vertebrates and have contributed to the evolution of novel features [68]. Likewise, TEs and other repetitive DNA have been associated with genomic rearrangements and changes in DNA content (e.g. [64,67]). The *Hydra* genus, which has been more extensively studied from this point of view, has experienced a rapid genomic evolutionary rate and presents a 3-fold genome size increase resulting from the amplification of a single LINE family [64]. Moreover, *Hydra* genomes include an over-representation of transposase-related domains [69]. It is interesting to note that many of the Medusozoa species studied so far have relatively small genomes but unusually high proportions of repetitive DNA [20,65,66,70]. Nevertheless, the lack of standardization in the description of its diversity, and the discrepancy in the degree of detail in which these have been described, limits the potential to make inferences. Repetitive DNA is a complex study subject, limited by assembly continuity and annotation

effort, but restricting genomic studies to the “functional” part of the genome (sensu [71]) may lead us to a narrowed view of the Medusozoa genome evolution.

Modern Medusozoa genomics formally started with the sequencing and publication of *Hydra vulgaris* genome [72] that in cnidaria was only preceded by *Nematostella vectensis* [60,72]. *Hydra vulgaris* is one of the earliest models in biology, mainly used for the study of development, regeneration, and more recently, of aging (reviewed in [73,74]). The study of these two early genomes was fundamental for the reconstruction of a more complex ancient eumetazoan genome than first suggested by the comparison of vertebrates and insects [16,23,60,72]. Nevertheless, unlike most other medusozoan species, *Hydra* lives in freshwater, lacks a medusa and has a genome that has experienced a very rapid rate of evolution [21]. It therefore is not the ideal species for reconstructing historical nodes on the Medusozoa tree of life. As such, more recent medusozoa genomes have led to important updates in our understanding of Medusozoa-relevant research topics, including phylogenetic reconstructions, the genetic basis of the medusae, the evolution of symbiosis, toxin characterization, Homeobox gene evolution, to name a few examples (Table 1). Nevertheless, Medusozoa genomes include thousands of single-copy genes and repetitive elements; however, only a very limited number of them have been analyzed in detail.

The determination of lineage specific genes and increases and decreases of gene content is one of the recurrent questions found in Medusozoa genomic studies (e.g. [20,21], but see [75,76]), and it has been carried on using different methodologies and sets of species. It has been estimated the most elevated rates of loss in Cnidaria in the hydrozoan branch leading to *Clytia hemisphaerica* and *Hydra* [21,72], followed by slightly lower rates of gene loss in Scyphozoa and substantially lower rates in Anthozoa [19]. Gene families that have experienced expansion and contraction have been studied in relation to complex life cycle patterns [19,21], simplification of the body plan [69,72], the evolution of symbiosis [69], among

others (table 1). Expression patterns of identified taxonomically restricted medusozoan genes have been mainly studied in the context of life cycle stages (e.g. [20,21]).

On the other hand, synteny was also analyzed several times, including species of Hydrozoa, Cubozoa and Scyphozoa, and were carried on at different scales depending on assembly continuity (i.e. microsynteny and macrosynteny), and often comparing the focus species to species from sister clade Anthozoa [19–21,62,72]. High synteny conservation was found within Anthozoa (*N. vectensis* vs. *Scolanthus callimorphus* [60–62]) and within Hydrozoa (*H. vulgaris* vs. *C. hemispherica;* [21]). Meanwhile, conservation of synteny at a lesser degree was also observed between Anthozoa and Scyphozoa (*N. vectensis* vs. *R. esculentum*; *N. vectensis* vs. *Aurelia* strains; [19,20,62]) and only a few shared syntenic blocks between Hydozoa and Anthozoa (*H. vulgaris* vs. *N. vectensis;* [21,62,72]), Hydrozoa and Scyphozoa (*H. vulgaris* vs. *Aurelia aurita;* [19]) and Scyphozoa and Cubozoa (*A. aurita vs. M. virulenta*; [20]). It is particularly interesting to note that *H. vulgaris, N. vectensis* and *S. callimorphus* present 2n=30, but shared fewer syntenic blocks than either of the two anthozoans with *R. esculentum*, which has a different karyotype (2n=22) [62] (non peer-reviewed). These results suggest that there is evidence for the conservation of an ancient genome architecture in Anthozoa and Scyphozoa, but less conservation in Hydrozoa and Cubozoa, coincident with a more rapid rate of genome reorganization in the last two classes [21,62].

4. Prospects on genomic data and general resources

The increasing amount of genomic information available for diverse organisms has enabled statistical inferences of trends in eukaryotic genomic evolution. Examples of such studies are available at small and large phylogenetic scales and have enabled evolutionary analyses of the distribution of gene numbers, gene features (e.g. intron size), and repetitive content (e.g. [40]). Nevertheless, the power of eukaryotic genomic comparative analyses is

hindered by a lack of data and metadata standardization [40,77], which is especially evident in Medusozoa.

We analyzed hundreds of fields including genetic information and metadata (methods, metrics and registry codes; table Supplementary file S1), of which no dataset presents most of them, whatever the area or section (e.g., processing area, section trimming). This could be a future problem because reusing of previously published datasets is becoming routine, and tracking of information (BioProjects, Biosamples, methodologies, filtering parameters, etc.) would be misleading [77,78].

The submission of raw sequencing data and fundamental metadata to the NCBI-SRA or EMBL-ENA remains a vital step in ensuring the usability and transparency of genome data [79,80]. Also, project centric repositories serve to store assemblies and associated datasets, and enable comparative studies. Nevertheless, their use should not lead to the abandonment of general databases, because it can result in the loss of fundamental metadata associated with a genomic project and has the potential to aggravate the discovery and re-usability problem [81]. For example, the assembly with the highest continuity as estimated by the BGP-metric, corresponding to *R. esculentum* [58], is only found in a journal specific database and lacks an stable identifier (e.g. NCBI accession). Moreover, even a simple deposit in a public database would call our attention to potential issues such as contamination (e.g. see online SRA runs SRR13700068 and SRR13036460).

About lack of past data and current limitations, we should learn from decades-old references of cytogenetic studies: because some of them do not provide complete material and methods (e.g., pretreatment, references, designs and photographs; general metadata as locality, taxonomic identification) and their results therefore should be considered carefully in a comparative framework (e.g., [16,82,83]). For example, we identified at least three independent projects that adopted different criteria for gene model filtering, and other three articles with slightly different criteria for repeat library filtering (Supplementary file S1). As

additional proof of this idea, this review presents a reanalysis on genome completeness by BUSCO, that was reran to ensure that comparisons were made between identically run analyses and database versions, which were frequently unspecified in the associated articles.

There is a growing number of community-driven guidelines, standards, databases and resources based on the Findable, Accessible, Interoperable and Reusable principles (FAIR principles) for digital research outputs [81]. Furthermore, global initiatives of large-scale genome sequencing included in Earth Biogenome Project have adopted a set of standardized protocols for the different stages of the genome projects, such as specimen collection, DNA extraction, sequencing, assembly and annotation methods, and reporting, in order to generate datasets that could “be useful to the broadest possible scientific community” [33]. Standards should be also implemented by independent research groups publishing genomes. The main goal of standardization is to promote evaluation, discovery, and reuse of genomic information, providing long term benefits for science.

The following are suggestions to enhance genome projects and outcomes, and to promote open and collaborative research.

1. Deposit all data and metadata in public specialized databases (e.g., NCBI), at least once associated articles are accepted for publication. Detail most metadata as possible, including those not considered as priority for the aforementioned project.

2. When possible, use a single standardized genome report format, based on previous Medusozoa projects (e.g. Supplementary file S1 presented here). This will help to recognize and select proper metadata options for new ones and will enable comparisons between studies; Alternatively, use specialized tools that standardize reports for multiple samples and datasets (e.g. [41,84,85]).

3. Deposit output results that were fundamental in any of the steps (e.g. gene models, repetitive libraries and annotation tracks).

4. Inform as much as possible if a dataset was edited (e.g., decontamination; gene and repetitive sequence filtering criteria).

5. Use and clearly identify software, database versions and references in all instances (e.g., RRID, BUSCO version and repetitive database version).

6. Deposit command lines and scripts used to handle data (from reads to full annotation).

Conclusions

The pace of genomic development in Medusozoa is far more rapid than more traditional disciplines such as cytogenetics, where gaps still remain. As the effect of chromosome structural variants in evolution is increasingly tested and recognized, it is expected that these disciplines will gain a revived interest as has been seen in other animal groups [86]. In spite of the great advances in Medusozoa genomics, we found a general lack of standardization in methodologies and genome reports across independent sequencing projects. Efforts to incorporate standards would benefit future studies and could promote the identification of hitherto undiscovered evolutionary patterns.

It is safe to anticipate that standardization will become increasingly easier as chromosome-level assemblies become more commonplace and as new integrated workflows of data reporting are developed (e.g. [87]). It will be possible to perform standardized annotation and analyses in order to identify patterns in medusozoa genome evolution. Conversations about how best to promote such efforts and best practices for medusozoan genome efforts will help move the field forward. There are several potential platforms for gathering community input (e.g., Cnidofest [88], “coelenterate” biology [89], Tutzing workshop [90]). Such conversations could lead to new standards and potentially a powerful cnidarian genomics database. This latter goal would be most effective if accompanied by a strong alliance that spans the growing cnidarian genomics community.

Data availability

All collected information and outputs supporting new results are made available through the supplementary files S1-S9 and in figshare [91] (public release if accepted). All genomic resources from previous articles and proyects are publicly available and are their sources are referenced in Supplementary file S3 Table S3.

Competing interests

The authors declare that they have no competing interests

Author's contributions

MDS collected the information, ran the analysis, conceived the study and drafted the manuscript; MMM collected the information, conceived the study, drafted and reviewed the manuscript; JR drafted and reviewed the manuscript. SCSA conceived the study, drafted and reviewed the manuscript. All authors gave final approval for publication.

Funding

This work was supported by Coordenação de Aperfeiçoamento de Pessoal de Nível Superior 88882.377420/2019-01 to MDS, Fundação de Amparo à Pesquisa do Estado de São Paulo FAPESP 2016/04560-9 to MMM, FAPESP 2015/20139-9 to SCSA.

Acknowledgments

We thank Analia Jaque for the jellyfish drawings of Figure 1 and Juan Martin Ferro for valuable comments on this review.

References

1. World Register of Marine Species. Cnidaria. http://www.marinespecies.org/aphia.php?p=taxdetails&id=126. Accessed 24 Nov 2021.

2. Bosch TCG, Adamska M, Augustin R, Domazet-Loso T, Foret S, Fraune S, et al.. How do environmental factors influence life cycles and development? An experimental framework for early-diverging metazoans. *BioEssays*. 2014; doi: 10.1002/bies.201400065.

3. Cartwright P, Collins AG. Fossils and phylogenies: integrating multiple lines of evidence to investigate the origin of early major metazoan lineages. *Integr Comp Biol*. 2007; doi: 10/bzhzc2.

4. Bridge D, Cunningham CW, Schierwater B, DeSalle R, Buss LW. Class-level relationships in the phylum Cnidaria: evidence from mitochondrial genome structure. *Proc Natl Acad Sci U S A*. 1992; doi: 10/dxgw77.

5. Kayal E, Bentlage B, Sabrina Pankey M, Ohdera AH, Medina M, Plachetzki DC, et al.. Phylogenomics provides a robust topology of the major cnidarian lineages and insights on the origins of key organismal traits. *BMC Evol Biol*. 2018; doi: 10.1186/s12862-018-1142-0.

6. Marques AC, Collins AG. Cladistic analysis of Medusozoa and cnidarian evolution. *Invertebr Biol*. 2004; doi: 10.1111/j.1744-7410.2004.tb00139.x.

7. Collins AG. Phylogeny of Medusozoa and the evolution of cnidarian life cycles. *J Evol Biol*. 2002; doi: 10.1046/j.1420-9101.2002.00403.x.

8. Boero F, Boero F Bouillon, J. Zoogeography and life cycle patterns of Mediterranean hydromedusae (Cnidaria). *Biol J Linn Soc*. 1993; doi: 10/cgvp43.

9. Da Silveira FL, Morandini AC. *Nausithoe aurea* n. sp.(Scyphozoa: Coronatae: Nausithoidae), a species with two pathways of reproduction after strobilation: sexual and asexual. *Contrib Zool*. 1997;66:235–46.

10. Straehler-Pohl I, Jarms G. Morphology and life cycle of *Carybdea morandinii*, sp. nov.(Cnidaria), a cubozoan with zooxanthellae and peculiar polyp anatomy. *Zootaxa*. 2011;2755:36–56.

11. Forêt S, Knack B, Houliston E, Momose T, Manuel M, Quéinnec E, et al.. New tricks with old genes: the genetic bases of novel cnidarian traits. *Trends Genet*. 2010; doi: 10/dd74gw.

12. Harvey EB. A review of the chromosome numbers in the Metazoa. Part I. *J Morphol*. 1916; doi: 10.1002/jmor.1050280102.

13. Makino S. An atlas of the chromosome numbers in animals. 2nd ed. Ames: The Iowa State College Press; 1951.

14. Goldberg RB, Crain WR, Ruderman JV, Moore GP, Buckley TR, Higgins RC, et al.. DNA sequence organization in the genomes of five marine invertebrates. *Chromosoma*. 1975; doi: 10.1007/BF00284817.

15. Adachi K, Miyake H, Kuramochi T, Mizusawa K, Okumura S. Genome size distribution in phylum Cnidaria. *Fish Sci*. 2017; doi: 10/ggbqnf.

16. Dunn CW, Ryan JF. The evolution of animal genomes. *Curr Opin Genet Dev*. 2015; doi: 10/gfkjbf.

17. Goodwin S, McPherson JD, McCombie WR. Coming of age: ten years of next-generation sequencing technologies. *Nat Rev Genet*. 2016; doi: 10.1038/nrg.2016.49.

18. Lewis Ames C, Ryan JF, Bely AE, Cartwright P, Collins AG. A new transcriptome and transcriptome profiling of adult and larval tissue in the box jellyfish *Alatina alata*: an emerging model for studying venom, vision and sex. *BMC Genomics*. 2016; doi: 10.1186/s12864-016-2944-3.

19. Gold DA, Katsuki T, Li Y, Yan X, Regulski M, Ibberson D, et al.. The genome of the jellyfish Aurelia and the evolution of animal complexity. *Nat Ecol Evol*. 2019; doi: 10/gfkwp4.

20. Khalturin K, Shinzato C, Khalturina M, Hamada M, Fujie M, Koyanagi R, et al.. Medusozoan genomes inform the evolution of the jellyfish body plan. *Nat Ecol Evol*. 2019; doi: 10/gfzg9m.

21. Leclère L, Horin C, Chevalier S, Lapébie P, Dru P, Peron S, et al.. The genome of the jellyfish *Clytia hemisphaerica* and the evolution of the cnidarian life-cycle. *Nat Ecol Evol*. 2019; doi: 10/gfwr3v.

22. Ohdera A, Ames CL, Dikow RB, Kayal E, Chiodin M, Busby B, et al.. Box, stalked, and upside-down? Draft genomes from diverse jellyfish (Cnidaria, Acraspeda) lineages: *Alatina*

alata (Cubozoa), *Calvadosia cruxmelitensis* (Staurozoa), and *Cassiopea xamachana* (Scyphozoa). *GigaScience*. 2019; doi: 10.1093/gigascience/giz069.

23. Steele RE, David CN, Technau U. A genomic view of 500 million years of cnidarian evolution. *Trends Genet TIG*. 2011; doi: 10/b53t8x.

24. Technau U, Schwaiger M. Recent advances in genomics and transcriptomics of cnidarians. *Mar Genomics*. 2015; doi: 10.1016/j.margen.2015.09.007.

25. Alama-Bermejo G, Holzer AS. Advances and Discoveries in Myxozoan Genomics. *Trends Parasitol*. 2021; doi: 10.1016/j.pt.2021.01.010.

26. D'Ambra I, Lauritano C. A Review of Toxins from Cnidaria. *Mar Drugs*. 2020;18:507.

27. NCBI Resource Coordinators N. Database resources of the National Center for Biotechnology Information. *Nucleic Acids Res*. 2015; doi: 10.1093/nar/gku1130.

28. Bushnell B. BBMap v38.73. https://sourceforge.net/projects/bbmap/. Accessed 25 May 2021.

29. Dainat J, Hereñú D, Pucholt P. NBISweden/AGAT: AGAT-v0.6.0. *Zenodo*. 2021; doi: 10.5281/zenodo.4637977.

30. Simão FA, Waterhouse RM, Ioannidis P, Kriventseva EV, Zdobnov EM. BUSCO: assessing genome assembly and annotation completeness with single-copy orthologs. *Bioinformatics*. 2015; doi: 10.1093/bioinformatics/btv351.

31. Afgan E, Baker D, Batut B, van den Beek M, Bouvier D, Čech M, et al.. The Galaxy platform for accessible, reproducible and collaborative biomedical analyses: 2018 update. *Nucleic Acids Res*. 2018; doi: 10.1093/nar/gky379.

32. Galaxy. https://usegalaxy.org/. Accessed 10 Aug 2021.

33. Lewin HA, Robinson GE, Kress WJ, Baker WJ, Coddington J, Crandall KA, et al.. Earth BioGenome Project: Sequencing life for the future of life. *Proc Natl Acad Sci*. 2018; doi:

10/gdh5vz.

34. Huerta-Cepas J, Serra F, Bork P. ETE 3: Reconstruction, analysis and visualization of phylogenomic data. *Mol Biol Evol*. 2016; doi: 10/gfzpph.

35. Caswell TA, Droettboom M, Lee A, Hunter J, Firing E, Andrade ES de, et al.. Matplotlib release v3.3.1. *Zenodo*. 2020; doi: 10.5281/zenodo.3984190.

36. Waskom ML. Seaborn: statistical data visualization. *J Open Source Softw*. 2021;6:3021.

37. Bayha KM, Dawson MN, Collins AG, Barbeitos MS, Haddock SHDD. Evolutionary relationships among scyphozoan jellyfish families based on complete taxon sampling and phylogenetic analyses of 18S and 28S ribosomal DNA. *Integr Comp Biol*. 2010; doi: 10.1093/icb/icq074.

38. Maronna MM, Miranda TP, Peña Cantero ÁL, Barbeitos MS, Marques AC. Towards a phylogenetic classification of Leptothecata (Cnidaria, Hydrozoa). *Sci Rep*. 2016; doi: 10/ggbrh4.

39. Mendoza- Becerril MA, Jaimes- Becerra AJ, Collins AG, Marques AC. Phylogeny and morphological evolution of the so- called bougainvilliids (Hydrozoa, Hydroidolina). *Zool Scr*. 2018; doi: 10/gd4ftz.

40. Elliott TA, Gregory TR. What's in a genome? The C-value enigma and the evolution of eukaryotic genome content. *Phil Trans R Soc B*. 2015; doi: 10/gfkjbq.

41. Wilbrandt J, Misof B, Niehuis O. COGNATE: comparative gene annotation characterizer. *BMC Genomics*. 2017; doi: 10/ggbrjp.

42. Tardent P. Coelenterata, Cnidaria. 1st ed. Jena/Stuttgart: Gustav Fischer; 1978.

43. Kubota S. Systematic study on a bivalve-inhabiting hydroid *Eucheilota intermedia* Kubota from central Japan. *J Fac Sci Hokkaido Univ Ser VI Zool*. 1985;24:122-143.

44. Kubota S. Taxonomic Study on *Hydrocoryne miurensis* (Hydrozoa). *Publ SETO Mar Biol*

Lab. 1988;33:1–18.

45. Kubota S. Second finding of *Stylactaria piscicola* (Komai, 1932) comb. nov. (Hydrozoa: Hydractiniidae) from off Atsumi Peninsula, Japan. *Publ Seto Mar Biol Lab*. 1991;35:11–5.

46. Kubota S. Chromosome number of a bivalve-inhabiting hydroid, *Eugymnanthea japonica* (Leptomedusae: Eirenidae) from Japan. *Publ SETO Mar Biol Lab*. 1992;35:383–6.

47. Ping Guo. The karyotype of *Rhopilema esculenta*. *J Fish China*. 1994;18:253–5.

48. Anokhin B, Kuznetsova V. Chromosome morphology and banding patterns in *Hydra oligactis* Pallas and *H. circumcincta* Schultze (Hydroidea, Hydrida). *Folia Biol (Krakow)*. 1999;47:91–6.

49. Anokhin B, Nokkala S. Characterization of C-heterochromatin in four species of Hydrozoa (Cnidaria) by sequence specific fluorochromes Chromomycin A_3 and DAPI. *Caryologia*. 2004; doi: 10.1080/00087114.2004.10589387.

50. Anokhin BA, Kuznetsova VG. FISH-based karyotyping of *Pelmatohydra oligactis* (Pallas, 1766), *Hydra oxycnida* Schulze, 1914, and *H. magnipapillata* Itô, 1947 (Cnidaria, Hydrozoa). *Comp Cytogenet*. 2018; doi: 10/gfst43.

51. Pflug JM, Holmes VR, Burrus C, Johnston JS, Maddison DR. Measuring Genome Sizes Using Read-Depth, k-mers, and Flow Cytometry: Methodological Comparisons in Beetles (Coleoptera). *G3 (Bethesda)*. 2020; doi: 10.1534/g3.120.401028.

52. Lawley JW, Gamero-Mora E, Maronna MM, Chiaverano LM, Stampar SN, Hopcroft RR, et al.. The importance of molecular characters when morphological variability hinders diagnosability: systematics of the moon jellyfish genus *Aurelia* (Cnidaria: Scyphozoa). *PeerJ*. 2021; doi: 10.7717/peerj.11954.

53. GigaDB. http://gigadb.org. Accessed 1 Apr 2021.

54. Hydra 2.0 Web Portal. https://research.nhgri.nih.gov/hydra/. Accessed 1 Apr 2021.

55. OIST Marine Genomics Unit Genome Browser. https://marinegenomics.oist.jp/gallery. Accessed 1 Apr 2021.

56. MARIMBA. http://marimba.obs-vlfr.fr/. Accessed 1 Apr 2021.

57. IRIDIAN GENOMES. https://www.iridiangenomes.com/. Accessed 1 Apr 2021.

58. Li Y, Gao L, Pan Y, Tian M, Li Y, He C, et al.. Chromosome-level reference genome of the jellyfish *Rhopilema esculentum*. *GigaScience*. 2020; doi: 10.1093/gigascience/giaa036.

59. Galliot B, Schummer M. ‘Guessmer’ screening strategy applied to species with AT-rich coding sequences. *Trends Genet*. 1993; doi: 10.1016/0168-9525(93)90051-I.

60. Putnam NH, Srivastava M, Hellsten U, Dirks B, Chapman J, Salamov A, et al.. Sea anemone genome reveals ancestral eumetazoan gene repertoire and genomic organization. *Science*. 2007; doi: 10.1126/science.1139158.

61. Taguchi T, Tagami E, Mezaki T, Sekida S, Chou Y, Soong K, et al.. Recent progress of molecular cytogenetic study on scleractinian (stony) corals. *Kuroshio Sci*. 2017;11:73-81.

62. Technau U, Robb S, Genikhovich G, Montenegro J, Fropf W, Weinguny L, et al.. Sea anemone genomes reveal ancestral metazoan chromosomal macrosynteny. *Preprint*. 2021; doi: 10.21203/rs.3.rs-796229/v1.

63. Blommaert J. Genome size evolution: towards new model systems for old questions. *Proc R Soc B Biol Sci*. 2020; doi: 10.1098/rspb.2020.1441.

64. Wong WY, Simakov O, Bridge DM, Cartwright P, Bellantuono AJ, Kuhn A, et al.. Expansion of a single transposable element family is associated with genome-size increase and radiation in the genus *Hydra*. *Proc Natl Acad Sci*. 2019; doi: 10/ggdfjb.

65. Nong W, Cao J, Li Y, Qu Z, Sun J, Swale T, et al.. Jellyfish genomes reveal distinct homeobox gene clusters and conservation of small RNA processing. *Nat Commun*. 2020; doi: 10.1038/s41467-020-16801-9.

66. Kim H-M, Weber JA, Lee N, Park SG, Cho YS, Bhak Y, et al.. The genome of the giant Nomura's jellyfish sheds light on the early evolution of active predation. *BMC Biol*. 2019; doi: 10/gfxm7p.

67. Schrader L, Schmitz J. The impact of transposable elements in adaptive evolution. *Mol Ecol*. 2019; doi: 10.1111/mec.14794.

68. Cosby RL, Judd J, Zhang R, Zhong A, Garry N, Pritham EJ, et al.. Recurrent evolution of vertebrate transcription factors by transposase capture. *Science*. 2021; doi: 10.1126/science.abc6405.

69. Hamada M, Satoh N, Khalturin K. A Reference Genome from the Symbiotic Hydrozoan, *Hydra viridissima*. *G3 (Bethesda).* 2020; doi: 10.1534/g3.120.401411.

70. Xia W, Li H, Cheng W, Li H, Mi Y, Gou X, et al.. High-Quality Genome Assembly of *Chrysaora quinquecirrha* Provides Insights Into the Adaptive Evolution of Jellyfish. *Front Genet*. 2020; doi: 10.3389/fgene.2020.00535.

71. Graur D, Zheng Y, Azevedo RBR. An Evolutionary Classification of Genomic Function. *Genome Biol Evol*. 2015; doi: 10.1093/gbe/evv021.

72. Chapman J a, Kirkness EF, Simakov O, Hampson SE, Mitros T, Weinmaier T, et al.. The dynamic genome of *Hydra*. *Nature*. 2010; doi: 10.1038/nature08830.

73. Galliot B. Hydra, a fruitful model system for 270 years. *Int J Dev Biol*. 2012; doi: 10.1387/ijdb.120086bg.

74. Tomczyk S, Fischer K, Austad S, Galliot B. *Hydra*, a powerful model for aging studies. *Invertebr Reprod Dev*. 2015; doi: 10.1080/07924259.2014.927805.

75. Martín-Durán JM, Ryan JF, Vellutini BC, Pang K, Hejnol A. Increased taxon sampling reveals thousands of hidden orthologs in flatworms. *Genome Res*. 2017; doi: 10/f97kth.

76. Weisman CM, Murray AW, Eddy SR. Many, but not all, lineage-specific genes can be explained by homology detection failure. *PLOS Biol*. 2020; doi: 10.1371/journal.pbio.3000862.

77. Schriml LM, Chuvochina M, Davies N, Eloe-Fadrosh EA, Finn RD, Hugenholtz P, et al.. COVID-19 pandemic reveals the peril of ignoring metadata standards. *Sci Data*. 2020; doi:

10.1038/s41597-020-0524-5.

78. Toczydlowski RH, Liggins L, Gaither MR, Anderson TJ, Barton RL, Berg JT, et al.. Poor data stewardship will hinder global genetic diversity surveillance. *Proc Natl Acad Sci*. National Academy of Sciences; 2021; doi: 10.1073/pnas.2107934118.

79. Arita M, Karsch-Mizrachi I, Cochrane G, on behalf of the International Nucleotide Sequence Database Collaboration. The international nucleotide sequence database collaboration. *Nucleic Acids Res*. 2021; doi: 10.1093/nar/gkaa967.

80. Kodama Y, Shumway M, Leinonen R. The Sequence Read Archive: explosive growth of sequencing data. *Nucleic Acids Res*. 2012; doi: 10/fw3c92.

81. Wilkinson MD, Dumontier M, Aalbersberg IJ, Appleton G, Axton M, Baak A, et al.. The FAIR Guiding Principles for scientific data management and stewardship. *Sci Data*. Nature Publishing Group; 2016; doi: 10.1038/sdata.2016.18.

82. Schnitzler CE. What makes a jellyfish. *Nat Ecol Evol*. 2019; doi: 10/gfzg9d.

83. Smith SD, Pennell MW, Dunn CW, Edwards SV. Phylogenetics is the New Genetics (for Most of Biodiversity). *Trends Ecol Evol*. 2020; doi: 10.1016/j.tree.2020.01.005.

84. Ewels P, Magnusson M, Lundin S, Käller M. MultiQC: summarize analysis results for multiple tools and samples in a single report. *Bioinformatics*. 2016; doi: 10.1093/bioinformatics/btw354.

85. Challis R, Richards E, Rajan J, Cochrane G, Blaxter M. BlobToolKit – Interactive Quality Assessment of Genome Assemblies. *G3 (Bethesda).* 2020; doi: 10.1534/g3.119.400908.

86. Deakin JE, Potter S, O'Neill R, Ruiz-Herrera A, Cioffi MB, Eldridge MD, et al.. Chromosomics: Bridging the gap between genomes and chromosomes. *Genes*. Multidisciplinary Digital Publishing Institute; 2019;10:627.

87. Dimitrova M, Meyer R, Buttigieg PL, Georgiev T, Zhelezov G, Demirov S, et al.. A streamlined workflow for conversion, peer review, and publication of genomics metadata as omics data papers. *GigaScience*. 2021; doi: 10.1093/gigascience/giab034.

88. He S, Grasis JA, Nicotra ML, Juliano CE, Schnitzler CE. Cnidofest 2018: the future is bright for cnidarian research. *Evodevo*. 2019; doi: 10.1186/s13227-019-0134-5.

89. Fautin DG, Westfall JA, Cartwright P, Daly M, Wyttenbach CR. Coelenterate Biology 2003: Trends in Research on Cnidaria and Ctenophora. *Hydrobiologia*. 2005;530:11–3.

90. Juliano CE, Hobmayer B. Meeting report on “animal evolution: New perspectives from early emerging metazoans”, tutzing, september 14–17, 2015. *BioEssays*. 2016; doi: 10.1002/bies.201500200.

91. Santander MD, Maronna MM, Ryan JF, Andrade SCS. The state of Medusozoa Genomics: supplementary material. doi: 10.6084/m9.figshare.17155676. Private link https://figshare.com/s/cd5e8069fb33cdb5a7aa

92. Hydractinia Genome Project Portal. https://research.nhgri.nih.gov/hydractinia/. Accessed 1 Apr 2021.

93. Vogg MC, Beccari L, Iglesias Ollé L, Rampon C, Vriz S, Perruchoud C, et al.. An evolutionarily-conserved Wnt3/β-catenin/Sp5 feedback loop restricts head organizer activity in Hydra. *Nat Commun*. Nature Publishing Group; 2019; doi: 10.1038/s41467-018-08242-2.

94. Xia W-X, Li H-R, Ge J-H, Liu Y-W, Li H-H, Su Y-H, et al.. High-continuity genome assembly of the jellyfish *Chrysaora quinquecirrha*. *Zool Res*. 2021; doi: 10.24272/j.issn.2095-8137.2020.258.

Table 1 - Genomic projects related to Medusozoa HTS. Sequencing projects with no current related publication are remarked with capital letters. Column "Main research topics" describes keywords according to references, restricted to a maximum of 4; "gene evolution" refers to the study of gene gains/losses and also of specific gene families. Species with reported assemblies were re-analyzed in this review (bold; Supplementary file S3 Table S3). UMCG=University Medical Center Groningen; IISER PRune=Indian Institute of Science Education and Research, Pune; NHGRI=The National Human Genome Research Institute; TF=transcription factors; *"preliminary” assembly available at the institutional site; **species with taxonomic updates. For further details see Supplementary file S1.

Project	Release year (NCBI-SRA)	Class (n° genomes)	Species	Main research topics
Chapman et al. [72]	2008	Hydrozoa (1)	***Hydra vulgaris***	Gene evolution; micro-synteny
IISER Pune	2014-2015	Hydrozoa (1)	***Hydra vulgaris***	not_informed
NHGRI [54]	no SRA	Hydrozoa (1)	***Hydra vulgaris***	not_informed
NHGRI [92]	2016	Hydrozoa (1)	*Hydractinia echinata**	not_informed
Gold et al. [19]	2018	Scyphozoa (1)	***Aurelia coerulea***	Life cycle; gene evolution; intraspecies variability; HOX
IRIDIAN GENOMES [57]	2018	Hydrozoa (1)	***Craspedacusta sowerbii***	not_informed
Kim et al. [66]	2018	Scyphozoa (1)	***Nemopilema nomurai***	Life cycle; jellyfish body patterning; gene evolution; toxins
IRIDIAN GENOMES [57]	2019	Hydrozoa (1)	*Scolionema suvaense*	not_informed
Khalturin et al. [20]	2019	Scyphozoa (2)	***Aurelia aurita**, Aurelia coerulea****	Life cycle; jellyfish body plan; gene evolution; synteny
		Cubozoa (1)	***Morbakka virulenta***	
Leclère et al. [21]	2019	Hydrozoa (1)	***Clytia hemisphaerica***	Life cycle; gene evolution; micro-synteny; TF
Odhera et al. [22]	2019	Scyphozoa (1)	***Cassiopea xamachana***	Gene evolution; micro-synteny; Homeobox; toxins
		Cubozoa (1)	***Alatina alata***	
		Staurozoa (1)	***Calvadosia cruxmelitensis***	
Vogg et al. [93]	2019	Hydrozoa (1)	***Hydra oligactis*; *Hydra viridissima***	Gene evolution; RTKs; developmental genes
Hamada et al. [69]	2020	Hydrozoa (1)	***Hydra viridissima***	Symbiosis; immune response; repetitive DNA; Homeobox
IRIDIAN GENOMES [57]	2020	Cubozoa (3)	***Alatinidae* sp.**	not_informed
			Carybdea marsupialis	

			Tamoya ohboya	
			Cladonema radiatum	
		Hydrozoa (2)	*Eutima* sp. BMK-2020	
			Aurelia coerulea	
			Chrysaora achlyos	
			Chrysaora chesapeakei	
		Scyphozoa (4)	***Chrysaora fuscescens***	
		Staurozoa (1)	*Calvadosia cruxmelitensis*	
Li et al. [58]	2020	Scyphozoa (1)	***Rhopilema esculentum***	Gene evolution; toxins
Nong et al. [65]	2020	Scyphozoa (2)	***Sanderia malayensis, Rhopilema esculentum***	Gene evolution; small RNAs; micro-synteny; Homeobox
Xia et al. [70]	2020	Scyphozoa (1)	***Chrysaora quinquecirrha***	Gene and gene feature evolution; repetitive DNA
Xia et al. [94]	2020	Scyphozoa (1)	***Chrysaora quinquecirrha***	Assembly improvement report
UMCG	2021	Scyphozoa (1)	***Cassiopea andromeda***	not_informed

Figure 1 - Phylogenetic distribution of genomic information in Medusozoa. A) Number of described species and number of species with genomic data; B) Chromosome number (2n) range; C) Genome size (Mbp) range taking into account Flow Cytometry and Feulgen Densitometry estimations; D) Total number of available assemblies and number of species with assembled genomes. In B) and C) single values were also included when only one species was characterized. Tree topology is explained in the methods section. Information used for this graph is available at Supplementary file S3 Table S2.

Figure 2 - Assembly and genome features. In A) is reported (from left to right): mean assembly length per class, GC content (%) per class, number of contigs and scaffolds per assembly coloured by class, contig and scaffold N50 (in Kbp) per assembly coloured by class,

and count of assemblies of each class corresponding to the different BGP-metric values, where X and Y correspond to contig and scaffold N50 respectively, and Z to chromosome assignment (see methods section). In B) is reported (from left to right): mean repeat length (Mbp) in assembly per class, mean total number of genes per class, mean exon number (count per gene) per class, and mean gene, intron and exon length (Kbp) per assembly coloured by class. The yellow arrowhead indicates *S. malayensis* gene features (See Box). All other references are specified in the figure. Mbp=millions of base pairs. Information used for this graph is available at Supplementary file S3 Tables S4-6.

Figure 3 - BUSCO Metazoa gene distribution in Medusozoa assemblies. Each column corresponds to a gene and each row an assembly. Columns were ordered based on presence from left to right and the least present genes (n=96) are shown in detail. Genes absent in all or almost all assemblies (more than 80% of absence) are indicated in red; genes also reported absent [20] are indicated in bold; genes absent in specific lineages are indicated with yellow rectangles. Higher quality assemblies are indicated in orange (BGP-metric > 1.0.0). The assembly with the highest quality score for BGP-metric is indicated by an orange circle and corresponds to *Rhopilema esculentum* [58]. Information used for this graph and complete genes names are available at Supplementary file S3 Table S7.

Supplementary Material

Supplementary file S1. Dataset 1. Genome report sheet.

Supplementary file S2. Table S1. Species information considering chromosome number, genome size and genomic datasets.

Supplementary file S3. Supplementary tables 2-8 - All information used for constructing graphs presented in this work. Includes summary information of Figure 1 (table S2), genome resources used in this study (table S3), assembly statistics for Figure 2A (table S4), genome

features of Figure 2B (table S5, S6) and BUSCO results for Figure 3 and Supplementary figure S4 (tables S7, S8).

Supplementary file S4. - BUSCO Eukaryota gene distribution in Medusozoa assemblies. Each column corresponds to a gene and each row an assembly. Information used for this graph is available at Supplementary file S3 Table S8.

Supplementary file S5 - Dataset 2. Information and metadata obtained from NCBI.

Supplementary file S6 - Dataset 3. Original results from AGAT and Galaxy server (BUSCO).

Supplementary file S7 - Dataset 4. Command line to retrieve data from NCBI and to generate new results.

Supplementary file S8 - Dataset 5. Figures in vectorial format

Figure 1

Click here to access/download;Figure;1 Figure 1.TIFF

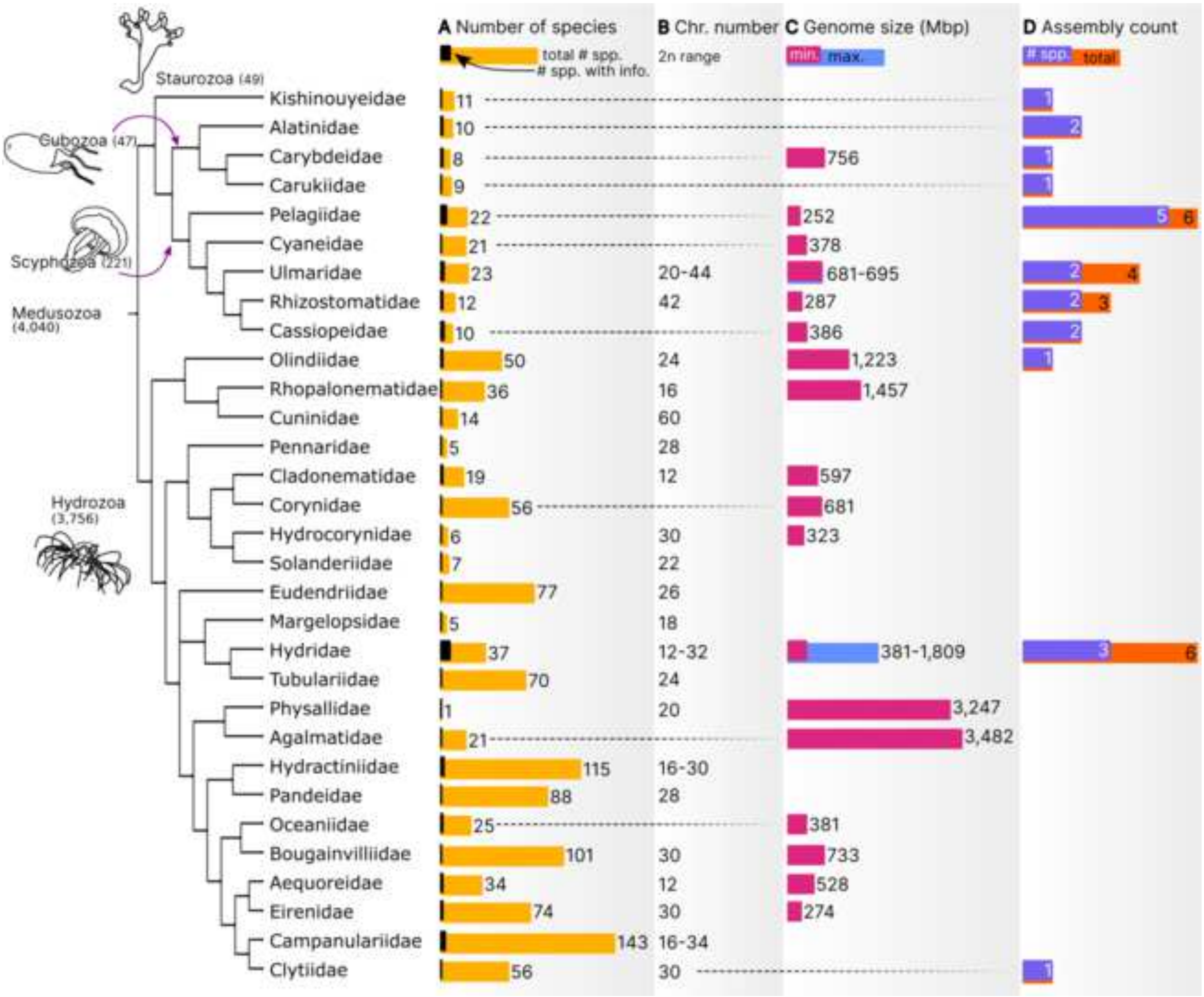

Figure 2

Click here to access/download;Figure;2 Figure 2.TIFF

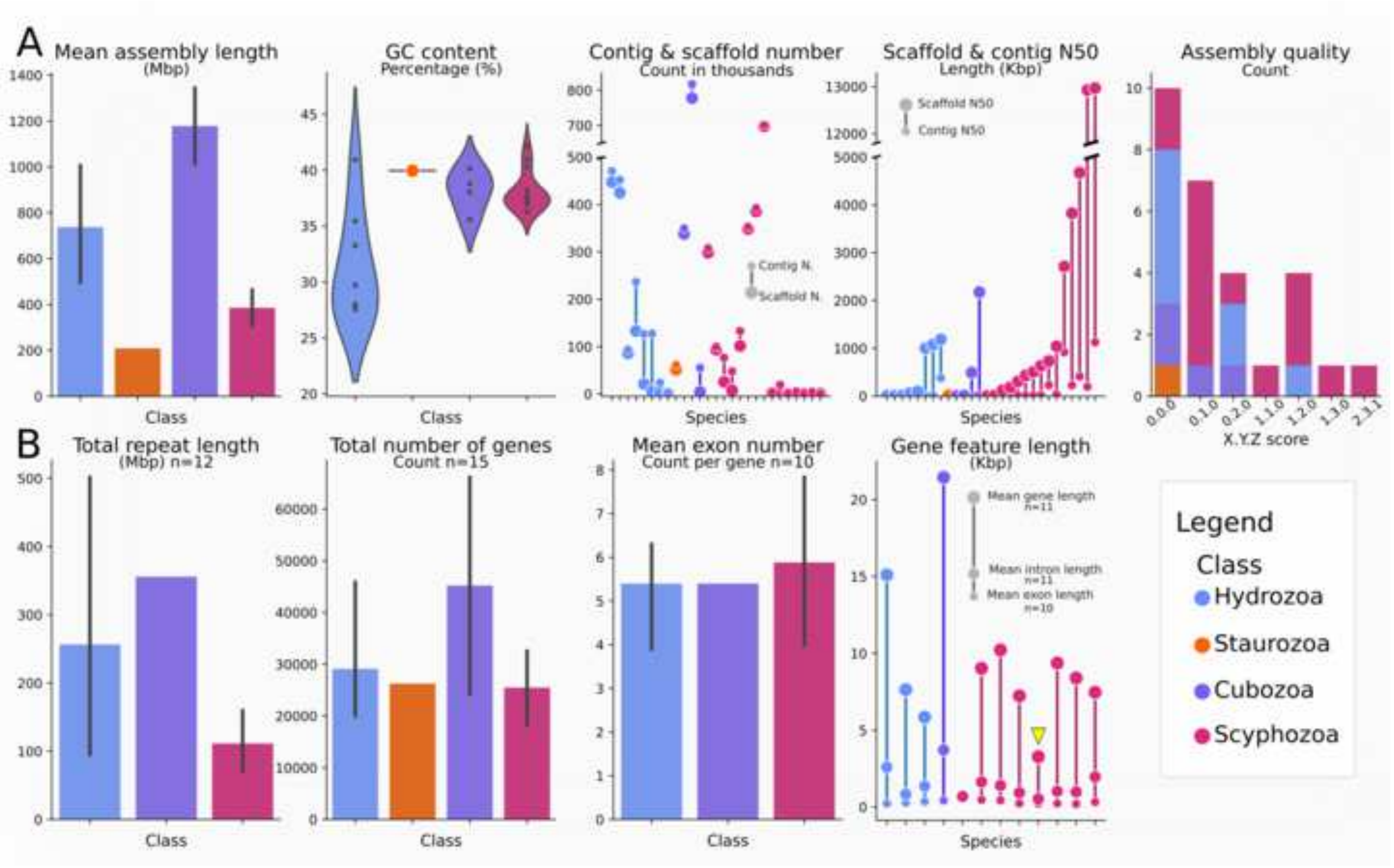

Figure 3

Click here to access/download;Figure;3 Figure 3.TIFF

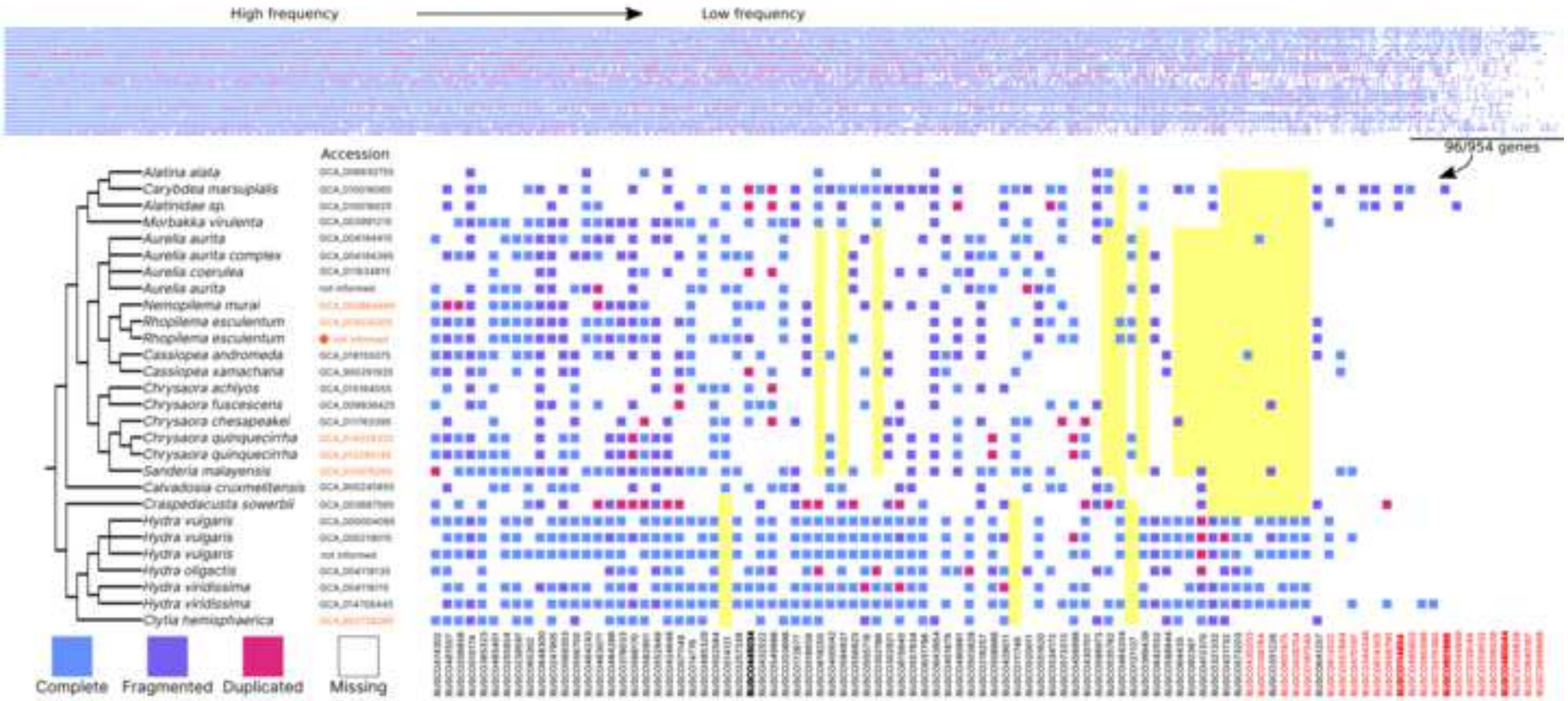

Click here to access/download
Supplementary Material
Supplementary_file_S1_Dataset_1_genome_report_sheet.xlsx

Supplementary File 2

Click here to access/download

Supplementary Material

Supplementary_file_S2_Table_S1.xlsx

Supplementary File 3

Click here to access/download
Supplementary Material
Supplementary_file_S3_TablesS2-8_Figures_Information.xlsx

Supplementary File 4

Click here to access/download
Supplementary Material
Supplementary_file_S4.png

Supplementary File 7

Click here to access/download
Supplementary Material
Supplementary_file_S7_Dataset_4_command_line.html
