# Supplementary material for: The state of Medusozoa genomics: current evidence and future challenges
Source: Gigascience. 2022 May 17;11:giac036. doi: 10.1093/gigascience/giac036 (PMC9112765; doi:10.1093/gigascience/giac036)
Supplement: giac036_GIGA-D-21-00404_R1_Revision_1 [file giac036_giga-d-21-00404_r1_revision_1.pdf]

GigaScience

The state of Medusozoa genomics: current evidence and future challenges

--Manuscript Draft--

**Manuscript Number:**	GIGA-D-21-00404R1
**Full Title:**	The state of Medusozoa genomics: current evidence and future challenges
**Article Type:**	Review
**Funding Information:**	Coordenação de Aperfeiçoamento de Pessoal de Nível Superior (88882.377420/2019-01) — Miss Mylena Daiana Santander<br>Fundação de Amparo à Pesquisa do Estado de São Paulo (FAPESP 2016/04560-9) — Mr. Maximiliano Manuel Maronna<br>Fundação de Amparo à Pesquisa do Estado de São Paulo (FAPESP 2015/20139-) — Miss Sónia Cristina da Silva Andrade
**Abstract:**	Medusozoa is a widely distributed ancient lineage that harbors one-third of Cnidaria diversity divided into four classes. This clade is characterized by the succession of stages and modes of reproduction during metagenic lifecycles, and includes some of the most plastic body plans and life cycles among animals. The characterization of traditional genomic features, such as chromosome numbers and genome sizes, was rather overlooked in Medusozoa and many evolutionary questions still remain unanswered. Modern genomic DNA sequencing in this group started in 2010 with the publishing of the Hydra vulgaris genome and has experienced an exponential increase in the past three years. Therefore, an update of the state of Medusozoa genomics is warranted. We reviewed different sources of evidence, including cytogenetic records and high-throughput sequencing (HTS) projects. We focused on four main topics that would be relevant for the broad Cnidaria research community: 1) taxonomic coverage of genomic information; 2) continuity, quality and completeness of HTS datasets; 3) overview of the Medusozoa specific research questions approached with genomics; and 4) the accessibility of data and metadata. We highlight a lack of standardization in genomic projects and their reports, and reinforce a series of recommendations to enhance future collaborative research.
**Corresponding Author:**	Mylena Daiana Santander, Bachelor<br>Universidade de Sao Paulo Instituto de Biociencias<br>São Paulo, SP BRAZIL
**Corresponding Author Secondary Information:**	
**Corresponding Author's Institution:**	Universidade de Sao Paulo Instituto de Biociencias
**Corresponding Author's Secondary Institution:**	
**First Author:**	Mylena Daiana Santander, Bachelor
**First Author Secondary Information:**	
**Order of Authors:**	Mylena Daiana Santander, Bachelor<br>Maximiliano Manuel Maronna, PhD<br>Joseph F Ryan, PhD<br>Sónia Cristina da Silva Andrade, PhD
**Order of Authors Secondary Information:**	
**Response to Reviewers:**	Editor<br><br>"Your manuscript "The state of Medusozoa genomics: past evidence and future challenges" (Review Article; GIGA-D-21-00404) has been assessed by three reviewers. Based on these reports, I am pleased to inform you that it is potentially

Powered by Editorial Manager® and ProduXion Manager® from Aries Systems Corporation

	acceptable for publication in GigaScience, once you have carried out some essential revisions suggested by our reviewers. Their reports are below.<br>I'd like to highlight three points:"<br><br>We are very appreciative of the excellent suggestions from the reviewers and editor. We have done our best to address each point and we feel that the manuscript has been greatly improved as a result of the review process. Thank you for the time dedicated to our manuscript. We provide a point-by-point answer to each suggestion. We also provide a new main text and a copy of the original text with all the changes kept as tracks. Line numbers in this letter are referenced to the new main text file in de submission PDF. Original comments made by the editor and the reviewers are indicated in bold or between quotation marks. We also provide a formated copy of the response to the reviewers as a separate file at the end of the submission PDF.<br><br>"1. Two of the reviewers mention that the "recommendations" would benefit if it would make clearer if there are any Medusozoa-specific recommendations (in addition to advice that is generally applicable to all animal genome projects)"<br><br>We have added the following to address this point generally on line 422:<br><br>The following are suggestions to enhance genome projects and outcomes, and to promote open and collaborative research. These suggestions can be broadly applied to any genome project and are in line with those proposed by many initiatives and consortia (e.g. [33,100,101]). Nevertheless, it is worth reinforcing and discussing them in the context of this review since genome projects are more and more often being initiated in research laboratories that have historically been more focused on other aspects of medusozoan biology and may not be as familiar with these general practices:<br><br>We have added the following to point #3 that refers to where to deposit data on lines 446:<br><br>A Medusozoa-centric database with long-term maintenance is still lacking for the community (e.g. Mollusca clade [104]); but many open repositories can serve this purpose with low or no costs considering the size of the aforementioned outputs. There are open topic-centric repositories (e.g Dfam [105] for repetitive DNA), general repositories (e.g. FigShare, Zenodo; or even NCBI for annotation tracks) as well as personal or institutional ones. Many of the reviewed genomic projects already made use of these repositories but failed to deposit some of the outputs. A solution for this inconvenience is to update submissions or create novel ones (e.g. submit annotations to NCBI or ENA) to deposit the missing outputs.<br><br>"2. Reviewer 1 recommends to make your code public, and I strongly support this, as it is also in line with our journal guidelines. You can also host code and supporting data in our repository GigaDB - our data curators will be happy to help. Please attach an open (OSI-compliant) licence to any scripts/code. (https://opensource.org/licenses)"<br><br>All the command lines used in this work were originally specified in the Supplementary File S7 of the original submission (Supplementary File S2 in the current version) but it was not properly indicated in the material and methods section. We corrected this issue by adding the following sentence on lines 122:<br><br>The command line used for retrieving genetic information and metadata, for statistics calculation and the code used for graph generation are available at Supplementary file S2 and S3.<br><br>We have also added the scripts used for constructing graphs in Supplementary file S3 (as suggested by reviewer 1). All the software used in this work is open and was properly referenced.<br><br>We deposited all supplementary files in Figshare and GigaDB and included a statement of open license to scripts on lines 518:

Powered by Editorial Manager® and ProduXion Manager® from Aries Systems Corporation

Data availability
All collected information, outputs and scripts supporting new results are available in the supplementary files S1-S9 in Figshare [114] and in GigaDB [115].

"3. Although not mentioned by the reviewers, I feel your manuscript would be more interesting for readers from outside the medusozoa community if you explained in a bit more detail the actual biological questions that have been addressed with these genomes; such as toxins, metazoan evolution / body plan evolution, Hox genes, immunity, etc.. These topics are mentioned in the introduction, but I feel they could be picked up again in a bit more detail in the discussion, to illustrate the biological insights gained from the genome projects."

We have added two paragraphs that highlight the insight genome projects bring understanding medusozoa biology.

Starting on line 301:

The complex nature of Medusozoa venom has been investigated by a number of transcriptomic, proteomic and genomic studies (reviewed in [26]). Several putative toxin genes and domains have been identified, covering a significant part of the wide range of known toxins [20,22,59,73]. In Scyphozoa, toxin-like genes were often recovered as multicopy sets [20,59]. Moreover, in R. esculentum toxin-like genes were also tandemly arranged and several of them were located nearby in chromosome 7, suggesting that the observed organization might influence toxin co-expression[59]. Minicollagens, which are major components of nematocysts, also had a clustered organization and a pattern of co-expression in Aurelia [20]. These examples add to various clustered genes described in Cubozoa, Hydrozoa and Anthozoa, and would indicate that gene clustering and operon-like expression of toxin genes is widespread in Cnidaria ([20] and references therein).

and starting on line 329:

The complex life cycle of Medusozoa has resulted from the combination of both ancestral and novel features. Aurelia, Morbakka virulenta and Clytia hemisphaerica have significantly different patterns of gene expression across stages and during transitions [19–21]. Differentially expressed genes include many conserved ancestral families of transcription factors [19–21]; there is also a considerable amount of the putative lineage-restricted genes that show differential expression in the adult stages [20,21]. A few of these “novel” medusozoan genes have been described, such as novel myosin-tail proteins that are absent from Anthozoa and represent markers of the medusae striated muscles [20]. It was suggested that the evolution of the Medusozoa complex life cycle would therefore have involved the rewiring of regulatory pathways of ancestral genes and the contribution of new ones [19–21]. As such, the body plan and life cycle simplifications observed in Clytia and Hydra, respectively, would be the result of loss of transcription factors involved in their development [21]. Finally, the significance of many of the putative Medusozoa and species-specific genes remain to be elucidated.

"4. For a review article, please also feel free to add illustrations/photos of relevant medusozoa species, if you wish (but please check with any copyright holder, if applicable - images will be published under an open cc-by licence)."

We added a new figure (Figure 1) with photographs of example species of each Medusozoa class. Some photographs (Figure 1 A, B, D, E) were recovered from an online open database called Cifonauta, available under open cc-by license, and it was properly cited. The remaining photographs were provided by Marta Chiodin (Figure 1C), Joseph Ryan (co-author; Figure 1 F, G), with permission to publish under CC-BY license. As a result of the addition of a new Figure 1, all figures were renumbered accordingly.

Reviewer 1
"In this paper, Santander et al. review the field of medusozoan genomics, which has burgeoned in the last three or so years. Overall, I found this a clear, interesting read. The manuscript is well-written, the figures are valuable, and the authors nicely describe

the history of the research as well as the state of the field. The findings are not monumental, but it is a worthwhile exercise to survey the rapidly-increasing dataset of genomes in a systematic way, and this review will be a useful start for further work in medusozoan comparative genomics. I rarely suggest a paper should be accepted during the first round of review, and I usually try to provide more constructive feedback than I do here, but I really don't have much too much to quibble with. A couple thoughts are provided below:

1. The set of suggestions for future work near the end of the document are fine, but they could apply broadly to any genome project. I encourage the authors to consider whether there are specific problems related to medusozoan evolution that are hampered by inconsistencies between studies, and discuss how their recommendations (or additional ones) could help resolve them."

This comment also addresses reviewer #3's first point as well. We have added the following, which acknowledges that some of our recommendations are general to all genome projects and provides justification for why it is important to include these in this review on line 422:

The following are suggestions to enhance genome projects and outcomes, and to promote open and collaborative research. These suggestions can be broadly applied to any genome project and are in line with those proposed by many initiatives and consortia (e.g. [33,100,101]). Nevertheless, it is worth reinforcing and discussing them in the context of this review since genome projects are more and more often being initiated in research laboratories that have historically been more focused on other aspects of medusozoan biology and may not be as familiar with these general practices:

In the recommendation regarding depositing results in public databases we discussed its importance and how metadata can be improved when datasets were already made public on line 431:

Frequently, data and metadata that are described in the original articles or deposited in repositories are not submitted to public databases. Tracking information from multiple sources is time consuming and prone to error. Databases and repositories enable the improvement of metadata after the initial releases, by the addition of new or corrected information (e.g. publication information) from the authors. We believe that this kind of data curation would improve the state of Medusozoa genomics not only by enabling downstream analysis after the publication, but also enabling the detection of methodological options (e.g. tissue selection; sequencing technology) that would improve the quality of the results.

In the section about depositing intermediate outputs, we have added information on the state of relevant taxon-specific databases on line 446:

Medusozoa-centric database with long-term maintenance is still lacking for the community (e.g. Mollusca clade [104]); but many open repositories can serve this purpose with low or no costs considering the size of the aforementioned outputs.

We added a paragraph discussing potential problems and benefits related to proper method description on line 460.

The latter suggestions (3-6) are mainly related to providing detailed methodologies of bioinformatic analyses. First, proper method and results descriptions can help to recover metadata and criteria usually not available in large sequence repositories. Second, comparative analyses depend upon standardization at different levels and significant sample sizes. The inclusion of species in downstream analyses is limited by data availability and proper description of previous analyses, custom software and results.

We added a recommendation about engaging in community-wide discussions, and highlighted potential venues that would be appropriate for discussing medusozoan genomics standards starting on line 466:

Powered by Editorial Manager® and ProduXion Manager® from Aries Systems Corporation

7. Engage in community-driven conversations about standards, guidelines and species priorities. There are a number of taxon-specific meetings that would be appropriate venues to engage in these conversations including the International Conference on Coelenterate Biology (~decennial; [106]), the International Jellyfish Blooms Symposium (~triennial), Cnidofest (~biennial; [107]), Tutzing workshop (~biennial; [108]), and Cnidofest zoom seminar series. In addition, satellite meetings at larger annual meetings (e.g. the Society for Integrative and Comparative Biology (SICB) or the Global Invertebrate Genomics Alliance (GIGA [101])) could provide appropriate venues to facilitate discussions on how the community can best move forward as more and more genomic data come online.

We close the section with a paragraph that explains how adhering to standards will benefit the medusozoan community on line 475:

The adoption of best practices in the Medusozoa genomics community will pave the way for major breakthroughs regarding understanding the genomic basis for several evolutionary innovations that arose within and in the stem lineage of Medusozoa. Similar advances were achieved with extensive taxon sampling at broader scales, where 25 novel core gene groups enriched in regulatory functions might be underlying the emergence of animals [109,110]. Medusozoa innovations have puzzled the community for decades [5,7,11,111] and include the origin of the medusa, the loss of polyp structures, the establishment of symbiosis, the blooming potential, and the evolution of an extremely potent venom. A deeper understanding of the genomic events driving these innovations will require accurate identifications of a number of key genomic features including (but not limited to) single copy orthologs, gene losses, lineage-specific genes, gene family expansions and non-coding regulatory sequences.

Related to this last point, we also suggest to read the added sentences after reviewer #3 comment on line 314:

Recent evidence proved that the detection of lineage-specific genes, and other analyses relying on accurate annotation and orthology prediction, can be significantly biased by methodological artifacts [79–83]; several problems have been identified, such as low taxon sampling, heterogeneous gene predictions, and failure of detecting distant homology and fast-evolving orthologues. These considerations are highly relevant in Medusozoa, as comparisons are often made, by necessity, with distantly related species (e.g. Anthozoa has been estimated to have diverged from Medusozoa around 800 million years ago [84]).

"2. I would encourage the authors to practice what they preach in terms of transparency, and make the code they used in their methods public (e.g. statswrapper.sh, AGAT, BUSCO, ETE Toolkit, Matplotlib, Seaborn). The code does not need to be executable, but a supplemental text and/or repository with as much of the starting data and commands executed as possible would make it easier for others to replicate this work and apply it to future comparative genomics projects."

All the command line used in this work was originally specified in the Supplementary S7 of the original submission but we did not not properly indicate this in the material and methods section. We corrected this issue by adding a sentence in the corresponding section as indicated below (note: this required re-numbering the supplementary files so Supplementary file S7 is now S2). We also included the scripts used for constructing graphs. All the packages and softwares used in the command line and in the custom scripts (statswrapper.sh, AGAT, BUSCO, ETE Toolkit, Matplotlib, Seaborn) are open. We have added the following on line 122:

The command line used for retrieving genetic information and metadata, for statistics calculation and the code used for graph generation are available at Supplementary file S2 and S3.

"3. Line 236: "…ploidy level, heterochromatin contente." This should be changed to "…ploidy level, and heterochromatin content.""

This error was corrected.

Powered by Editorial Manager® and ProduXion Manager® from Aries Systems Corporation

"4. Line 253-254: "…evolution of genome size is a long-standing question that is included in the so-called C-value Enigma [40]." The authors provide a citation, but I think this sentence would be stronger with a brief explanation of what the C- value Enigma is. Medusozoans are a great example of this "enigma", so it's worth reinforcing."

We have added the following to clarify the C-value enigma on line 274:

… "C-value Enigma" [41]. This name stems from the difficulty elucidating the evolutionary forces (e.g. drift and natural selection) that have given rise and serve to maintain variations in genome size, the mechanisms of genome size change, and the consequences of these variations at an organismal level [41]. Several conflicting hypotheses have been postulated to explain this puzzle with most having experimental support in some but not all lineages (reviewed in [68]).

Reviewer 2

"This manuscript offers a reanalysis of all available nuclear genomic data published on medusozoans. It represents a well though, and timely review of the available data, systematically comparing genomic features (repeated elements, intro/exon/gene size and numbers, chromosome numbers...) and genomic assemblies (available data, assembly quality and size…) in the different medusozoan classes. It largely confirms the results obtained from analysis of single species. It also provides useful guidelines for future standardization of genomic projects focused on medusozoans."
Minor comments and suggested corrections:
1. Line 118: How was "compiled all genomic and HTS metadata reference in this review", manually? If not, please provide the scripts used for this task."

The information was collected by a combination of automatic and manual retrieval, as it was superficially mentioned in the first paragraph of the Material and Methods section. We added a few sentences to clarify this point as follows below. All of the command lines used for these analyses were originally specified in the Supplementary S7 of the original submission but this was not properly indicated in the material and methods section. We corrected this issue by adding a sentence in the corresponding section as indicated below (note: this required re-numbering the supplementary files so Supplementary file S7 is now S2).

First, we clarified the automatic and manual retrieval on line 91:
Our main source of genomic information and metadata was NCBI Genome (Assembly, Genomes, Nucleotide, Taxonomy and SRA; [27]). We retrieved data automatically using entrez-direct v.13.9 and NCBI datasets v. 12.12. For information not present in NCBI, we checked published articles for proper information collection, as well as personal repositories mentioned in the associated articles.
We clarified that the merging of manually and automatically retrieved information was merged/compiled manually, and specified the supplementary material where scripts and command lines were deposited on line 119:
We manually compiled all genomic information and HTS metadata referenced in this review using a report model based on previous works and public databases such as NCBI (Supplementary file S1; [29,41,42]). The command line used for retrieving genetic information and metadata, for statistics calculation and the code used for graph generation are available at Supplementary file S2 and S3.

"2. Line 236: correct contente"

This error was corrected.

"3. Line 326: The sentence starting with "Moreover, even…" is unclear. Please clarify or delete."

To clarify this point we deleted the original sentence and added the following on line 403:
In addition, submission to the large databases like SRA and GenBank can lead to the automatic detection of specific issues such as contamination or annotation errors that might otherwise not be detected.

Powered by Editorial Manager® and ProduXion Manager® from Aries Systems Corporation

"4. Line 389: correct "proyects""

This error was corrected.

"5. Figure 1: it would useful to indicate in this figure genome sizes calculated from genomic assemblies, in addition to genome sizes calculated from flow cytometry and feulgen densitometry estimations; either as a new column or using another color in C"

We prefer to maintain the original version of the figure. The following reasons were considered for not adding “assembly length” in figure 1 (now renumbered as Figure 2):

• Assembly length would not be a robust estimation of genome size because different causes can lead to biased results, especially for short reads projects. High heterozygosity and incomplete collapsing of haplotypes can lead to genome size overestimation. Sequencing bias, as well as repetitive DNA misassembly, can lead to underestimations of genome size (see https://doi.org/10.1371/journal.pone.0062856; 10.1111/1755-0998.12933; https://doi.org/10.1101/2021.04.09.438957; for further details)

• Adding this information in Figure 1 (now renumbered as Figure 2) could hinder visualization as already many variables are being simultaneously plotted.

• Distribution of assembly length was specified in Figure 2a (now renumbered as Figure 3a).

"6. SM_Table2: Suplementary Material S2 - Table S1 - please correct in the title "condidering"."

This error was corrected.

Reviewer 3

"Santander et al. review the state of genome assemblies and cytogenetics of Medusozoa. This review captures the progression of the sequencing efforts in the past decade and how the field is moving with new technological advances. From their assessment of the literature and unpublished data, they found that a weakness in their community is a general lack of standardization in analysis and limited availability of intermediate assembly components, such as the repeat libraries, and associated metadata. In the end they provide recommendations for standards to be applied to ongoing and future genomic projects.

1. I felt that these recommendations fell short of extending beyond basic requirements of publishing genomes today. While these recommendations are in line with recommendations of other genomic consortia (Vertebrate Genomes Project [Rhieet al. 2021, Nature], Sanger/Moore Aquatic Symbiosis Genomics, etc.) and most publishers including GigaScience (deposit data, reproducible methods, code availability statements, etc), they are quite general. I was left wondering if this was a commentary on the whole field of genomics. "

Reviewer #1 had a very similar comment. We have added the following, which acknowledges that some of our recommendations are general to all genome projects and provides justification for why it is important to include these in this review on lines 422:

The following are suggestions to enhance genome projects and outcomes, and to promote open and collaborative research. These suggestions can be broadly applied to any genome project and are in line with those proposed by many initiatives and consortia (e.g. [33,100,101]). Nevertheless, it is worth reinforcing and discussing them in the context of this review since genome projects are more and more often being initiated in research laboratories that have historically been more focused on other aspects of medusozoan biology and may not be as familiar with these general practices:

"2. To that end, are there specific recommendations regarding medusozoans that would enhance data usage community wide that could be stated here? "

As a response to point, which was also raised by reviewer #1 we added several sentences and paragraphs. Specifically, the manuscript now includes a discussion of how curational steps on database metadata could enhance data usage. It also includes a discussion about the lack of taxon-specific databases appropriate for Medusozoa, which may inspire such an effort in the near future. In addition, our recommendation that conversations regarding the state of medusozoan genomics take place at taxon-specific meetings should lead to enhanced data usage.

On line 431:

Frequently, data and metadata that are described in the original articles or deposited in repositories are not submitted to public databases. Tracking information from multiple sources is time consuming and prone to error. Databases and repositories enable the improvement of metadata after the initial releases, by the addition of new or corrected information (e.g. publication information) from the authors. We believe that this kind of data curation would improve the state of Medusozoa genomics not only by enabling downstream analysis after the publication, but also enabling the detection of methodological options (e.g. tissue selection; sequencing technology) that would improve the quality of the results.

On line 446:

A Medusozoa-centric database with long-term maintenance is still lacking for the community (e.g. Mollusca clade [94]); but many open repositories can serve this purpose with low or no costs considering the size of the aforementioned outputs.

On line 466:

7. Engage in community-driven conversations about standards, guidelines and species priorities. There are a number of taxon-specific meetings that would be appropriate venues to engage in these conversations including the International Conference on Coelenterate Biology (~decennial; [106]), the International Jellyfish Blooms Symposium (~triennial), Cnidofest (~biennial; [107]), Tutzing workshop (~biennial; [108]), and Cnidofest zoom seminar series. In addition, satellite meetings at larger annual meetings (e.g. the Society for Integrative and Comparative Biology (SICB) or the Global Invertebrate Genomics Alliance (GIGA [101])) could provide appropriate venues to facilitate discussions on how the community can best move forward as more and more genomic data come online.

We also provided a link in the data availability statement to the online version of the Supplementary file 1 in Figshare. This table will be maintained and can be modified/corrected if authors from the original papers contact us. On line 522:

A copy of table S1 will be available upon publication [114] and can be updated upon the original author’s request.

"3. Are there established assembly pipelines (i.e. tools that provide the highest quality assemblies from various species) or types of sequencing effort (i.e. long read + HiC maps, transcriptome-informed gene annotation) that should be endorsed as part of your assessment?"
A rigorous assessment of this issue was not possible because Medusozoa genomic datasets are quite heterogeneous (time-scales, technologies, objectives, methods and output quality; all with a small sampling). However, it is a highly relevant topic, and we opted to mention general trends in the main text with a proper citation to more specific bibliography on methods. We added the following paragraph on line 237:

Differences in sequencing strategy and platforms are expected to be linked with assembly quality, both in terms of continuity and completeness. For example, hybrid sequencing plus optical maps and combined evidence-based annotation should generate better results than a short-read sequencing and single-evidence annotation [61,62]. Although this general trend was observed in this review, with most Illumina-only datasets showing lower BGP-metric (Figure 3) and lower completeness (Figure 4),

Powered by Editorial Manager® and ProduXion Manager® from Aries Systems Corporation

it is not a granted condition. Some punctual cases can exemplify biological and methodological issues that impose limitations to genome sequencing and assembly: e.g. the difficulty in obtaining chromosome-scale assemblies despite small genome sizes and combined sequencing strategies (Hi-C + short reads+ long reads) [63,64] or the difficulty in extracting high-molecular-weight DNA [20]. Because of the heterogeneity of Medusozoa genomic projects in terms of time periods, objectives, methods and resources, a proper quantitative analysis of the relationship between methods and outcome quality would not be feasible, and we prefer to refer to articles specialized in assessing methods (e.g. [61,62]).

"4. Are there specific taxonomic gaps that should be prioritized (starting Line 238)?"

There are taxonomic gaps in Medusozoa genomics that were mentioned in the "Genomic projects: whos and hows of Medusozoa" section. But we believe criteria for priority should come from community discussions as was carried on by other projects. To remark the importance of filling taxonomic gaps, we added the following sentences on line 466:

7. Engage in community-driven conversations about standards, guidelines and species priorities.

And on line 501:

The distribution of genetic and genomic information presented significant taxonomic gaps in Medusozoa. It is a reasonable scenario since genomic sequencing data is accumulating in many medusozoan lineages. Even so, some of the most species-rich clades with a diverse array of phenotypic and ecological traits have not yet had their genomes sequenced (e.g. Scyphozoa:Coronamedusae, Hydrozoa:Macrocolonia). These, and other, heretofore genomically underexplored lineages provide golden opportunities from which to make major contributions to understanding the evolution of Medusozoa genomes and would be a wonderful contribution to the rest of the Medusozoa research community. Defining candidate species for sequencing can avoid unnecessary doubled efforts. Different international projects recognized this situation and proposed a set of criteria for prioritizing species at other scales, such as the GIGA ([101]).

"5. The majority of the resources you identified only have short-read Illumina data which inevitably means that chromosome-scale assemblies are not possible yet. However, these assemblies are sufficient for gene model comparisons across species (starting on Line 187). Is there a way to standardize gene prediction for cases where short reads may be all that is available?
Re-analysis of gene predictions with different tools may lead to varying estimates and can lead to erroneous orthology assignments (see https://doi.org/10.1111/jpy.12947, https://doi.org/10.1371/journal.pbio.3000862, and https://www.biorxiv.org/content/10.1101/2022.01.13.476251v1). Re-analysis of Rhopilema gene content using different tools increases gene predictions closer to the median gene count you've found."

Based on this commentary, we have added several sentences to clarify the problem of comparative analysis based on heterogeneous annotations. This point was explored in the section “The state of Medusozoa genomics: inner and derived knowledge” in relation to articles' conclusions about lineage-specific genes and increases/decreases in gene content. Moreover, this point was also recapitulated at the final part of the recommendations, reinforcing the problem of comparative analysis.

We made the following additions on line 314:

Recent evidence proved that the detection of lineage-specific genes, and other analyses relying on accurate annotation and orthology prediction, can be significantly biased by methodological artifacts [79–83]; several problems have been identified, such as low taxon sampling, heterogeneous gene predictions, and failure of detecting distant homology and fast-evolving orthologues. These considerations are highly relevant in Medusozoa, as comparisons are often made, by necessity, with distantly

Powered by Editorial Manager® and ProduXion Manager® from Aries Systems Corporation

related species (e.g. Anthozoa has been estimated to have diverged from Medusozoa around 800 million years ago [84]).
On line 460:
The latter suggestions (3-6) are mainly related to providing detailed methodologies of bioinformatic analyses. First, proper method and results descriptions can help to recover metadata and criteria usually not available in large sequence repositories. Second, comparative analyses depend upon standardization at different levels and significant sample sizes. The inclusion of species in downstream analyses is limited by data availability and proper description of previous analyses, custom software and results.
and on line 475:
The adoption of best practices in the Medusozoa genomics community will pave the way for major breakthroughs regarding understanding the genomic basis for several evolutionary innovations that arose within and in the stem lineage of Medusozoa. Similar advances were achieved with extensive taxon sampling at broader scales, where 25 novel core gene groups enriched in regulatory functions might be underlying the emergence of animals [109,110]. Medusozoa innovations have puzzled the community for decades [5,7,11,111] and include the origin of the medusa, the loss of polyp structures, the establishment of symbiosis, the blooming potential, and the evolution of an extremely potent venom. A deeper understanding of the genomic events driving these innovations will require accurate identifications of a number of key genomic features including (but not limited to) single copy orthologs, gene losses, lineage-specific genes, gene family expansions and non-coding regulatory sequences.
In relation to the question: "Is there a way to standardize gene prediction for cases where short reads may be all that is available?"
We are not aware of any pipeline specifically designed to standardize gene prediction for short-read assemblies. One solution would be to re-annotate and annotate all genomes by the same methodology. Another solution would be to use existing annotations and improve them by comparative analysis or by targeting specific gene families of interest. These considerations were added to “Prospects on genomic data and general resources" but not as part of the final recommendations on line 390.
An alternative solution for comprehensive comparative analyses is to (re)annotate all genomes with the same pipeline, a task that is laborious and time consuming. Some programs were designed for achieving this task simultaneously in many related species (e.g. [89,90]). Another alternative is to use specific software developed to improve genome annotations by leveraging data from multiple species (e.g. [91,92]) or targeting specific gene families [93,94]. Finally, differences in annotation due to methodological artifacts can be accommodated in comparative analysis if considered as a variable in the statistical tests (e.g. comparing tRNA genes in high and low quality avian genomes [95]).

"6. Regarding the recommendation for depositing intermediates into repositories (#3), is there one established for the community or are you referring to more general ones like Dryad, FigShare, Repbase, etc.? Providing an example genome project or two that shares these associated files might be helpful."

We were referring to general repositories. We have clarified this point in the section titled: "Deposit output results that were fundamental in any of the steps of the analysis" on line 446:

A Medusozoa-centric database with long-term maintenance is still lacking for the community (e.g. Mollusca clade [104]); but many open repositories can serve this purpose with low or no costs considering the size of the aforementioned outputs. There are open topic-centric repositories (e.g Dfam [105] for repetitive DNA), general repositories (e.g. FigShare, Zenodo; or even NCBI for annotation tracks) as well as personal or institutional ones. Many of the reviewed genomic projects already made use of these repositories but failed to deposit some of the outputs. A solution for this inconvenience is to update submissions or create novel ones (e.g. submit annotations to NCBI or ENA) to deposit the missing outputs.

"7. There can be cost associated with hosting these resources. Do you see that as a barrier to researchers providing this sort of data?"

Although repositories can be expensive, the intermediates we mentioned in

	recommendation #3 (gene and repetitive models and tracks) are frequently below 1gb. These file sizes can be easily accommodated by repositories with no cost at all. Therefore, we do not find cost to be a barrier for deposit. One possible barrier is that in general the submission process is cumbersome, something that might improve as new workflows are developed (as mentioned in the final conclusions of the manuscript).<br><br>"8. A recommendation that is provided earlier in the paper is the call for lineage-specific single copy ortholog sets (Line 228). Should this be re-stated in the final recommendations as well?"<br><br>The determination of a single copy ortholog set for Medusozoa would depend on the availability of gene annotations for several species, the completeness of these annotationes, or availability of sufficient information enabling re-annotation of these genomes. We believe this might not be possible yet in Medusozoa, therefore this topic was restated together with suggestion #5 (starting on line 480).<br><br>"Minor Comments:"<br>"9. Line 31-33: This sentence seems to be constructed of two thoughts but missing a connector between them."<br><br>This error was corrected as follows in the abstract:<br><br>Modern genomic DNA sequencing in this group started in 2010 with the publishing of the Hydra vulgaris genome "and" has experienced an exponential increase in the past three years.<br><br>"The following corrections were also done:"<br>"Line 98: … assembly statistics using the statswrapper.sh script …"<br>"Line 169: … [55], and the …"<br>"Line 315: Remove "of" between reusing and previously."<br>"Line 337: "reran" should be "rerun"."<br>"Line 389: Typo, "projects""<br><br>"10. Figures: The resolution of the figures provided made it difficult to review. Specifically Figure 3 was quite pixelated."<br><br>The figures are concordant with the journal's requirements. The low quality of figures might be due to compression before the journal sent them to the reviewers. High quality versions of each version can be downloaded from the link available next to the figures in the pdf or svg files in Supplementary file S9. Leaving aside, Figure 2 and 3 (now re-numbered as Figure 3 and 4) were corrected to improve visualization; font size was increased and graph legend was repositioned.
**Additional Information:**	
**Question**	**Response**
Are you submitting this manuscript to a special series or article collection?	No
**Experimental design and statistics**<br><br>Full details of the experimental design and statistical methods used should be given in the Methods section, as detailed in our Minimum Standards Reporting Checklist. Information essential to interpreting the data presented should be made available in the figure legends.	Yes

Powered by Editorial Manager® and ProduXion Manager® from Aries Systems Corporation

Have you included all the information requested in your manuscript?	
**Resources**<br><br>A description of all resources used, including antibodies, cell lines, animals and software tools, with enough information to allow them to be uniquely identified, should be included in the Methods section. Authors are strongly encouraged to cite Research Resource Identifiers (RRIDs) for antibodies, model organisms and tools, where possible.<br><br>Have you included the information requested as detailed in our Minimum Standards Reporting Checklist?	Yes
**Availability of data and materials**<br><br>All datasets and code on which the conclusions of the paper rely must be either included in your submission or deposited in publicly available repositories (where available and ethically appropriate), referencing such data using a unique identifier in the references and in the "Availability of Data and Materials" section of your manuscript.<br><br>Have you have met the above requirement as detailed in our Minimum Standards Reporting Checklist?	Yes

Manuscript Click here to access/download;Manuscript;Meduso_for_production.docx

The state of Medusozoa genomics: current evidence and future challenges

Santander, Mylena D. 1 ORCID 0000-0001-6750-4180

Maronna, Maximiliano M. 2 ORCID 0000-0002-2590-639X

Ryan, Joseph F. 3,4 ORCID 0000-0001-5478-0522

Andrade, Sónia CS. 1 ORCID 0000-0002-1302-5261

Affiliation

1. Departamento de Genética e Biologia Evolutiva, Instituto de Biociências, Universidade São Paulo, 277 Rua do Matão, Cidade Universitária, São Paulo, Brazil. ZIP CODE 05508-090.
2. Departamento de Zoologia, Instituto de Biociências, Universidade de São Paulo, São Paulo, 101 Rua do Matão Cidade Universitária, São Paulo, Brazil. ZIP CODE 05508-090.
3. Whitney Laboratory for Marine Bioscience, University of Florida, 9505 Ocean Shore Blvd, St. Augustine, Florida, 32080, USA
4. Department of Biology, University of Florida, 220 Bartram Hall, Gainesville, FL, 32611, USA

Corresponding authors: MDS mylena.santander@gmail.com, MMM maxmaronna@gmail.com

Abstract

Medusozoa is a widely distributed ancient lineage that harbors one-third of Cnidaria diversity divided into four classes. This clade is characterized by the succession of stages and modes of reproduction during metagenic lifecycles, and includes some of the most plastic body plans and life cycles among animals. The characterization of traditional genomic features, such as chromosome numbers and genome sizes, was rather overlooked in Medusozoa and many evolutionary questions still remain unanswered. Modern genomic DNA sequencing in this group started in 2010 with the publishing of the *Hydra vulgaris* genome and has experienced an exponential increase in the past three years. Therefore, an update of the state of Medusozoa genomics is warranted. We reviewed different sources of evidence, including cytogenetic records and high-throughput sequencing (HTS) projects. We focused on four main topics that would be relevant for the broad Cnidaria research community: 1) taxonomic coverage of genomic information; 2) continuity, quality and completeness of HTS datasets; 3) overview of the Medusozoa specific research questions approached with genomics; and 4) the accessibility of data and metadata. We highlight a lack of standardization in genomic projects and their reports, and reinforce a series of recommendations to enhance future collaborative research.

Keywords

Annotation, completeness, assembly, genome size, chromosome number, collaborative genomics

Background

Medusozoa subphylum includes nearly 4,055 species of invertebrates distributed in the classes Hydrozoa, Cubozoa, Staurozoa and Scyphozoa [1], which are found at all latitudes in almost all aquatic environments, from freshwater to marine, and from shallow to deep waters (Figure 1). Medusozoa species, together with the other cnidarians classes (i.e. Anthozoa and Endocnidozoa), harbor some of the most plastic life cycles and diverse body plans among animals [2], and represent one of its early diverging groups, with all major cnidarian lineages already present 500 million years ago [3].

The Medusozoa clade is characterized by different evolutionary novelties, such as the presence of linear mitochondria and the adult pelagic stage, also known as medusa or jellyfish [4–6]. Most medusozoan life-cycles are characterized by the succession of different stages, including a larval, benthic asexually reproducing polyp stage, and a sexually reproducing jellyfish stage [6,7]. This ancestral metagenic life-cycle pattern is highly plastic and in some groups has been extensively modified or even lost. For example, several lineages have lost the pelagic medusae or reduced it to a reproductive structure, or acquired colonial lifestyles during the benthic phase [8–10]. Other novel traits have emerged in Medusozoa such as complex body patterns, neuromuscular systems and sensory organs [11].

The history of Medusozoa genomics started with pioneer cytogenetics reports (e.g. [12,13]) and was followed later by genome size estimations [14,15]. Over the past 20 years, technological advances and cost reduction of genome-scale sequencing platforms have led to a steady increase in both number and diversity of sequenced genomes and transcriptomes [16,17]. Medusozoa is not an exception, as numerous genomic resources have become available for model and non-model species, especially in the last 3 years. This advance has enabled the study of the genetic basis of many Medusozoa novel traits (e.g. [18–22]. Previous reviews about cnidaria genomics have focused on the small number of species with sequenced genomes available at the time [11,23,24], on individual cnidarian lineages (i.e.

Myxozoa; [25]), or on specific topics such as toxins or evolution of novel traits [11,26]. Given the increasing amount of genomic information available, an update of the state of Medusozoa genomics is warranted.

Here, we provide a comprehensive review of the major advances in Medusozoa genomics over the past century. In order to shed light in the understanding of the genomic evolution of the group from high throughput sequencing (HTS) datasets, we report the main trends on the number and quality of available genome projects, taking into account basic information of sequencing datasets, genome assemblies, genome annotations, and accessibility of associated data and metadata.

Main text

1. Methods

We surveyed literature and databases for cytogenetic reports and genome size estimations. Our main source of genomic information and metadata was NCBI Genome (Assembly, Genomes, Nucleotide, Taxonomy and SRA; [27]). We retrieved data automatically using entrez-direct v.13.9 and NCBI datasets v. 12.12. For information not present in NCBI, we checked published articles for proper information collection, as well as personal repositories mentioned in the associated articles. Due to recent updates in taxonomic statuses, we modified the attribution of karyotypes, genome sizes and assemblies of several species (see main text and Supplementary Materials).

Because there have been subtle variations in metrics and statistics between most genome reports, we recalculated some statistics, allowing us to make meaningful comparisons. Briefly, we have generated the following: i) assembly statistics using the statswrapper.sh script from BBmap (v38.73; RRID:SCR_016965; [28]); ii) gene statistics from the original annotation files with AGAT (v0.6.0; [29]) and assessment of completeness of all assemblies using BUSCO (v5.0.0+galaxy0; RRID:SCR_015008; [30]) in genome mode and

Metaeuk software, using two Single Orthologs Databases (eukaryota_odb10, number of genes=255, number of species=70; metazoa_odb10, number of genes=954, number of species=65), available at the public Galaxy server [31,32].

Assembly quality was reported following the metric proposed by Earth Biogenome Project [33] (hereafter BGP-metric). This system avoids the use of ambiguous terminology for quality and uses a logarithmic scale where the first two numbers are the exponents of the N50 contig and scaffold (1: 0-99Kb; 2: 1-9.9Mb; 3: 10-99.9Mbp), and the third number corresponds to the level of chromosomal assembly (1: 90% DNA > assigned to chromosomes in silico; 2: chromosomal rearrangements validated by two data sources; 3: >80% DNA assigned to intra-species maps and experimental validation of all breakpoints; see [33]).

All graphs were generated using Python v.3 with ETE Toolkit v.3 [34], Matplotlib v3.3.1 [35] and Seaborn v.0.11 [36] and modified with Inkscape v.0.92 [37], to improve visualization (e.g. font size and spacing). The tree of figures 1 and 3 represent a simplified phylogenetic hypothesis obtained by combining phylogenies from previous studies (Scyphozoa [38], Medusozoa [5], Hydrozoa [39,40]), taking into account clades with high congruence and support values. Although the different phylogenetic hypotheses were mostly congruent, no single study nor molecular dataset comprised all the terminals discussed here. We manually compiled all genomic information and HTS metadata referenced in this review using a report model based on previous works and public databases such as NCBI (Supplementary file S1; [29,41,42]). The command line used for retrieving genetic information and metadata, for statistics calculation and the code used for graph generation are available at Supplementary file S2 and S3. All collected data was updated until May 1st 2021.

2. Genomic projects: whos and hows of Medusozoa

Chromosome numbers are known for 34 hydrozoan species and 5 scyphozoan, including 3 lineages of the *Aurelia aurita* sp. complex species ([12,13,21,43–51]; Supplementary file S4). Older chromosome descriptions for 25 species do not include

information about chromosome morphology and often lack photographic records or schematic representations [12,13,43–47].

Genome size, a fundamental feature in genome sequencing project, has been experimentally estimated by Flow Cytometry or Feulgen Densitometry techniques, for 24 medusozoan species (Scyphozoa: 7spp.; Cubozoa: 1spp.; Hydrozoa: 16 spp.; Supplementary file S4). Genome sizes are highly variable ranging from 254 Megabases (Mbp) to 3,481.68 Mbp in *Sanderia malayensis* (Scyphozoa) and in *Agalma elegans* (Hydrozoa), respectively [15]. Moreover, an additional 12 genome size estimates are available when considering k-mer-based computational assessments, increasing the number of species with genome size information to 30, and including two cubozoans (913-2,673Mbp) and one staurozoan (230 Mbp) (Supplementary file S1; Supplementary file S4). These estimates are considered less accurate, especially for genomes with high heterozygosity, high repetitive content and large genome size [52]. In fact, kmer based and experimental estimations from the same species differed by 13-33%.

A total of 34 HTS projects were identified. Of these, 32 had sequencing reads accessible through the NCBI-SRA database but not all of them were associated with a genome assembly (Table 1; Supplementary file S1). The taxonomic coverage of the assemblies encompassed 7 of the 13 Medusozoa orders, and represented at least one species per class (Figure 2): 28 assemblies were accessible for 21 species, representing 0.5 % of Medusozoa (Figure 2; Table 1; Supplementary file S1). Of these 21 species, 12 were Scyphozoa, 4 were Hydrozoa, 4 were Cubozoa, and one was Staurozoa. Scyphozoa had the highest number of sequenced families (4 of 22), of which Pelagiidae contained the highest number of sequenced species so far (5 spp.), followed by Ulmaridae, Rhizostomatidae and Cassiopeiidae with 2 spp. each (Figure 2), all belonging to subclass Discomedusae (none from Coronamedusae). The remaining assemblies represent three of the eight Cubozoa families and three of 135 Hydrozoan families (Figure 2). In addition to the small fraction of family representation in the

hydrozoan genomes, the underrepresentation of Leptothecata is particularly unfavorable as it harbors more than half of Medusozoa species (2,059 sp; [1]).

------------TABLE 1 SHOULD BE LOCATED HERE------------

Much of the assembly effort is biased towards a small number of species. For example, three species of Hydrozoa and Scyphozoa presented two assemblies each, of which *Hydra viridissima* and *Rhopilema esculentum* were sequenced twice independently, meanwhile *Chrysoaora quinquecirrha* presents two versions of the same assembly. Moreover, three assemblies were available for two different strains of *Hydra vulgaris* (former *Hydra magnipapillata*), one of them published as an update of the reference genome called Hydra 2.0. In *Aurelia,* the genomes of three different lineages were sequenced and assembled: Baltic sea, Roscoff and *Aurelia* sp1. strains [19,20]. Based on a recent taxonomic update of this genus [53], locality and genetic information described in the original articles [19,20], we decided to refer to these genomic datasets as: Baltic sea strain = *Aurelia aurita;* Roscoff strain and *Aurelia* sp1. strains = *Aurelia coerulea*.

Most of the assemblies were deposited in NCBI Assembly database, one was only found in a journal-specific database (i.e. GigaDB [54]), one assembly was only in a personal repository (Google Drive) and one in the National Human Genome Research Institute site [55]. Some assemblies were additionally deposited in Institute-centered repositories such as OIST Marine Genomics Unit [56], and the Marine Invertebrate Models Database (MARIMBA, [57]). A significant portion of the publicly available assemblies (total of 8, ~30%) are not yet associated with a formal publication and belong to the IRIDIAN GENOMES project [58]. The most frequent sequencing technology was Illumina (26 assemblies, ~93%), but leaving aside unpublished ones, most works include a combination of different sequencing techniques, library sizes and platforms (i.e Sanger, 454, Illumina, long reads, linked-reads and Hi-C sequencing; Supplementary file S1).

Almost all medusozoan genome assemblies were at draft contig or scaffold level, with one exception, *Rhopilema esculentum*, where chromosome-level scale assembly was reported [59]. The total length, contig and scaffold number, N50, and GC% varied across species and classes (Figure 3A; references in Supplementary file S5). The assembly continuity and quality was higher in Scyphozoa than in the other classes, as observed by the distribution of contig and scaffold N50 (Figure 3A) and the BGP-metric for assembly quality (Figure 3A). In general, they are fragmented (75%), and have contig N50 of less than 40 Kbp (Figure 3A; BGP-metric values of 0.0.0, 0.1.0 and 0.2.0). Staurozoa, Cubozoa and Scyphozoa assemblies have similar percentages of base composition, around 35% to 43% GC. Consistent with previous reports [60], Hydrozoa genomes have a higher dispersion of GC%, with the GC values of five assemblies below 35%.

In relation to gene content (Figure 3B), 17 genomes were annotated using at least one source of information (Supplementary file S1) and their total number of genes or total number of protein-coding genes were reported. Further description of coding information was variable among works and as more detailed information was considered, the number of genomes with reported information decreased. Annotation tracks and gene models were available for only 11 of the 17 datasets. Recalculations of gene features together with the information recovered from original articles, allowed us to analyze the distribution of 5 different features in 15 genomes of Scyphozoa, Hydrozoa and Cubozoa (Figure 3B; Box): Number of genes (n=15), Mean exons per cds (n=10), Mean gene length (n=11), Mean exon length (n=11), Mean intron length (n=12). For three species, *Cassiopea xamachana* (Scyphozoa; 31,459), *Alatina alata* (Cubozoa; 66,156) and *Calvadosia cruxmelitensis* (Staurozoa; 26,258), the available information was restricted to the number of predicted genes. Some small inconsistencies were detected between original data reported in some papers and our recalculations (Table S5-6), and others between data reported in the main text and supplementary materials of some papers.

The determination of repetitive DNA has been an integral step before gene annotation in most genomic projects. Frequently, repeat diversity was not properly reported and the degree of

detail also varied between articles: e.g. some published works only referred to the most abundant class of repetitive DNA, meanwhile others described only results at class or family level. Repetitive libraries —consensus sequences representing repeat families— were not properly saved in repositories with the exception of two independent articles, and RepeatMasker results were reported in 4 articles (one reporting only classified repeats). Total repetitive length of 12 species for which coding information was also available is presented in Figure 3B and discussed in Box.

The degree of completeness of these datasets also varied substantially, as estimated by BUSCO (metazoa_odb10 and eukaryota_odb10; Figure 4). While all Eukaryota genes were present in at least one assembly (Supplementary file S5, Supplementary file S6), the level of absence and fragmentation of Metazoa genes was higher (Figure 4. Supplementary file S5). Seven Metazoa genes were absent in all assemblies and 17 were absent in more than 20% of them (Figure 4, indicated in red). Some Metazoa BUSCO genes were absent in lineages with the higher number of assemblies, such as Scyphozoa and Hydrozoa (Figure 4. indicated in yellow rectangles; Supplementary file S5). This condition was suggested by [20], after detecting the absence of 14 genes in 5 species (version metazoa_o9db), 3 of which coincided with the genes detected as absent here (Orthodb IDs: 460044at33208, 601886at33208, 114954at33208), one of which (445034at33208) that has a patchy distribution in Medusozoa and 9 of which were removed in later versions of the database (Figure 4 in bold).

Moreover, 27 genes were simultaneously recovered as undetectable or fragmented in more than 80% of the assemblies (Supplementary file S5 table S7). Based on BUSCO completeness assessment with metazoa_o10db, 13 assemblies present 90-95% of genes (fragmented+complete), while only one assembly includes over 90% of complete genes; the remaining 15 assemblies present between 57-87% of genes (complete+fragmented) or 16-77% complete genes. While the Metazoa database might include genes that are absent, fragmented, or have non-conventional features in all medusozoa species, the utility of the Eukaryota database in the completeness assessment is limited by its low number of genes.

Until more specific databases are developed, the combination of both BUSCO databases should be used taking into account their limitations.

Differences in sequencing strategy and platforms are expected to be linked with assembly quality, both in terms of continuity and completeness. For example, hybrid sequencing plus optical maps and combined evidence-based annotation should generate better results than a short-read sequencing and single-evidence annotation [61,62]. Although this general trend was observed in this review, with most Illumina-only datasets showing lower BGP-metric (Figure 3) and lower completeness (Figure 4), it is not a granted condition. Some punctual cases can exemplify biological and methodological issues that impose limitations to genome sequencing and assembly: e.g. the difficulty in obtaining chromosome-scale assemblies despite small genome sizes and combined sequencing strategies (Hi-C + short reads+ long reads) [63,64] or the difficulty in extracting high-molecular-weight DNA [20]. Because of the heterogeneity of Medusozoa genomic projects in terms of time periods, objectives, methods and resources, a proper quantitative analysis of the relationship between methods and outcome quality would not be feasible, and we prefer to refer to articles specialized in assessing methods (e.g. [61,62]).

3. The state of Medusozoa genomics: inner and derived knowledge

The first glimpse of the Medusozoa genomic organization was obtained by cytogenetic studies [12,13,21,43–51], but in contrast to other animals, the available information is still sparse. Many cytogenetic questions essential to the understanding of genome evolution are unanswered in Medusozoa, either at species or population scale, including the distribution of the chromosome number (2n), fundamental number of chromosome arms (FN), genome size, ploidy level, heterochromatin content. These are questions that have gained renewed interest since the arrival of the genomic era.

Regarding the phylogenetic distribution of the chromosome number, no inferences can yet be made on the sparse available information, apart from the presence of some

chromosome variation throughout Medusozoa. A special case was reported in *Hydra* where, according to recent descriptions, many species shared a 2n=30 karyotype with metacentric or submetacentric chromosomes ([51]; Supplementary file S4). This suggests that the 2n=30 karyotype could be widely distributed in the genus and even in other Hydrozoa groups, since it was also described for one species of Hydrocorynidae, Hydractiniidae, Campanulariidae, Bougainvilliidae, and Clytiidae, and 3 Eirenidae (Supplementary file S4; references therein). Interestingly, in Anthozoa, a few sea anemones and several scleractinian corals have karyotypes between 2n=28 and 2n=30 [65–67]. Nevertheless, a higher sampling effort should be conducted in order to test the extent of this apparent karyotype stability.

Scyphozoa genomes tend to be smaller (~250 to ~700 Mbp) than those of Hydrozoa, which encompass a larger range (~380 to ~3,500 Mbp) (Figure 2; Supplementary file S4, references therein), but due to the scarcity of estimations that represent around 1% of the subphylum, these ranges should be considered preliminary. The evolution of eukaryotic genome size is a long-standing question that has been called the “C-value Enigma” [41]. This name stems from the difficulty elucidating the evolutionary forces (e.g. drift and natural selection) that have given rise and serve to maintain variations in genome size, the mechanisms of genome size change, and the consequences of these variations at an organismal level [41]. Several conflicting hypotheses have been postulated to explain this puzzle with most having experimental support in some but not all lineages (reviewed in [68]). The molecular basis of these variations in Medusozoa have only been studied in detail for *Hydra* [69] and for *S. malayensis* [63]; their trends have been related to repetitive DNA and gene length respectively (Box). Meanwhile, the ecological and historical factors underlying genome size diversity and its extent in Medusozoa, are topics that remain to be elucidated.

Box. Genome content

Gene content and length: it is straightforward to imagine that the evolution of these two characteristics have potential impacts in macroevolution of organisms. The distribution of gene number in Medusozoa (Figure 3B) ranged from 17,219 in the Scyphozoan *Rhopilema esculentum* [59] to 66,156 in the Cubozoan *Alatina alata* [22], but most species of all classes have gene counts near the median (26,258), which is higher than the range (18,943 ± 451.82) described for animals [41]. The upper limit described in the highly fragmented *A. alata* genome deviates from the observed in *Morbakka virulenta (*24,278 genes), the only other sequenced Cubomedusae [20,22]. Species with varying genome sizes of Hydrozoa, Scyphozoa and *M. virulenta* (Cubozoa) had similar mean CDS lengths (1,414, 1,214, 1,387 base pairs), mean numbers of exons per gene (5, 6, 5.4), mean exon lengths (306, 293, 432 bp), but had different gene lengths (9,530, 7,855 and 21,444 bp respectively) due to the presence of longer introns in Hydrozoa and Cubozoa when compared to Scyphozoa (Hydrozoa: 1,600; Cubozoa: 3,705 vs. 1,146 bp in Scyphozoa). This is best exemplified in the genome of the scyphozoan *S. malayensis*, which has the smallest cnidarian genome reported to date [63], and has also the smallest introns of any sequenced medusozoan genome (Figure 3B. yellow arrowhead). Nevertheless, these ranges are rough estimates and sometimes heterogeneous, e.g. resulting from different filtering parameters, and their implications should be tested as new assemblies and annotations become available.

Repetitive content: repetitive DNA represents a significant part of eukaryotic genomes and is highly diverse, composed by different kinds of transposable elements (TEs), tandem repeats and multigene families (e.g. rRNA and tRNA). Many of these sequences, especially TEs and satellite DNA, were initially considered as an expendable sector of the genome, although their impact on genomic evolution has since been recognized (reviewed in [70]). For example, fusion between TEs and host genes have occurred multiple times in vertebrates and have contributed to the evolution of novel features [71]. Likewise, TEs and other repetitive DNA have been associated with genomic rearrangements and changes in

DNA content (e.g. [69,70]). The *Hydra* genus, which has been more extensively studied from this point of view, has experienced a rapid genomic evolutionary rate and presents a 3-fold genome size increase resulting from the amplification of a single LINE family [69]. Moreover, *Hydra* genomes include an over-representation of transposase-related domains [72]. It is interesting to note that many of the Medusozoa species studied so far have relatively small genomes but unusually high proportions of repetitive DNA [20,63,73,74]. Nevertheless, the lack of standardization in the description of its diversity, and the discrepancy in the degree of detail in which these have been described, limits the potential to make inferences. Repetitive DNA is a complex study subject, limited by assembly continuity and annotation effort, but restricting genomic studies to the "functional" part of the genome (sensu [75]) may lead us to a narrowed view of the Medusozoa genome evolution.

Modern Medusozoa genomics formally started with the sequencing and publication of *Hydra vulgaris* genome that in cnidaria was only preceded by *Nematostella vectensis* [65,76]. *Hydra vulgaris* is one of the earliest models in biology, mainly used for the study of development, regeneration, and more recently, of aging (reviewed in [77,78]). The study of these two early genomes was fundamental for the reconstruction of a more complex ancient eumetazoan genome than first suggested by the comparison of vertebrates and insects [16,23,65,76].

Unlike most other medusozoan species, *Hydra* lives in freshwater, lacks a medusa and has a genome that has experienced a very rapid rate of evolution [21]. It therefore is not the ideal species for reconstructing historical nodes on the Medusozoa tree of life. As such, more recent medusozoa genomes have led to important updates in our understanding of Medusozoa-relevant research topics, including phylogenetic reconstructions, the genetic basis of the medusae, the evolution of symbiosis, toxin characterization, Homeobox gene evolution, to name a few examples (Table 1). Nevertheless, Medusozoa genomes include

thousands of single-copy genes and repetitive elements; however, only a very limited number of them have been analyzed in detail.

The complex nature of Medusozoa venom has been investigated by a number of transcriptomic, proteomic and genomic studies (reviewed in [26]). Several putative toxin genes and domains have been identified, covering a significant part of the wide range of known toxins [20,22,59,73]. In Scyphozoa, toxin-like genes were often recovered as multicopy sets [20,59]**.** Moreover, in *R. esculentum* toxin-like genes were also tandemly arranged and several of them were located nearby in chromosome 7, suggesting that the observed organization might influence toxin co-expression[59]. Minicollagens, which are major components of nematocysts, also had a clustered organization and a pattern of co-expression in *Aurelia* [20]. These examples add to various clustered genes described in Cubozoa, Hydrozoa and Anthozoa, and would indicate that gene clustering and operon-like expression of toxin genes is widespread in Cnidaria ([20] and references therein).

The determination of lineage specific genes and increases and decreases of gene content is one of the recurrent questions found in Medusozoa genomic studies (e.g. [20,21]), and it has been conducted using different methodologies and sets of species. Recent evidence proved that the detection of lineage-specific genes, and other analyses relying on accurate annotation and orthology prediction, can be significantly biased by methodological artifacts [79–83]; several problems have been identified, such as low taxon sampling, heterogeneous gene predictions, and failure of detecting distant homology and fast-evolving orthologues. These considerations are highly relevant in Medusozoa, as comparisons are often made, by necessity, with distantly related species (e.g. Anthozoa has been estimated to have diverged from Medusozoa around 800 million years ago [84]). In Cnidaria, It has been estimated the most elevated rates of loss in the hydrozoan branch leading to *Clytia hemisphaerica* and *Hydra* [21,76], followed by slightly lower rates of gene loss in Scyphozoa and substantially lower rates in Anthozoa [19]. Gene families that have experienced expansion and contraction have been studied in relation to complex life cycle patterns [19,21], simplification of the body plan

[72,76], the evolution of symbiosis [72], among others (table 1). Expression patterns of identified taxonomically restricted medusozoan genes have been mainly studied in the context of life cycle stages (e.g. [20,21]).

The complex life cycle of Medusozoa has resulted from the combination of both ancestral and novel features. *Aurelia*, *Morbakka virulenta* and *Clytia hemisphaerica* have significantly different patterns of gene expression across stages and during transitions [19–21]. Differentially expressed genes include many conserved ancestral families of transcription factors [19–21]; there is also a considerable amount of the putative lineage-restricted genes that show differential expression in the adult stages [20,21]. A few of these "novel" medusozoan genes have been described, such as novel myosin-tail proteins that are absent from Anthozoa and represent markers of the medusae striated muscles [20]. It was suggested that the evolution of the Medusozoa complex life cycle would therefore have involved the rewiring of regulatory pathways of ancestral genes and the contribution of new ones [19–21]. As such, the body plan and life cycle simplifications observed in *Clytia* and *Hydra*, respectively, would be the result of loss of transcription factors involved in their development [21]. Finally, the significance of many of the putative Medusozoa and species-specific genes remain to be elucidated.

On the other hand, synteny was also analyzed several times, including species of Hydrozoa, Cubozoa and Scyphozoa, and were carried on at different scales depending on assembly continuity (i.e. microsynteny and macrosynteny), and often comparing the focus species to species from sister clade Anthozoa [19–21,67,76]. High synteny conservation was found within Anthozoa (*N. vectensis* vs. *Scolanthus callimorphus* [65–67]) and within Hydrozoa (*H. vulgaris* vs. *C. hemisphaerica;* [21]). Meanwhile, conservation of synteny at a lesser degree was also observed between Anthozoa and Scyphozoa (*N. vectensis* vs. *R. esculentum*; *N. vectensis* vs. *Aurelia* strains; [19,20,67]) and only a few shared syntenic blocks between Hydozoa and Anthozoa (*H. vulgaris* vs. *N. vectensis;* [21,67,76]), Hydrozoa and Scyphozoa (*H. vulgaris* vs. *Aurelia aurita;* [19]) and Scyphozoa and Cubozoa (*A. aurita vs. M.*

virulenta; [20]). It is particularly interesting to note that *H. vulgaris, N. vectensis* and *S. callimorphus* present 2n=30, but shared fewer syntenic blocks than either of the two anthozoans with *R. esculentum*, which has a different karyotype (2n=22) [67] (non peer-reviewed). These results suggest that there is evidence for the conservation of an ancient genome architecture in Anthozoa and Scyphozoa, but less conservation in Hydrozoa and Cubozoa, coincident with a more rapid rate of genome reorganization in the last two classes [21,67].

4. Prospects on genomic data and general resources

The increasing amount of genomic information available for diverse organisms has enabled statistical inferences of trends in eukaryotic genomic evolution. Examples of such studies are available at small and large phylogenetic scales and have enabled evolutionary analyses of the distribution of gene numbers, gene features (e.g. intron size), and repetitive content (e.g. [41]). Nevertheless, the power of eukaryotic genomic comparative analyses is hindered by a lack of data and metadata standardization [41,85], which is especially evident in Medusozoa.

There is much to learn from decades-old references of cytogenetic studies, but some studies, especially older ones, lack complete material and methods (e.g. pretreatment, references, designs and photographs; general metadata as locality, taxonomic identification) and therefore should be considered carefully in a comparative framework (e.g. [86]).

Similar problems can be expected in relation to genomic data, as metadata is often not specified in great detail. We analyzed hundreds of fields including genetic information and metadata (methods, metrics and registry codes; table Supplementary file S1), of which no dataset presents most of them, whatever the area or section (e.g. processing area, section trimming). This could be a future problem because reusing previously published datasets is becoming routine, and tracking of information (BioProjects, Biosamples, methodologies, filtering parameters, etc.) would be misleading [85,87].

Descriptions of bioinformatic methods in genome studies are often even less comprehensive than database metadata. For example, we identified at least three independent projects, each of which applied different criteria for gene model filtering, and another three articles applied slightly different criteria for repeat library filtering (Supplementary file S1). Although differences at this stage can seem small on the surface, they can result in hard-to-detect biases downstream that can lead to flawed biological conclusions. For example, resistance genes have been underestimated in some flowering plant genomes due to inconsistencies of genome annotation stemming from differences in repeat masking [88]. Likewise, in the current review, we identify discrepancies in BUSCO genome completeness comparisons that are caused by differences in database versions, which are frequently unspecified in the associated articles.

An alternative solution for comprehensive comparative analyses is to (re)annotate all genomes with the same pipeline, a task that is laborious and time consuming. Some programs were designed for achieving this task simultaneously in many related species (e.g. [89,90]). Another alternative is to use specific software developed to improve genome annotations by leveraging data from multiple species (e.g. [91,92]) or targeting specific gene families [93,94]. Finally, differences in annotation due to methodological artifacts can be accommodated in comparative analysis if considered as a variable in the statistical tests (e.g. comparing tRNA genes in high and low quality avian genomes [95]).

The submission of raw sequencing data and fundamental metadata to the NCBI-SRA or EMBL-ENA remains a vital step in ensuring the usability and transparency of genome data [96,97]. Also, project centric repositories serve to store assemblies and associated datasets, and enable comparative studies by basic tools. Taxon-restricted databases including cnidarian data have been employed in the past, but these are often not maintained due to lack of upkeep funding and other factors (e.g, [98,99]). In addition, submission to the large databases like SRA and GenBank can lead to the automatic detection of specific issues such as

contamination or annotation errors that might otherwise not be detected. For these reasons, the large general databases should remain the primary repositories for sequence and metadata [100]. Nevertheless, this is not always the case. For example, the assembly with the highest continuity as estimated by the BGP-metric, corresponding to *R. esculentum* [59], is only found in a journal-specific database and lacks a stable identifier (e.g. NCBI accession). A similar situation is observed for one of *Hydra vulgaris* assemblies (Hydra 2.0) which is only found in a project-specific database [55].

There is a growing number of community-driven guidelines, standards, databases and resources based on the Findable, Accessible, Interoperable and Reusable principles (FAIR principles) for digital research outputs [100]. Furthermore, global initiatives of large-scale genome sequencing included in Earth Biogenome Project have adopted a set of standardized protocols for the different stages of the genome projects, such as specimen collection, DNA extraction, sequencing, assembly and annotation methods, and reporting, in order to generate datasets that could "be useful to the broadest possible scientific community" [33]. Standards should be also implemented by independent research groups publishing genomes. The main goal of standardization is to promote evaluation, discovery, and reuse of genomic information, providing long term benefits for science.

The following are suggestions to enhance genome projects and outcomes, and to promote open and collaborative research. These suggestions can be broadly applied to any genome project and are in line with those proposed by many initiatives and consortia (e.g. [33,100,101]). Nevertheless, it is worth reinforcing and discussing them in the context of this review since genome projects are more and more often being initiated in research laboratories that have historically been more focused on other aspects of medusozoan biology and may not be as familiar with these general practices:

1. *Deposit all data and metadata in public specialized databases (e.g. NCBI), at least once associated articles are accepted for publication. Provide comprehensive metadata, including*

those not considered as priority for the aforementioned project. Frequently, data and metadata that are described in the original articles or deposited in repositories are not submitted to public databases. Tracking information from multiple sources is time consuming and prone to error. Databases and repositories enable the improvement of metadata after the initial releases, by the addition of new or corrected information (e.g. publication information) from the authors. We believe that this kind of data curation would improve the state of Medusozoa genomics not only by enabling downstream analysis after the publication, but also enabling the detection of methodological options (e.g. tissue selection; sequencing technology) that would improve the quality of the results.

2. *Consider providing standardized genome statistics in an easily accessible format (e.g. Supplementary file S1 presented here)*. *Alternatively, use specialized tools that standardize reports for multiple samples and datasets* (e.g. [42,102,103]). This will facilitate meta-analyses, prompt new genome studies to make accurate comparisons to previously published studies, and prevent the propagation of erroneous information.

3. *Deposit output results that were fundamental in any of the steps of the analysis (e.g. gene models, repetitive libraries and annotation tracks).* A Medusozoa-centric database with long-term maintenance is still lacking for the community (e.g. Mollusca clade [104]); but many open repositories can serve this purpose with low or no costs considering the size of the aforementioned outputs. There are open topic-centric repositories (e.g Dfam [105] for repetitive DNA), general repositories (e.g. FigShare, Zenodo; or even NCBI for annotation tracks) as well as personal or institutional ones. Many of the reviewed genomic projects already made use of these repositories but failed to deposit some of the outputs. A solution for this inconvenience is to update submissions or create novel ones (e.g. submit annotations to NCBI or ENA) to deposit the missing outputs.

4. Inform as much as possible if a dataset was edited (e.g. removal of exogenous DNA; gene and repetitive sequence filtering criteria).

5. Use and clearly identify software, database versions and references in all instances (e.g. RRID, BUSCO version and repetitive database version).

6. Deposit command lines and scripts used to handle data (from reads to full annotation).

The latter suggestions (3-6) are mainly related to providing detailed methodologies of bioinformatic analyses. First, proper method and results descriptions can help to recover metadata and criteria usually not available in large sequence repositories. Second, comparative analyses depend upon standardization at different levels and significant sample sizes. The inclusion of species in downstream analyses is limited by data availability and proper description of previous analyses, custom software and results.

7. Engage in community-driven conversations about standards, guidelines and species priorities. There are a number of taxon-specific meetings that would be appropriate venues to engage in these conversations including the International Conference on Coelenterate Biology (~decennial; [106]), the International Jellyfish Blooms Symposium (~triennial), Cnidofest (~biennial; [107]), Tutzing workshop (~biennial; [108]), and Cnidofest zoom seminar series. In addition, satellite meetings at larger annual meetings (e.g. the Society for Integrative and Comparative Biology (SICB) or the Global Invertebrate Genomics Alliance (GIGA [101])) could provide appropriate venues to facilitate discussions on how the community can best move forward as more and more genomic data come online.

The adoption of best practices in the Medusozoa genomics community will pave the way for major breakthroughs regarding understanding the genomic basis for several evolutionary innovations that arose within and in the stem lineage of Medusozoa. Similar advances were achieved with extensive taxon sampling at broader scales, where 25 novel core gene groups enriched in regulatory functions might be underlying the emergence of animals [109,110]. Medusozoa innovations have puzzled the community for decades [5,7,11,111] and include the origin of the medusa, the loss of polyp structures, the establishment of symbiosis, the blooming potential, and the evolution of an extremely potent

venom. A deeper understanding of the genomic events driving these innovations will require accurate identifications of a number of key genomic features including (but not limited to) single copy orthologs, gene losses, lineage-specific genes, gene family expansions and non-coding regulatory sequences.

Conclusions

The pace of genomic development in Medusozoa is far more rapid than more traditional disciplines such as cytogenetics, where gaps still remain. As the effect of chromosome structural variants in evolution is increasingly tested and recognized, it is expected that these disciplines will gain a revived interest as has been seen in other animal groups [112]. In spite of the great advances in Medusozoa genomics, we found a general lack of standardization in methodologies and genome reports across independent sequencing projects. Efforts to incorporate standards would benefit future studies and could promote the identification of hitherto undiscovered evolutionary patterns.

It is safe to anticipate that standardization will become increasingly easier as chromosome-level assemblies become more commonplace and as new integrated workflows of data reporting and submission are developed (e.g. [113]). It will be possible to perform standardized annotation and analyses in order to identify patterns in medusozoa genome evolution.

The distribution of genetic and genomic information presented significant taxonomic gaps in Medusozoa. It is a reasonable scenario since genomic sequencing data is accumulating in many medusozoan lineages. Even so, some of the most species-rich clades with a diverse array of phenotypic and ecological traits have not yet had their genomes sequenced (e.g. Scyphozoa: Coronamedusae, Hydrozoa: Macrocolonia). These, and other, heretofore genomically underexplored lineages provide golden opportunities from which to make major contributions to understanding the evolution of Medusozoa genomes and would be a wonderful contribution to the rest of the Medusozoa research community. Defining

candidate species for sequencing can avoid unnecessary doubled efforts. Different international projects recognized this situation and proposed a set of criteria for prioritizing species at other scales, such as the GIGA ([101]).

Conversations about how best to promote such efforts and best practices for medusozoan genomics will help move the field forward. Such conversations could lead to new standards and potentially a powerful cnidarian genomics database. This latter goal would be most effective if accompanied by a strong alliance that spans the growing cnidarian genomics community.

Data availability

All collected information, outputs and scripts supporting new results are available in the supplementary files S1-S9 in Figshare [114]. All genomic resources from previous articles and projects are publicly available and their sources are referenced in Supplementary file S4 Table S3. A copy of table S1 is available [114] and can be updated upon the original author's request.

Competing interests

The authors declare that they have no competing interests

Author's contributions

MDS collected the information, ran the analysis, conceived the study and drafted the manuscript; MMM collected the information, conceived the study, drafted and reviewed the manuscript; JR drafted and reviewed the manuscript. SCSA conceived the study, drafted and reviewed the manuscript. All authors gave final approval for publication.

Funding

This work was supported by Coordenação de Aperfeiçoamento de Pessoal de Nível Superior 88882.377420/2019-01 to MDS, Fundação de Amparo à Pesquisa do Estado de São Paulo FAPESP 2016/04560-9 to MMM, FAPESP 2015/20139-9 to SCSA.

Acknowledgments

We thank Analia Jaque for the jellyfish drawings of Figure 2, Juan M. Ferro for valuable comments on this review and Jonathan W. Lawley for the taxonomy update on the *Aurelia* samples.

References

1. World Register of Marine Species. Cnidaria. http://www.marinespecies.org/aphia.php?p=taxdetails&id=126. Accessed 24 Nov 2021.

2. Bosch TCG, Adamska M, Augustin R, Domazet-Loso T, Foret S, Fraune S, et al.. How do environmental factors influence life cycles and development? An experimental framework for early-diverging metazoans. *BioEssays*. 2014; doi: 10.1002/bies.201400065.

3. Cartwright P, Collins AG. Fossils and phylogenies: integrating multiple lines of evidence to investigate the origin of early major metazoan lineages. *Integr Comp Biol*. 2007; doi: 10/bzhzc2.

4. Bridge D, Cunningham CW, Schierwater B, DeSalle R, Buss LW. Class-level relationships in the phylum Cnidaria: evidence from mitochondrial genome structure. *Proc Natl Acad Sci USA*. 1992; doi: 10/dxgw77.

5. Kayal E, Bentlage B, Sabrina Pankey M, Ohdera AH, Medina M, Plachetzki DC, et al.. Phylogenomics provides a robust topology of the major cnidarian lineages and insights on the origins of key organismal traits. *BMC Evol Biol*. 2018; doi: 10.1186/s12862-018-1142-0.

6. Marques AC, Collins AG. Cladistic analysis of Medusozoa and cnidarian evolution. *Invertebr Biol*. 2004; doi: 10.1111/j.1744-7410.2004.tb00139.x.

7. Collins AG. Phylogeny of Medusozoa and the evolution of cnidarian life cycles. *J Evol Biol*. 2002; doi: 10.1046/j.1420-9101.2002.00403.x.

8. Boero F, Boero F Bouillon, J. Zoogeography and life cycle patterns of Mediterranean hydromedusae (Cnidaria). *Biol J Linn Soc*. 1993; doi: 10/cgvp43.

9. Da Silveira FL, Morandini AC. *Nausithoe aurea* n. sp.(Scyphozoa: Coronatae: Nausithoidae), a species with two pathways of reproduction after strobilation: sexual and

asexual. *Contrib Zool*. 1997;66:235–46.

10. Straehler-Pohl I, Jarms G. Morphology and life cycle of *Carybdea morandinii*, sp. nov.(Cnidaria), a cubozoan with zooxanthellae and peculiar polyp anatomy. *Zootaxa*. 2011;2755:36–56.

11. Forêt S, Knack B, Houliston E, Momose T, Manuel M, Quéinnec E, et al.. New tricks with old genes: the genetic bases of novel cnidarian traits. *Trends Genet*. 2010; doi: 10/dd74gw.

12. Harvey EB. A review of the chromosome numbers in the Metazoa. Part I. *J Morphol*. 1916; doi: 10.1002/jmor.1050280102.

13. Makino S. An atlas of the chromosome numbers in animals. 2nd ed. Ames: The Iowa State College Press; 1951.

14. Goldberg RB, Crain WR, Ruderman JV, Moore GP, Buckley TR, Higgins RC, et al.. DNA sequence organization in the genomes of five marine invertebrates. *Chromosoma*. 1975; doi: 10.1007/BF00284817.

15. Adachi K, Miyake H, Kuramochi T, Mizusawa K, Okumura S. Genome size distribution in phylum Cnidaria. *Fish Sci*. 2017; doi: 10/ggbqnf.

16. Dunn CW, Ryan JF. The evolution of animal genomes. *Curr Opin Genet Dev*. 2015; doi: 10/gfkjbf.

17. Goodwin S, McPherson JD, McCombie WR. Coming of age: ten years of next-generation sequencing technologies. *Nat Rev Genet*. 2016; doi: 10.1038/nrg.2016.49.

18. Lewis Ames C, Ryan JF, Bely AE, Cartwright P, Collins AG. A new transcriptome and transcriptome profiling of adult and larval tissue in the box jellyfish *Alatina alata*: an emerging model for studying venom, vision and sex. *BMC Genomics*. 2016; doi: 10.1186/s12864-016-2944-3.

19. Gold DA, Katsuki T, Li Y, Yan X, Regulski M, Ibberson D, et al.. The genome of the jellyfish Aurelia and the evolution of animal complexity. *Nat Ecol Evol*. 2019; doi: 10/gfkwp4.

20. Khalturin K, Shinzato C, Khalturina M, Hamada M, Fujie M, Koyanagi R, et al.. Medusozoan genomes inform the evolution of the jellyfish body plan. *Nat Ecol Evol*. 2019; doi: 10/gfzg9m.

21. Leclère L, Horin C, Chevalier S, Lapébie P, Dru P, Peron S, et al.. The genome of the jellyfish *Clytia hemisphaerica* and the evolution of the cnidarian life-cycle. *Nat Ecol Evol*. 2019; doi: 10/gfwr3v.

22. Ohdera A, Ames CL, Dikow RB, Kayal E, Chiodin M, Busby B, et al.. Box, stalked, and upside-down? Draft genomes from diverse jellyfish (Cnidaria, Acraspeda) lineages: *Alatina alata* (Cubozoa), *Calvadosia cruxmelitensis* (Staurozoa), and *Cassiopea xamachana* (Scyphozoa). *GigaScience*. 2019; doi: 10.1093/gigascience/giz069.

23. Steele RE, David CN, Technau U. A genomic view of 500 million years of cnidarian evolution. *Trends Genet TIG*. 2011; doi: 10/b53t8x.

24. Technau U, Schwaiger M. Recent advances in genomics and transcriptomics of cnidarians. *Mar Genomics*. 2015; doi: 10.1016/j.margen.2015.09.007.

25. Alama-Bermejo G, Holzer AS. Advances and discoveries in myxozoan genomics. *Trends Parasitol*. 2021; doi: 10.1016/j.pt.2021.01.010.

26. D'Ambra I, Lauritano C. A review of toxins from Cnidaria. *Mar Drugs*. 2020; doi: 10.3390/md18100507.

27. NCBI Resource Coordinators N. Database resources of the National Center for Biotechnology Information. *Nucleic Acids Res*. 2015; doi: 10.1093/nar/gku1130.

28. Bushnell B. BBMap v38.73. https://sourceforge.net/projects/bbmap/. Accessed 25 May 2021.

29. Dainat J, Hereñú D, Pucholt P. NBISweden/AGAT: AGAT-v0.6.0. *Zenodo*. 2021; doi: 10.5281/zenodo.4637977.

30. Simão FA, Waterhouse RM, Ioannidis P, Kriventseva EV, Zdobnov EM. BUSCO: assessing genome assembly and annotation completeness with single-copy orthologs. *Bioinformatics*. 2015; doi: 10.1093/bioinformatics/btv351.

31. Afgan E, Baker D, Batut B, van de Beek M, Bouvier D, Čech M, et al.. The Galaxy platform for accessible, reproducible and collaborative biomedical analyses: 2018 update. *Nucleic Acids Res*. 2018; doi: 10.1093/nar/gky379.

32. Galaxy. https://usegalaxy.org/. Accessed 10 Aug 2021.

33. Lewin HA, Robinson GE, Kress WJ, Baker WJ, Coddington J, Crandall KA, et al.. Earth BioGenome Project: sequencing life for the future of life. *Proc Natl Acad Sci*. 2018; doi: 10/gdh5vz.

34. Huerta-Cepas J, Serra F, Bork P. ETE 3: Reconstruction, analysis and visualization of phylogenomic data. *Mol Biol Evol*. 2016; doi: 10/gfzpph.

35. Caswell TA, Droettboom M, Lee A, Hunter J, Firing E, Andrade ES de, et al.. Matplotlib release v3.3.1. *Zenodo*. 2020; doi: 10.5281/zenodo.3984190.

36. Waskom ML. Seaborn: statistical data visualization. *J Open Source Softw*. 2021; doi: 10.21105/joss.03021.

37. Inkscape Project IW. Inkscape. https://inkscape.org/. Accessed 21 Jan 2022.

38. Bayha KM, Dawson MN, Collins AG, Barbeitos MS, Haddock SHDD. Evolutionary relationships among scyphozoan jellyfish families based on complete taxon sampling and phylogenetic analyses of 18S and 28S ribosomal DNA. *Integr Comp Biol*. 2010; doi: 10.1093/icb/icq074.

39. Maronna MM, Miranda TP, Peña Cantero ÁL, Barbeitos MS, Marques AC. Towards a phylogenetic classification of Leptothecata (Cnidaria, Hydrozoa). *Sci Rep*. 2016; doi: 10/ggbrh4.

40. Mendoza-Becerril MA, Jaimes-Becerra AJ, Collins AG, Marques AC. Phylogeny and morphological evolution of the so-called bougainvilliids (Hydrozoa, Hydroidolina). *Zool Scr*. 2018; doi: 10/gd4ftz.

41. Elliott TA, Gregory TR. What's in a genome? The C-value enigma and the evolution of eukaryotic genome content. *Phil Trans R Soc B*. 2015; doi: 10/gfkjbq.

42. Wilbrandt J, Misof B, Niehuis O. COGNATE: comparative gene annotation characterizer. *BMC Genomics*. 2017; doi: 10/ggbrjp.

43. Tardent P. Coelenterata, Cnidaria. 1st ed. Jena/Stuttgart: Gustav Fischer; 1978.

44. Kubota S. Systematic study on a bivalve-inhabiting hydroid *Eucheilota intermedia* Kubota from central Japan. *J Fac Sci Hokkaido Univ Ser VI Zool*. 1985;24:pl. I.

45. Kubota S. Taxonomic study on *Hydrocoryne miurensis* (Hydrozoa). *Publ SETO Mar Biol*

Lab. 1988;33:1–18.

46. Kubota S. Second finding of *Stylactaria piscicola* (Komai, 1932) comb. nov. (Hydrozoa: Hydractiniidae) from off Atsumi Peninsula, Japan. *Publ Seto Mar Biol Lab*. 1991;35:11–5.

47. Kubota S. Chromosome number of a bivalve-inhabiting hydroid, *Eugymnanthea japonica* (Leptomedusae: Eirenidae) from Japan. *Publ SETO Mar Biol Lab*. 1992;35:383–6.

48. Ping Guo. The karyotype of *Rhopilema esculenta*. *J Fish China*. 1994;18:253–5.

49. Anokhin B, Kuznetsova V. Chromosome morphology and banding patterns in *Hydra oligactis* Pallas and *H. circumcincta* Schultze (Hydroidea, Hydrida). *FOLIA Biol-KRAKOW*. 1999;47:91–6.

50. Anokhin B, Nokkala S. Characterization of C-heterochromatin in four species of Hydrozoa (Cnidaria) by sequence specific fluorochromes Chromomycin A $_3$ and DAPI. *Caryologia*. 2004; doi: 10.1080/00087114.2004.10589387.

51. Anokhin BA, Kuznetsova VG. FISH-based karyotyping of *Pelmatohydra oligactis* (Pallas, 1766), *Hydra oxycnida* Schulze, 1914, and *H. magnipapillata* Itô, 1947 (Cnidaria, Hydrozoa). *Comp Cytogenet*. 2018; doi: 10/gfst43.

52. Pflug JM, Holmes VR, Burrus C, Johnston JS, Maddison DR. Measuring genome sizes using read-depth, k-mers, and flow cytometry: methodological comparisons in beetles (Coleoptera). *G3 (Bethesda)*. 2020; doi: 10.1534/g3.120.401028.

53. Lawley JW, Gamero-Mora E, Maronna MM, Chiaverano LM, Stampar SN, Hopcroft RR, et al.. The importance of molecular characters when morphological variability hinders diagnosability: systematics of the moon jellyfish genus *Aurelia* (Cnidaria: Scyphozoa). *PeerJ*. 2021; doi: 10.7717/peerj.11954.

54. GigaDB. http://gigadb.org. Accessed 1 Apr 2021.

55. Hydra 2.0 Web Portal. https://research.nhgri.nih.gov/hydra/. Accessed 1 Apr 2021.

56. OIST Marine Genomics Unit Genome Browser. https://marinegenomics.oist.jp/gallery. Accessed 1 Apr 2021.

57. MARIMBA. http://marimba.obs-vlfr.fr/. Accessed 1 Apr 2021.

58. IRIDIAN GENOMES. https://www.iridiangenomes.com/. Accessed 1 Apr 2021.

59. Li Y, Gao L, Pan Y, Tian M, Li Y, He C, et al.. Chromosome-level reference genome of the jellyfish *Rhopilema esculentum*. *GigaScience*. 2020; doi: 10.1093/gigascience/giaa036.

60. Galliot B, Schummer M. 'Guessmer' screening strategy applied to species with AT-rich coding sequences. *Trends Genet*. 1993; doi: 10.1016/0168-9525(93)90051-I.

61. Hoff K, Stanke M. Current methods for automated annotation of protein-coding genes. *Curr Opin Insect Sci*. 2015; doi: 10.1016/j.cois.2015.02.008.

62. Peona V, Blom MPK, Xu L, Burri R, Sullivan S, Bunikis I, et al.. Identifying the causes and consequences of assembly gaps using a multiplatform genome assembly of a bird-of-paradise. *Mol Ecol Resour*. 2020; doi: 10.1111/1755-0998.13252.

63. Nong W, Cao J, Li Y, Qu Z, Sun J, Swale T, et al.. Jellyfish genomes reveal distinct homeobox gene clusters and conservation of small RNA processing. *Nat Commun*. 2020; doi: 10.1038/s41467-020-16801-9.

64. Xia W-X, Li H-R, Ge J-H, Liu Y-W, Li H-H, Su Y-H, et al.. High-continuity genome assembly of the jellyfish *Chrysaora quinquecirrha*. *Zool Res*. 2021; doi: 10.24272/j.issn.2095-8137.2020.258.

65. Putnam NH, Srivastava M, Hellsten U, Dirks B, Chapman J, Salamov A, et al.. Sea anemone genome reveals ancestral eumetazoan gene repertoire and genomic organization. *Science*. 2007; doi: 10.1126/science.1139158.

66. Taguchi T, Tagami E, Mezaki T, Sekida S, Chou Y, Soong K, et al.. Recent progress of molecular cytogenetic study on scleractinian (stony) corals. *Kuroshio Sci*. 2017;9.

67. Technau U, Robb S, Genikhovich G, Montenegro J, Fropf W, Weinguny L, et al.. Sea anemone genomes reveal ancestral metazoan chromosomal macrosynteny. *Res Sq*. 2021; doi: 10.21203/rs.3.rs-796229/v1.

68. Blommaert J. Genome size evolution: towards new model systems for old questions. *Proc R Soc B Biol Sci*. 2020; doi: 10.1098/rspb.2020.1441.

69. Wong WY, Simakov O, Bridge DM, Cartwright P, Bellantuono AJ, Kuhn A, et al.. Expansion of a single transposable element family is associated with genome-size increase and radiation in the genus *Hydra*. *Proc Natl Acad Sci*. 2019; doi: 10/ggdfjb.

70. Schrader L, Schmitz J. The impact of transposable elements in adaptive evolution. *Mol Ecol*. 2019; doi: 10.1111/mec.14794.

71. Cosby RL, Judd J, Zhang R, Zhong A, Garry N, Pritham EJ, et al.. Recurrent evolution of vertebrate transcription factors by transposase capture. *Science*. 2021; doi: 10.1126/science.abc6405.

72. Hamada M, Satoh N, Khalturin K. A reference genome from the symbiotic hydrozoan, *Hydra viridissima*. *G3 (Bethesda).* 2020; doi: 10.1534/g3.120.401411.

73. Kim H-M, Weber JA, Lee N, Park SG, Cho YS, Bhak Y, et al.. The genome of the giant Nomura's jellyfish sheds light on the early evolution of active predation. *BMC Biol*. 2019; doi: 10/gfxm7p.

74. Xia W, Li H, Cheng W, Li H, Mi Y, Gou X, et al.. High-quality genome assembly of *Chrysaora quinquecirrha* provides insights into the adaptive evolution of jellyfish. *Front Genet*. 2020; doi: 10.3389/fgene.2020.00535.

75. Graur D, Zheng Y, Azevedo RBR. An evolutionary classification of genomic function. *Genome Biol Evol*. 2015; doi: 10.1093/gbe/evv021.

76. Chapman J a, Kirkness EF, Simakov O, Hampson SE, Mitros T, Weinmaier T, et al.. The dynamic genome of *Hydra*. *Nature*. 2010; doi: 10.1038/nature08830.

77. Galliot B. *Hydra*, a fruitful model system for 270 years. *Int J Dev Biol*. 2012; doi: 10.1387/ijdb.120086bg.

78. Tomczyk S, Fischer K, Austad S, Galliot B. *Hydra*, a powerful model for aging studies. *Invertebr Reprod Dev*. 2015; doi: 10.1080/07924259.2014.927805.

79. Weisman CM, Murray AW, Eddy SR. Many, but not all, lineage-specific genes can be explained by homology detection failure. *PLOS Biol*. 2020; doi: 10.1371/journal.pbio.3000862.

80. Weisman CM, Murray AW, Eddy SR. Mixing genome annotation methods in a comparative analysis inflates the apparent number of lineage-specific genes. *bioRxiv*; 2022;

81. Chen Y, González-Pech RA, Stephens TG, Bhattacharya D, Chan CX. Evidence that inconsistent gene prediction can mislead analysis of dinoflagellate genomes. *J Phycol*. 2020;

doi: 10.1111/jpy.12947.

82. Martín-Durán JM, Ryan JF, Vellutini BC, Pang K, Hejnol A. Increased taxon sampling reveals thousands of hidden orthologs in flatworms. *Genome Res*. 2017; doi: 10.1101/gr.216226.116.

83. Natsidis P, Kapli P, Schiffer PH, Telford MJ. Systematic errors in orthology inference and their effects on evolutionary analyses. *iScience*. 2021; doi: 10.1016/j.isci.2021.102110.

84. Quattrini AM, Rodríguez E, Faircloth BC, Cowman PF, Brugler MR, Farfan GA, et al.. Palaeoclimate ocean conditions shaped the evolution of corals and their skeletons through deep time. *Nat Ecol Evol*. 2020; doi: 10.1038/s41559-020-01291-1.

85. Schriml LM, Chuvochina M, Davies N, Eloe-Fadrosh EA, Finn RD, Hugenholtz P, et al.. COVID-19 pandemic reveals the peril of ignoring metadata standards. *Sci Data*. 2020; doi: 10.1038/s41597-020-0524-5.

86. Martinez PA, Jacobina UP, Fernandes RV, Brito C, Penone C, Amado TF, et al.. A comparative study on karyotypic diversification rate in mammals. *Heredity*. 2017; doi: 10.1038/hdy.2016.110.

87. Toczydlowski RH, Liggins L, Gaither MR, Anderson TJ, Barton RL, Berg JT, et al.. Poor data stewardship will hinder global genetic diversity surveillance. *Proc Natl Acad Sci*. 2021; doi: 10.1073/pnas.2107934118.

88. Bayer PE, Edwards D, Batley J. Bias in resistance gene prediction due to repeat masking. *Nat Plants*. 2018; doi: 10.1038/s41477-018-0264-0.

89. Fiddes IT, Armstrong J, Diekhans M, Nachtweide S, Kronenberg ZN, Underwood JG, et al.. Comparative Annotation Toolkit (CAT)—simultaneous clade and personal genome annotation. *Genome Res*. 2018; doi: 10.1101/gr.233460.117.

90. König S, Romoth LW, Gerischer L, Stanke M. Simultaneous gene finding in multiple genomes. *Bioinforma Oxf Engl*. 2016; doi: 10.1093/bioinformatics/btw494.

91. Dunne MP, Kelly S. OMGene: mutual improvement of gene models through optimisation of evolutionary conservation. *BMC Genomics*. 2018; doi: 10.1186/s12864-018-4704-z.

92. Dunne MP, Kelly S. OrthoFiller: utilising data from multiple species to improve the

completeness of genome annotations. *BMC Genomics*. 2017; doi: 10.1186/s12864-017-3771-x.

93. Hua Z, Early MJ. Closing target trimming and CTTdocker programs for discovering hidden superfamily loci in genomes. *PLOS ONE*. 2019; doi: 10.1371/journal.pone.0209468.

94. Kim S, Cheong K, Park J, Kim M-S, Kim J, Seo M-K, et al.. TGFam-Finder: a novel solution for target-gene family annotation in plants. *New Phytol*. 2020; doi: 10.1111/nph.16645.

95. Ottenburghs J, Geng K, Suh A, Kutter C. Genome size reduction and transposon activity impact tRNA gene diversity while ensuring translational stability in birds. *Genome Biol Evol*. 2021; doi: 10.1093/gbe/evab016.

96. Arita M, Karsch-Mizrachi I, Cochrane G, on behalf of the International Nucleotide Sequence Database Collaboration. The international nucleotide sequence database collaboration. *Nucleic Acids Res*. 2021; doi: 10.1093/nar/gkaa967.

97. Kodama Y, Shumway M, Leinonen R. The Sequence Read Archive: explosive growth of sequencing data. *Nucleic Acids Res*. 2012; doi: 10/fw3c92.

98. Hemmrich G, Bosch TC. Compagen, a comparative genomics platform for early branching metazoan animals, reveals early origins of genes regulating stem- cell differentiation. *Bioessays*. Wiley Online Library; 2008;30:1010–8.

99. Ryan JF, Finnerty JR. CnidBase : The Cnidarian Evolutionary Genomics Database. *Nucleic Acids Res*. 2003; doi: 10.1093/nar/gkg116.

100. Wilkinson MD, Dumontier M, Aalbersberg IjJ, Appleton G, Axton M, Baak A, et al.. The FAIR Guiding Principles for scientific data management and stewardship. *Sci Data*. 2016; doi: 10.1038/sdata.2016.18.

101. GIGA Community of Scientists. The Global Invertebrate Genomics Alliance (GIGA): developing community resources to study diverse invertebrate genomes. *J Hered*. 2014; doi: 10.1093/jhered/est084.

102. Ewels P, Magnusson M, Lundin S, Käller M. MultiQC: summarize analysis results for multiple tools and samples in a single report. *Bioinformatics*. 2016; doi:

10.1093/bioinformatics/btw354.

103. Challis R, Richards E, Rajan J, Cochrane G, Blaxter M. BlobToolKit – interactive quality assessment of genome assemblies. *G3 (Bethesda)*. 2020; doi: 10.1534/g3.119.400908.

104. Liu F, Li Y, Yu H, Zhang L, Hu J, Bao Z, et al.. MolluscDB: an integrated functional and evolutionary genomics database for the hyper-diverse animal phylum Mollusca. *Nucleic Acids Res*. 2021; doi: 10.1093/nar/gkaa918.

105. Storer J, Hubley R, Rosen J, Wheeler TJ, Smit AF. The Dfam community resource of transposable element families, sequence models, and genome annotations. *Mob DNA*. 2021; doi: 10.1186/s13100-020-00230-y.

106. Fautin DG, Westfall JA, Cartwright P, Daly M, Wyttenbach CR. Coelenterate Biology 2003: trends in research on Cnidaria and Ctenophora. *Hydrobiologia*. 2005;530:11–3.

107. He S, Grasis JA, Nicotra ML, Juliano CE, Schnitzler CE. Cnidofest 2018: the future is bright for cnidarian research. *Evodevo*. 2019; doi: 10.1186/s13227-019-0134-5.

108. Funayama N, Frank U. Meeting report on "At the roots of bilaterian complexity: insights from early emerging metazoans," Tutzing (Germany) September 16–19, 2019. *BioEssays*. 2020; doi: 10.1002/bies.201900236.

109. Paps J, Holland PWH. Reconstruction of the ancestral metazoan genome reveals an increase in genomic novelty. *Nat Commun*. 2018; doi: 10.1038/s41467-018-04136-5.

110. Guijarro-Clarke C, Holland PWH, Paps J. Widespread patterns of gene loss in the evolution of the animal kingdom. *Nat Ecol Evol*. 2020; doi: 10.1038/s41559-020-1129-2.

111. Dawson MN, Hamner WM. A character-based analysis of the evolution of jellyfish blooms: adaptation and exaptation. *Hydrobiologia*. 2009; doi: 10.1007/s10750-008-9591-x.

112. Deakin JE, Potter S, O'Neill R, Ruiz-Herrera A, Cioffi MB, Eldridge MD, et al.. Chromosomics: Bridging the gap between genomes and chromosomes. *Genes*. 2019; doi: 10.3390/genes10080627.

113. Dimitrova M, Meyer R, Buttigieg PL, Georgiev T, Zhelezov G, Demirov S, et al.. A streamlined workflow for conversion, peer review, and publication of genomics metadata as omics data papers. *GigaScience*. 2021; doi: 10.1093/gigascience/giab034.

114. Santander MD, Maronna MM, Ryan JF, Andrade S. The state of Medusozoa genomics: supplementary material. doi: 10.6084/m9.figshare.17155676

115. Hydractinia Genome Project Portal. https://research.nhgri.nih.gov/hydractinia/. Accessed 1 Apr 2021.

116. Vogg MC, Beccari L, Iglesias Ollé L, Rampon C, Vriz S, Perruchoud C, et al.. An evolutionarily-conserved Wnt3/β-catenin/Sp5 feedback loop restricts head organizer activity in *Hydra*. *Nat Commun*. 2019; doi: 10.1038/s41467-018-08242-2.

117. Migotto AE, Vellutini BC. Cifonauta: Banco de Imagens de Biologia Marinha. http://cifonauta.cebimar.usp.br/. Accessed 2 Feb 2022.

Table 1 - Genomic projects related to Medusozoa HTS. Sequencing projects with no current related publication are remarked with capital letters. Column "Main research topics" describes keywords according to references, restricted to a maximum of 4; "gene evolution" refers to the study of gene gains/losses and also of specific gene families. Species with reported assemblies were re-analyzed in this review (bold; Supplementary file S5 Table S3). UMCG=University Medical Center Groningen; IISER PRune=Indian Institute of Science Education and Research, Pune; NHGRI=The National Human Genome Research Institute; TF=transcription factors; *"preliminary" assembly available at the institutional site; **species with taxonomic updates. For further details see Supplementary file S1.

Project	Release year (NCBI-SRA)	Class (n° genomes)	Species	Main research topics
Chapman et al. [76]	2008	Hydrozoa (1)	***Hydra vulgaris***	Gene evolution; micro-synteny
IISER Pune	2014-2015	Hydrozoa (1)	***Hydra vulgaris***	not_informed
NHGRI [55]	no SRA	Hydrozoa (1)	***Hydra vulgaris***	not_informed
NHGRI [115]	2016	Hydrozoa (1)	*Hydractinia echinata**	not_informed
Gold et al. [19]	2018	Scyphozoa (1)	***Aurelia coerulea***	Life cycle; gene evolution; intraspecies variability; HOX
IRIDIAN	2018	Hydrozoa (1)	***Craspedacusta sowerbii***	not_informed

GENOMES [58]				
Kim et al. [73]	2018	Scyphozoa (1)	***Nemopilema nomurai***	Life cycle; jellyfish body patterning; gene evolution; toxins
IRIDIAN GENOMES [58]	2019	Hydrozoa (1)	*Scolionema suvaense*	not_informed
Khalturin et al. [20]	2019	Scyphozoa (2)	***Aurelia aurita**, Aurelia coerulea****	Life cycle; jellyfish body plan; gene evolution; synteny
		Cubozoa (1)	***Morbakka virulenta***	
Leclère et al. [21]	2019	Hydrozoa (1)	***Clytia hemisphaerica***	Life cycle; gene evolution; micro-synteny; TF
Odhera et al. [22]	2019	Scyphozoa (1)	***Cassiopea xamachana***	Gene evolution; micro-synteny; Homeobox; toxins
		Cubozoa (1)	***Alatina alata***	
		Staurozoa (1)	***Calvadosia cruxmelitensis***	
Vogg et al. [116]	2019	Hydrozoa (1)	***Hydra oligactis*; *Hydra viridissima***	Gene evolution; RTKs; developmental genes
Hamada et al. [72]	2020	Hydrozoa (1)	***Hydra viridissima***	Symbiosis; immune response; repetitive DNA; Homeobox
IRIDIAN GENOMES [58]	2020	Cubozoa (3)	***Alatinidae* sp.**	not_informed
			Carybdea marsupialis	
			Tamoya ohboya	
		Hydrozoa (2)	*Cladonema radiatum*	
			Eutima sp. BMK-2020	
		Scyphozoa (4)	***Aurelia coerulea***	
			Chrysaora achlyos	
			Chrysaora chesapeakei	
			Chrysaora fuscescens	
		Staurozoa (1)	*Calvadosia cruxmelitensis*	
Li et al. [59]	2020	Scyphozoa (1)	***Rhopilema esculentum***	Gene evolution; toxins
Nong et al. [63]	2020	Scyphozoa (2)	***Sanderia malayensis, Rhopilema esculentum***	Gene evolution; small RNAs; micro-synteny; Homeobox
Xia et al. [74]	2020	Scyphozoa (1)	***Chrysaora quinquecirrha***	Gene and gene feature evolution; repetitive DNA
Xia et al. [64]	2020	Scyphozoa (1)	***Chrysaora quinquecirrha***	Assembly improvement report
UMCG	2021	Scyphozoa (1)	***Cassiopea andromeda***	not_informed

Figure 1 - Medusozoa diversity. Examples of different genus covered by this review belong to Hydrozoa (A-B), Staurozoa (C), Cubozoa (D-E) and Scyphozoa (F-G). A) *Craspedacusta sowerbii,* B) *Cladonema radiatum,* C) *Haliclystus sanjuanensis*, D) Carybdea sivickisi, E) *Tamoya haplonema*, F) *Cassiopea xamachana*, G) *Aurelia aurita*. Credits to Alvaro E. Migotto (A, B, E), Marta Chiodin (C), Joseph Ryan (F, G) and Cheryl Ames Lewis (D). Photographs A, B, D, E were obtained from Cifonauta [117]. Photographs are not to scale.

Figure 2 - Phylogenetic distribution of genomic information in Medusozoa. A) Number of described species and number of species with genomic data; B) Chromosome number (2n) range; C) Genome size (Mbp) range taking into account Flow Cytometry and Feulgen Densitometry estimations; D) Total number of available assemblies and number of species with assembled genomes. In B) and C) single values were also included when only one species was characterized. Tree topology is explained in the methods section. Information used for this graph is available at Supplementary file S5 Table S2.

Figure 3 - Assembly and genome features. In A) is reported (from left to right): mean assembly length per class, GC content (%) per class, number of contigs and scaffolds per assembly coloured by class, contig and scaffold N50 (in Kbp) per assembly coloured by class, and count of assemblies of each class corresponding to the different BGP-metric values, where X and Y correspond to contig and scaffold N50 respectively, and Z to chromosome assignment (see methods section). In B) is reported (from left to right): mean repeat length (Mbp) in assembly per class, mean total number of genes per class, mean exon number (count per gene) per class, and mean gene, intron and exon length (Kbp) per assembly coloured by class. The yellow arrowhead indicates *S. malayensis* gene features (See Box). All other references are specified in the figure. Mbp=millions of base pairs. Information used for this graph is available at Supplementary file S5 Tables S4-6.

Figure 4 - BUSCO Metazoa gene distribution in Medusozoa assemblies. Each column corresponds to a gene and each row an assembly. Columns were ordered based on presence

from left to right and the least present genes (n=96) are shown in detail. Genes absent in all or almost all assemblies (more than 80% of absence) are indicated in red; genes also reported absent [20] are indicated in bold; genes absent in specific lineages are indicated with yellow rectangles. Higher quality assemblies are indicated in orange (BGP-metric > 1.0.0). The assembly with the highest quality score for BGP-metric is indicated by an orange circle and corresponds to *Rhopilema esculentum* [59]. Information used for this graph and full BUSCO gene names are available at Supplementary file S5 Table S7.

Supplementary Material

Supplementary file S1. Dataset 1. Genome report sheet.

Supplementary file S2. Dataset 2. Command line to retrieve data from NCBI and to generate new results.

Supplementary file S3. Dataset 3. Scripts used for graph construction.

Supplementary file S4. Table S1. Species information considering chromosome number, genome size and genomic datasets.

Supplementary file S5. Tables S2-8 - All information used for constructing graphs presented in this work. Includes summary information of Figure 2 (table S2), genome resources used in this study (table S3), assembly statistics for Figure 3A (table S4), genome features of Figure 3B (table S5, S6) and BUSCO results for Figure 4 and Supplementary figure S1 (tables S7, S8).

Supplementary file S6. Figure S1. BUSCO Eukaryota gene distribution in Medusozoa assemblies. Each column corresponds to a gene and each row an assembly. Information used for this graph is available at Supplementary file S4 Table S8.

Supplementary file S7 - Dataset 4. Original metadata from NCBI.

Supplementary file S8 - Dataset 5. Original results from AGAT and Galaxy server (BUSCO).

Supplementary file S9 - Dataset 6. Figures in vectorial format.

Figure 1

Click here to access/download;Figure;1 Figure 1.TIFF

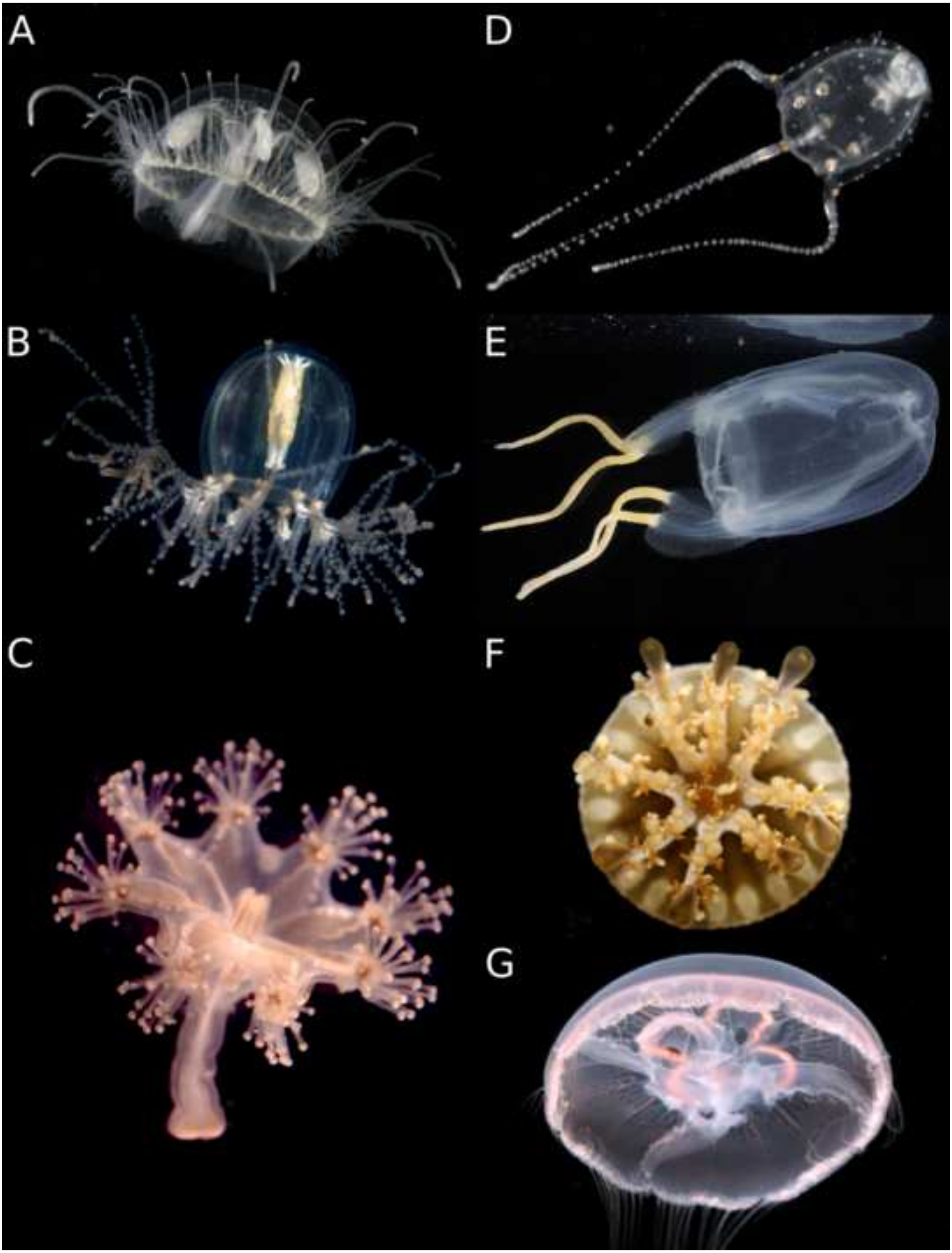

Figure 2

Click here to access/download;Figure;2 Figure 2.TIFF

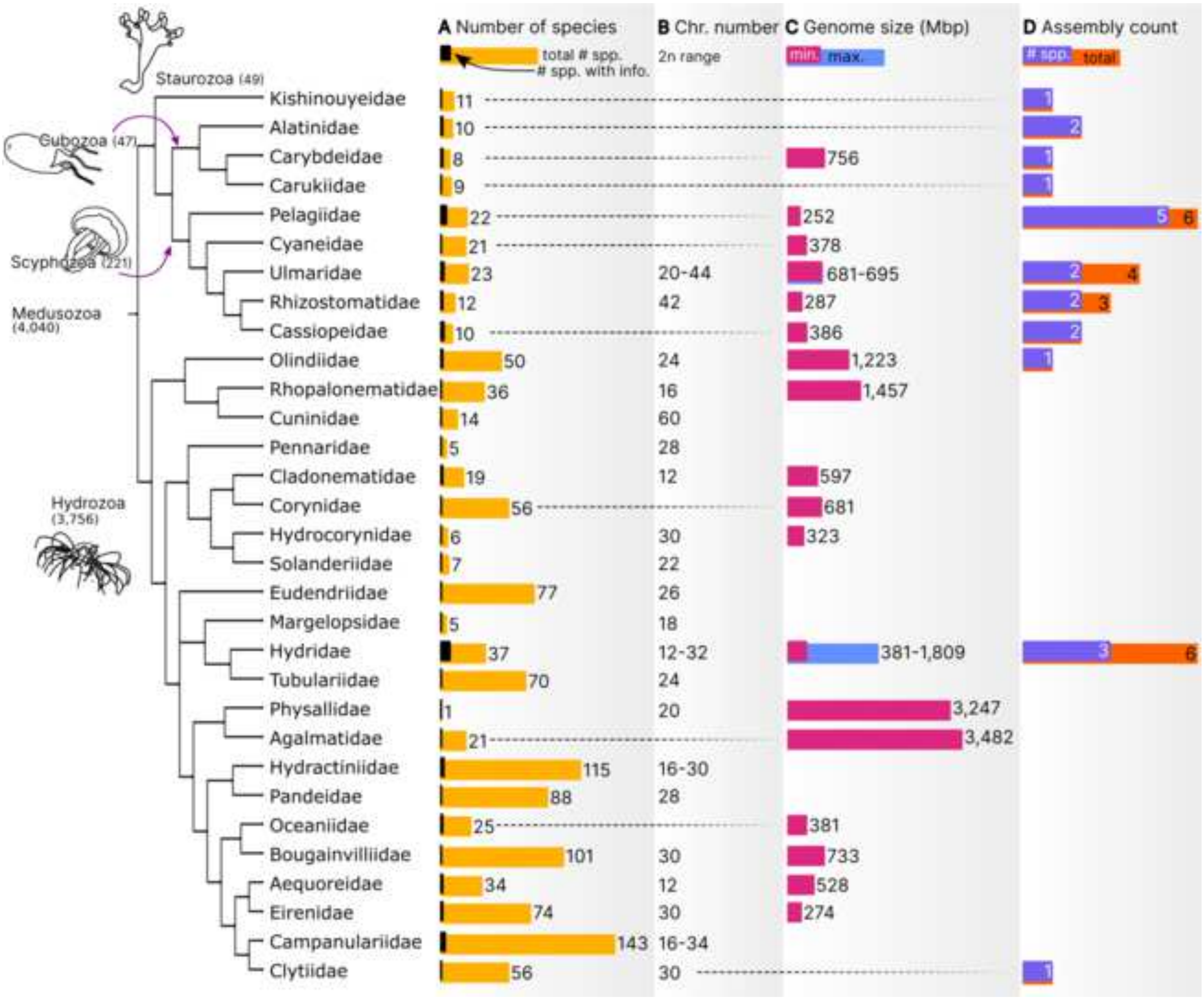

Figure 3

Click here to access/download;Figure;3 Figure 3.TIFF

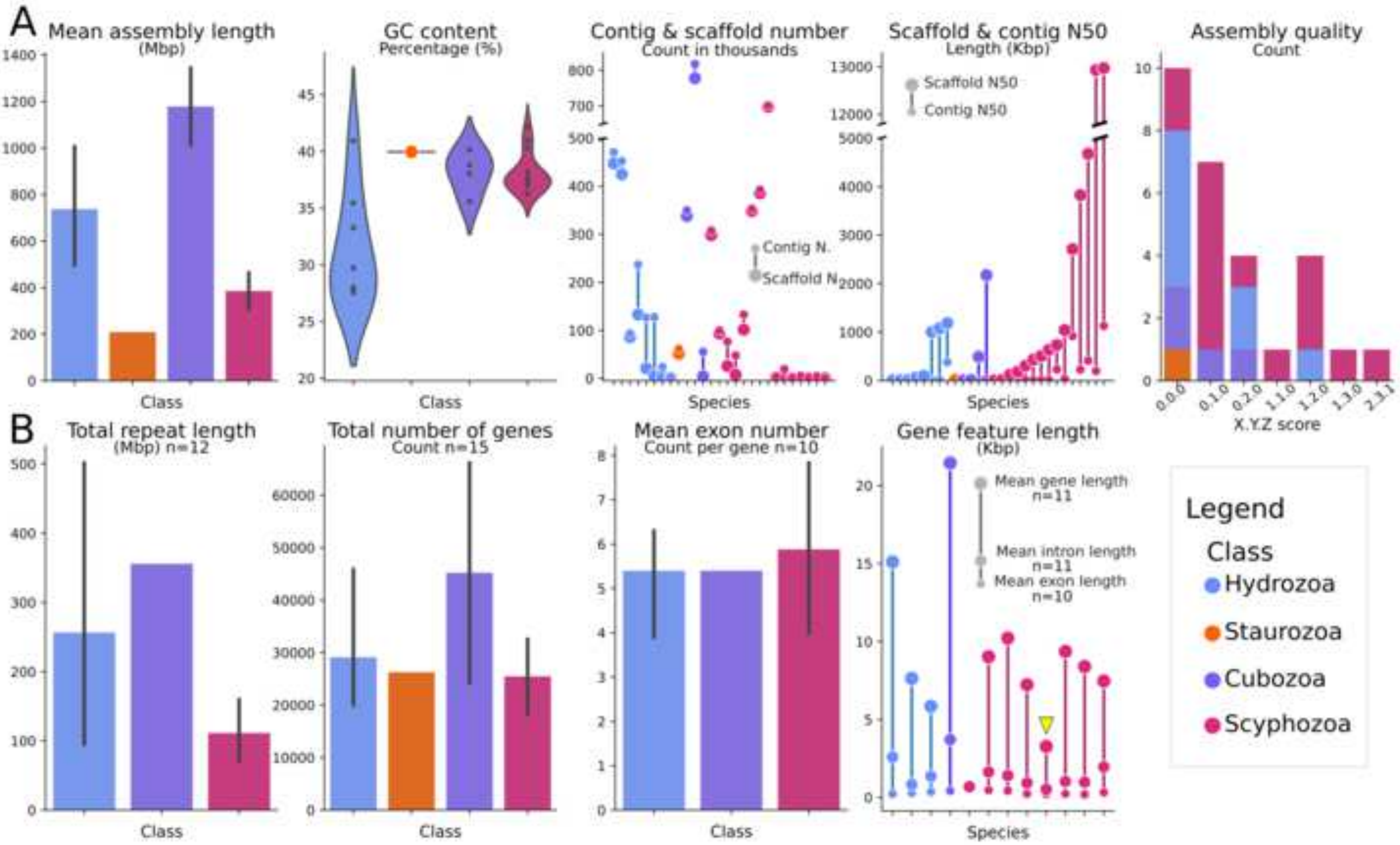

Figure 4

Click here to access/download;Figure;4 Figure 4.TIFF

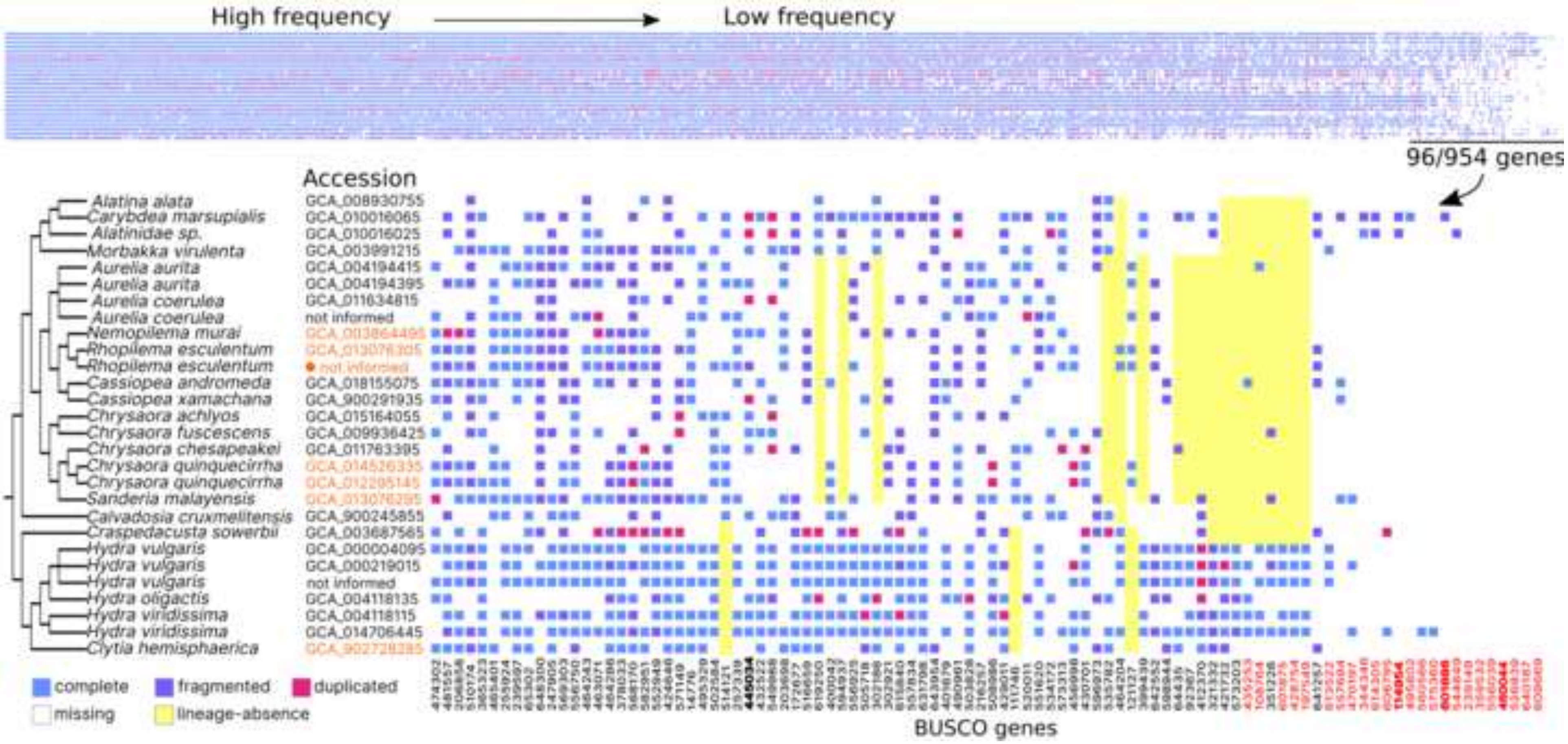

Supplementary File 1

Click here to access/download
Supplementary Material
Supplementary_file_S1_Dataset_1_genome_report_sheet.xlsx

Supplementary File 2

Click here to access/download
Supplementary Material
Supplementary_file_S2_command_line.sh

Click here to access/download
Supplementary Material
Supplementary_file_S3_Codes_for_graphs.py

Supplementary File 4

Click here to access/download
Supplementary Material
Supplementary_file_S4_Table_S1_Chr_GenomeSize.xlsx

Supplementary File 5

Click here to access/download
Supplementary Material
Supplementary_file_S5_TablesS2-8_Figures_Information.xlsx

Supplementary File 6

Click here to access/download
Supplementary Material
Supplementary_file_S6_Figure_S1_EukaryotaBUSCO.png

Manuscript with track changes

Click here to access/download
Supplementary Material
Main_Text_Revision1_trackchanges.docx

Click here to access/download;Personal Cover;Response to reviewers.docx

Editor

"Your manuscript "The state of Medusozoa genomics: past evidence and future challenges" (Review Article; GIGA-D-21-00404) has been assessed by three reviewers. Based on these reports, I am pleased to inform you that it is potentially acceptable for publication in GigaScience, once you have carried out some essential revisions suggested by our reviewers. Their reports are below.
I'd like to highlight three points:"

We are very appreciative of the excellent suggestions from the reviewers and editor. We have done our best to address each point and we feel that the manuscript has been greatly improved as a result of the review process. Thank you for the time dedicated to our manuscript. We provide a point-by-point answer to each suggestion. We also provide a new main text and a copy of the original text with all the changes kept as tracks. Line numbers in this letter are referenced to the new main text file in de submission PDF. Original comments made by the editor and the reviewers are indicated in bold or between quotation marks. We also provide a formated copy of the response to the reviewers as a separate file at the end of the submission PDF.

"1. Two of the reviewers mention that the "recommendations" would benefit if it would make clearer if there are any Medusozoa-specific recommendations (in addition to advice that is generally applicable to all animal genome projects)"

We have added the following to address this point generally on line 422:

> The following are suggestions to enhance genome projects and outcomes, and to promote open and collaborative research. These suggestions can be broadly applied to any genome project and are in line with those proposed by many initiatives and consortia (e.g. [33,100,101]). Nevertheless, it is worth reinforcing and discussing them in the context of this review since genome projects are more and more often being initiated in research laboratories that have historically been more focused on other aspects of medusozoan biology and may not be as familiar with these general practices:

We have added the following to point #3 that refers to where to deposit data on lines 446:

> A Medusozoa-centric database with long-term maintenance is still lacking for the community (e.g. Mollusca clade [104]); but many open repositories can serve this purpose with low or no costs considering the size of the aforementioned outputs. There are open topic-centric repositories (e.g Dfam [105] for repetitive DNA), general repositories (e.g. FigShare, Zenodo; or even NCBI for annotation tracks) as well as personal or institutional ones. Many of the reviewed genomic projects already made use of these repositories but failed to deposit some of the outputs. A solution for this inconvenience is to update submissions or create novel ones (e.g. submit annotations to NCBI or ENA) to deposit the missing outputs.

"2. Reviewer 1 recommends to make your code public, and I strongly support this, as it is also in line with our journal guidelines. You can also host code and supporting data in our repository GigaDB - our data curators will be happy to help. Please attach an open (OSI-compliant) licence to any scripts/code. (https://opensource.org/licenses)"

All the command lines used in this work were originally specified in the Supplementary File S7 of the original submission (Supplementary File S2 in the current version) but it was not properly indicated in the material and methods section. We corrected this issue by adding the following sentence on lines 122:

> The command line used for retrieving genetic information and metadata, for statistics calculation and the code used for graph generation are available at Supplementary file S2 and S3.

We have also added the scripts used for constructing graphs in Supplementary file S3 (as suggested by reviewer 1). All the software used in this work is open and was properly referenced.

We deposited all supplementary files in Figshare and GigaDB and included a statement of open license to scripts on lines 518:

> **Data availability**
> All collected information, outputs and scripts supporting new results are available in the supplementary files S1-S9 in Figshare [114] and in GigaDB [115].

"3. Although not mentioned by the reviewers, I feel your manuscript would be more interesting for readers from outside the medusozoa community if you explained in a bit more detail the actual biological questions that have been addressed with these genomes; such as toxins, metazoan evolution / body plan evolution, Hox genes, immunity, etc.. These topics are mentioned in the introduction, but I feel they could be picked up again in a bit more detail in the discussion, to illustrate the biological insights gained from the genome projects."

We have added two paragraphs that highlight the insight genome projects bring understanding medusozoa biology.

Starting on line 301:

> The complex nature of Medusozoa venom has been investigated by a number of transcriptomic, proteomic and genomic studies (reviewed in [26]). Several putative toxin genes and domains have been identified, covering a significant part of the wide range of known toxins [20,22,59,73]. In Scyphozoa, toxin-like genes were often recovered as multicopy sets [20,59]. Moreover, in *R. esculentum* toxin-like genes were also tandemly arranged and several of them were located nearby in chromosome 7, suggesting that the observed organization might influence toxin co-expression[59]. Minicollagens, which are major components of nematocysts, also had a clustered organization and a pattern of co-expression in Aurelia [20]. These examples add to

> various clustered genes described in Cubozoa, Hydrozoa and Anthozoa, and would indicate that gene clustering and operon-like expression of toxin genes is widespread in Cnidaria ([20] and references therein).

and starting on line 329:

> The complex life cycle of Medusozoa has resulted from the combination of both ancestral and novel features. *Aurelia*, *Morbakka virulenta* and *Clytia hemisphaerica* have significantly different patterns of gene expression across stages and during transitions [19–21]. Differentially expressed genes include many conserved ancestral families of transcription factors [19–21]; there is also a considerable amount of the putative lineage-restricted genes that show differential expression in the adult stages [20,21]. A few of these “novel” medusozoan genes have been described, such as novel myosin-tail proteins that are absent from Anthozoa and represent markers of the medusae striated muscles [20]. It was suggested that the evolution of the Medusozoa complex life cycle would therefore have involved the rewiring of regulatory pathways of ancestral genes and the contribution of new ones [19–21]. As such, the body plan and life cycle simplifications observed in *Clytia* and *Hydra*, respectively, would be the result of loss of transcription factors involved in their development [21]. Finally, the significance of many of the putative Medusozoa and species-specific genes remain to be elucidated.

"4. For a review article, please also feel free to add illustrations/photos of relevant medusozoa species, if you wish (but please check with any copyright holder, if applicable - images will be published under an open cc-by licence)."

We added a new figure (Figure 1) with photographs of example species of each Medusozoa class. Some photographs (Figure 1 A, B, D, E) were recovered from an online open database called Cifonauta, available under open cc-by license, and it was properly cited. The remaining photographs were provided by Marta Chiodin (Figure 1C), Joseph Ryan (co-author; Figure 1 F, G), with permission to publish under CC-BY license. As a result of the addition of a new Figure 1, all figures were renumbered accordingly.

"Reviewer 1"

"In this paper, Santander et al. review the field of medusozoan genomics, which has burgeoned in the last three or so years. Overall, I found this a clear, interesting read. The manuscript is well-written, the figures are valuable, and the authors nicely describe the history of the research as well as the state of the field. The findings are not monumental, but it is a worthwhile exercise to survey the rapidly-increasing dataset of genomes in a systematic way, and this review will be a useful start for further work in medusozoan comparative genomics. I rarely suggest a paper should be accepted during the first round of review, and I usually try to provide more constructive feedback than I do here, but I really don't have much too much to quibble with. A couple thoughts are provided below:

1. The set of suggestions for future work near the end of the document are fine, but they could apply broadly to any genome project. I encourage the authors to consider whether there are specific problems related to medusozoan evolution that are hampered by inconsistencies between studies, and discuss how their recommendations (or additional ones) could help resolve them."

This comment also addresses reviewer #3's first point as well. We have added the following, which acknowledges that some of our recommendations are general to all genome projects and provides justification for why it is important to include these in this review on line 422:

> The following are suggestions to enhance genome projects and outcomes, and to promote open and collaborative research. These suggestions can be broadly applied to any genome project and are in line with those proposed by many initiatives and consortia (e.g. [33,100,101]). Nevertheless, it is worth reinforcing and discussing them in the context of this review since genome projects are more and more often being initiated in research laboratories that have historically been more focused on other aspects of medusozoan biology and may not be as familiar with these general practices:

In the recommendation regarding depositing results in public databases we discussed its importance and how metadata can be improved when datasets were already made public on line 431:

> Frequently, data and metadata that are described in the original articles or deposited in repositories are not submitted to public databases. Tracking information from multiple sources is time consuming and prone to error. Databases and repositories enable the improvement of metadata after the initial releases, by the addition of new or corrected information (e.g. publication information) from the authors. We believe that this kind of data curation would improve the state of Medusozoa genomics not only by enabling downstream analysis after the publication, but also enabling the detection of methodological options (e.g. tissue selection; sequencing technology) that would improve the quality of the results.

In the section about depositing intermediate outputs, we have added information on the state of relevant taxon-specific databases on line 446:

> Medusozoa-centric database with long-term maintenance is still lacking for the community (e.g. Mollusca clade [104]); but many open repositories can serve this purpose with low or no costs considering the size of the aforementioned outputs.

We added a paragraph discussing potential problems and benefits related to proper method description on line 460.

> The latter suggestions (3-6) are mainly related to providing detailed methodologies of bioinformatic analyses. First, proper method and results descriptions can help to recover metadata and criteria usually not available in large sequence repositories. Second, comparative analyses depend upon standardization at different levels and significant sample sizes. The inclusion of species in downstream analyses is limited by

data availability and proper description of previous analyses, custom software and results.

We added a recommendation about engaging in community-wide discussions, and highlighted potential venues that would be appropriate for discussing medusozoan genomics standards starting on line 466:

> 7. Engage in community-driven conversations about standards, guidelines and species priorities. There are a number of taxon-specific meetings that would be appropriate venues to engage in these conversations including the International Conference on Coelenterate Biology (~decennial; [106]), the International Jellyfish Blooms Symposium (~triennial), Cnidofest (~biennial; [107]), Tutzing workshop (~biennial; [108]), and Cnidofest zoom seminar series. In addition, satellite meetings at larger annual meetings (e.g. the Society for Integrative and Comparative Biology (SICB) or the Global Invertebrate Genomics Alliance (GIGA [101])) could provide appropriate venues to facilitate discussions on how the community can best move forward as more and more genomic data come online.

We close the section with a paragraph that explains how adhering to standards will benefit the medusozoan community on line 475:

> The adoption of best practices in the Medusozoa genomics community will pave the way for major breakthroughs regarding understanding the genomic basis for several evolutionary innovations that arose within and in the stem lineage of Medusozoa. Similar advances were achieved with extensive taxon sampling at broader scales, where 25 novel core gene groups enriched in regulatory functions might be underlying the emergence of animals [109,110]. Medusozoa innovations have puzzled the community for decades [5,7,11,111] and include the origin of the medusa, the loss of polyp structures, the establishment of symbiosis, the blooming potential, and the evolution of an extremely potent venom. A deeper understanding of the genomic events driving these innovations will require accurate identifications of a number of key genomic features including (but not limited to) single copy orthologs, gene losses, lineage-specific genes, gene family expansions and non-coding regulatory sequences.

Related to this last point, we also suggest to read the added sentences after reviewer #3 comment on line 314:

> Recent evidence proved that the detection of lineage-specific genes, and other analyses relying on accurate annotation and orthology prediction, can be significantly biased by methodological artifacts [79–83]; several problems have been identified, such as low taxon sampling, heterogeneous gene predictions, and failure of detecting distant homology and fast-evolving orthologues. These considerations are highly relevant in Medusozoa, as comparisons are often made, by necessity, with distantly related species (e.g. Anthozoa has been estimated to have diverged from Medusozoa around 800 million years ago [84]).

"2. I would encourage the authors to practice what they preach in terms of transparency, and make the code they used in their methods public (e.g.

statswrapper.sh, AGAT, BUSCO, ETE Toolkit, Matplotlib, Seaborn). The code does not need to be executable, but a supplemental text and/or repository with as much of the starting data and commands executed as possible would make it easier for others to replicate this work and apply it to future comparative genomics projects."

All the command line used in this work was originally specified in the Supplementary S7 of the original submission but we did not not properly indicate this in the material and methods section. We corrected this issue by adding a sentence in the corresponding section as indicated below (note: this required re-numbering the supplementary files so Supplementary file S7 is now S2). We also included the scripts used for constructing graphs. All the packages and softwares used in the command line and in the custom scripts (statswrapper.sh, AGAT, BUSCO, ETE Toolkit, Matplotlib, Seaborn) are open. We have added the following on line 122:

> The command line used for retrieving genetic information and metadata, for statistics calculation and the code used for graph generation are available at Supplementary file S2 and S3.

"3. Line 236: "…ploidy level, heterochromatin contente." This should be changed to "…ploidy level, and heterochromatin content.""

This error was corrected.

"4. Line 253-254: "…evolution of genome size is a long-standing question that is included in the so-called C-value Enigma [40]." The authors provide a citation, but I think this sentence would be stronger with a brief explanation of what the C- value Enigma is. Medusozoans are a great example of this "enigma", so it's worth reinforcing."

We have added the following to clarify the C-value enigma on line 274:

> … "C-value Enigma" [41]. This name stems from the difficulty elucidating the evolutionary forces (e.g. drift and natural selection) that have given rise and serve to maintain variations in genome size, the mechanisms of genome size change, and the consequences of these variations at an organismal level [41]. Several conflicting hypotheses have been postulated to explain this puzzle with most having experimental support in some but not all lineages (reviewed in [68]).

Reviewer 2

"This manuscript offers a reanalysis of all available nuclear genomic data published on medusozoans. It represents a well though, and timely review of the available data, systematically comparing genomic features (repeated elements, intro/exon/gene size and numbers, chromosome numbers...) and genomic assemblies (available data, assembly quality and size…) in the different medusozoan classes. It largely confirms

the results obtained from analysis of single species. It also provides useful guidelines for future standardization of genomic projects focused on medusozoans."

Minor comments and suggested corrections:

1. Line 118: How was "compiled all genomic and HTS metadata reference in this review", manually? If not, please provide the scripts used for this task."

The information was collected by a combination of automatic and manual retrieval, as it was superficially mentioned in the first paragraph of the Material and Methods section. We added a few sentences to clarify this point as follows below. All of the command lines used for these analyses were originally specified in the Supplementary S7 of the original submission but this was not properly indicated in the material and methods section. We corrected this issue by adding a sentence in the corresponding section as indicated below (note: this required re-numbering the supplementary files so Supplementary file S7 is now S2).

First, we clarified the automatic and manual retrieval on line 91:

> Our main source of genomic information and metadata was NCBI Genome (Assembly, Genomes, Nucleotide, Taxonomy and SRA; [27]). We retrieved data automatically using entrez-direct v.13.9 and NCBI datasets v. 12.12. For information not present in NCBI, we checked published articles for proper information collection, as well as personal repositories mentioned in the associated articles.

We clarified that the merging of manually and automatically retrieved information was merged/compiled manually, and specified the supplementary material where scripts and command lines were deposited on line 119:

> We manually compiled all genomic information and HTS metadata referenced in this review using a report model based on previous works and public databases such as NCBI (Supplementary file S1; [29,41,42]). The command line used for retrieving genetic information and metadata, for statistics calculation and the code used for graph generation are available at Supplementary file S2 and S3.

"2. Line 236: correct contente"

This error was corrected.

"3. Line 326: The sentence starting with "Moreover, even…" is unclear. Please clarify or delete."

To clarify this point we deleted the original sentence and added the following on line 403:

> In addition, submission to the large databases like SRA and GenBank can lead to the automatic detection of specific issues such as contamination or annotation errors that might otherwise not be detected.

"4. Line 389: correct "proyects""

This error was corrected.

"5. Figure 1: it would useful to indicate in this figure genome sizes calculated from genomic assemblies, in addition to genome sizes calculated from flow cytometry and feulgen densitometry estimations; either as a new column or using another color in C"

We prefer to maintain the original version of the figure. The following reasons were considered for not adding “assembly length” in figure 1 (now renumbered as Figure 2):

- Assembly length would not be a robust estimation of genome size because different causes can lead to biased results, especially for short reads projects. High heterozygosity and incomplete collapsing of haplotypes can lead to genome size overestimation. Sequencing bias, as well as repetitive DNA misassembly, can lead to underestimations of genome size (see https://doi.org/10.1371/journal.pone.0062856; 10.1111/1755-0998.12933; https://doi.org/10.1101/2021.04.09.438957; for further details)
- Adding this information in Figure 1 (now renumbered as Figure 2) could hinder visualization as already many variables are being simultaneously plotted.
- Distribution of assembly length was specified in Figure 2a (now renumbered as Figure 3a).

"6. SM_Table2: Suplementary Material S2 - Table S1 - please correct in the title "condidering"."

This error was corrected.

Reviewer 3

"Santander et al. review the state of genome assemblies and cytogenetics of Medusozoa. This review captures the progression of the sequencing efforts in the past decade and how the field is moving with new technological advances. From their assessment of the literature and unpublished data, they found that a weakness in their community is a general lack of standardization in analysis and limited availability of intermediate assembly components, such as the repeat libraries, and associated metadata. In the end they provide recommendations for standards to be applied to ongoing and future genomic projects.

1. I felt that these recommendations fell short of extending beyond basic requirements of publishing genomes today. While these recommendations are in line with recommendations of other genomic consortia (Vertebrate Genomes Project [Rhieet al. 2021, Nature], Sanger/Moore Aquatic Symbiosis Genomics, etc.) and most publishers including GigaScience (deposit data, reproducible methods, code availability statements, etc), they are quite general. I was left wondering if this was a commentary on the whole field of genomics. "

Reviewer #1 had a very similar comment. We have added the following, which acknowledges that some of our recommendations are general to all genome projects and provides justification for why it is important to include these in this review on lines 422:

The following are suggestions to enhance genome projects and outcomes, and to promote open and collaborative research. These suggestions can be broadly applied to any genome project and are in line with those proposed by many initiatives and consortia (e.g. [33,100,101]). Nevertheless, it is worth reinforcing and discussing them in the context of this review since genome projects are more and more often being initiated in research laboratories that have historically been more focused on other aspects of medusozoan biology and may not be as familiar with these general practices:

"2. To that end, are there specific recommendations regarding medusozoans that would enhance data usage community wide that could be stated here? "

As a response to point, which was also raised by reviewer #1 we added several sentences and paragraphs. Specifically, the manuscript now includes a discussion of how curational steps on database metadata could enhance data usage. It also includes a discussion about the lack of taxon-specific databases appropriate for Medusozoa, which may inspire such an effort in the near future. In addition, our recommendation that conversations regarding the state of medusozoan genomics take place at taxon-specific meetings should lead to enhanced data usage.

On line 431:

Frequently, data and metadata that are described in the original articles or deposited in repositories are not submitted to public databases. Tracking information from multiple sources is time consuming and prone to error. Databases and repositories enable the improvement of metadata after the initial releases, by the addition of new or corrected information (e.g. publication information) from the authors. We believe that this kind of data curation would improve the state of Medusozoa genomics not only by enabling downstream analysis after the publication, but also enabling the detection of methodological options (e.g. tissue selection; sequencing technology) that would improve the quality of the results.

On line 446:

A Medusozoa-centric database with long-term maintenance is still lacking for the community (e.g. Mollusca clade [94]); but many open repositories can serve this purpose with low or no costs considering the size of the aforementioned outputs.

On line 466:

7. Engage in community-driven conversations about standards, guidelines and species priorities. There are a number of taxon-specific meetings that would be appropriate venues to engage in these conversations including the International Conference on Coelenterate Biology (~decennial; [106]), the International Jellyfish Blooms Symposium (~triennial), Cnidofest (~biennial; [107]), Tutzing workshop (~biennial; [108]), and Cnidofest zoom seminar series. In addition, satellite meetings at larger annual meetings (e.g. the Society for Integrative and Comparative Biology (SICB) or

> the Global Invertebrate Genomics Alliance (GIGA [101])) could provide appropriate venues to facilitate discussions on how the community can best move forward as more and more genomic data come online.

We also provided a link in the data availability statement to the online version of the Supplementary file 1 in Figshare. This table will be maintained and can be modified/corrected if authors from the original papers contact us. On line 522:

> A copy of table S1 will be available upon publication [114] and can be updated upon the original author's request.

"3. Are there established assembly pipelines (i.e. tools that provide the highest quality assemblies from various species) or types of sequencing effort (i.e. long read + HiC maps, transcriptome-informed gene annotation) that should be endorsed as part of your assessment?"

A rigorous assessment of this issue was not possible because Medusozoa genomic datasets are quite heterogeneous (time-scales, technologies, objectives, methods and output quality; all with a small sampling). However, it is a highly relevant topic, and we opted to mention general trends in the main text with a proper citation to more specific bibliography on methods. We added the following paragraph on line 237:

> Differences in sequencing strategy and platforms are expected to be linked with assembly quality, both in terms of continuity and completeness. For example, hybrid sequencing plus optical maps and combined evidence-based annotation should generate better results than a short-read sequencing and single-evidence annotation [61,62]. Although this general trend was observed in this review, with most Illumina-only datasets showing lower BGP-metric (Figure 3) and lower completeness (Figure 4), it is not a granted condition. Some punctual cases can exemplify biological and methodological issues that impose limitations to genome sequencing and assembly: e.g. the difficulty in obtaining chromosome-scale assemblies despite small genome sizes and combined sequencing strategies (Hi-C + short reads+ long reads) [63,64] or the difficulty in extracting high-molecular-weight DNA [20]. Because of the heterogeneity of Medusozoa genomic projects in terms of time periods, objectives, methods and resources, a proper quantitative analysis of the relationship between methods and outcome quality would not be feasible, and we prefer to refer to articles specialized in assessing methods (e.g. [61,62]).

"4. Are there specific taxonomic gaps that should be prioritized (starting Line 238)?"

There are taxonomic gaps in Medusozoa genomics that were mentioned in the **"Genomic projects: whos and hows of Medusozoa"** section. But we believe criteria for priority should come from community discussions as was carried on by other projects. To remark the importance of filling taxonomic gaps, we added the following sentences on line 466:

> 7. Engage in community-driven conversations about standards, guidelines and species priorities.

And on line 501:

> The distribution of genetic and genomic information presented significant taxonomic gaps in Medusozoa. It is a reasonable scenario since genomic sequencing data is accumulating in many medusozoan lineages. Even so, some of the most species-rich clades with a diverse array of phenotypic and ecological traits have not yet had their genomes sequenced (e.g. Scyphozoa:Coronamedusae, Hydrozoa:Macrocolonia). These, and other, heretofore genomically underexplored lineages provide golden opportunities from which to make major contributions to understanding the evolution of Medusozoa genomes and would be a wonderful contribution to the rest of the Medusozoa research community. Defining candidate species for sequencing can avoid unnecessary doubled efforts. Different international projects recognized this situation and proposed a set of criteria for prioritizing species at other scales, such as the GIGA ([101]).

"5. The majority of the resources you identified only have short-read Illumina data which inevitably means that chromosome-scale assemblies are not possible yet. However, these assemblies are sufficient for gene model comparisons across species (starting on Line 187). Is there a way to standardize gene prediction for cases where short reads may be all that is available?
Re-analysis of gene predictions with different tools may lead to varying estimates and can lead to erroneous orthology assignments (see https://doi.org/10.1111/jpy.12947, https://doi.org/10.1371/journal.pbio.3000862, and https://www.biorxiv.org/content/10.1101/2022.01.13.476251v1). Re-analysis of Rhopilema gene content using different tools increases gene predictions closer to the median gene count you've found."

Based on this commentary, we have added several sentences to clarify the problem of comparative analysis based on heterogeneous annotations. This point was explored in the section “The state of Medusozoa genomics: inner and derived knowledge” in relation to articles' conclusions about lineage-specific genes and increases/decreases in gene content. Moreover, this point was also recapitulated at the final part of the recommendations, reinforcing the problem of comparative analysis.

We made the following additions on line 314:

> Recent evidence proved that the detection of lineage-specific genes, and other analyses relying on accurate annotation and orthology prediction, can be significantly biased by methodological artifacts [79–83]; several problems have been identified, such as low taxon sampling, heterogeneous gene predictions, and failure of detecting distant homology and fast-evolving orthologues. These considerations are highly relevant in Medusozoa, as comparisons are often made, by necessity, with distantly related species (e.g. Anthozoa has been estimated to have diverged from Medusozoa around 800 million years ago [84]).

On line 460:

> The latter suggestions (3-6) are mainly related to providing detailed methodologies of bioinformatic analyses. First, proper method and results descriptions can help to recover metadata and criteria usually not available in large sequence repositories. Second, comparative analyses depend upon standardization at different levels and significant sample sizes. The inclusion of species in downstream analyses is limited by data availability and proper description of previous analyses, custom software and results.

and on line 475:

> The adoption of best practices in the Medusozoa genomics community will pave the way for major breakthroughs regarding understanding the genomic basis for several evolutionary innovations that arose within and in the stem lineage of Medusozoa. Similar advances were achieved with extensive taxon sampling at broader scales, where 25 novel core gene groups enriched in regulatory functions might be underlying the emergence of animals [109,110]. Medusozoa innovations have puzzled the community for decades [5,7,11,111] and include the origin of the medusa, the loss of polyp structures, the establishment of symbiosis, the blooming potential, and the evolution of an extremely potent venom. A deeper understanding of the genomic events driving these innovations will require accurate identifications of a number of key genomic features including (but not limited to) single copy orthologs, gene losses, lineage-specific genes, gene family expansions and non-coding regulatory sequences.

In relation to the question: **"Is there a way to standardize gene prediction for cases where short reads may be all that is available?"**

We are not aware of any pipeline specifically designed to standardize gene prediction for short-read assemblies. One solution would be to re-annotate and annotate all genomes by the same methodology. Another solution would be to use existing annotations and improve them by comparative analysis or by targeting specific gene families of interest. These considerations were added to “Prospects on genomic data and general resources" but not as part of the final recommendations on line 390.

> An alternative solution for comprehensive comparative analyses is to (re)annotate all genomes with the same pipeline, a task that is laborious and time consuming. Some programs were designed for achieving this task simultaneously in many related species (e.g. [89,90]). Another alternative is to use specific software developed to improve genome annotations by leveraging data from multiple species (e.g. [91,92]) or targeting specific gene families [93,94]. Finally, differences in annotation due to methodological artifacts can be accommodated in comparative analysis if considered as a variable in the statistical tests (e.g. comparing tRNA genes in high and low quality avian genomes [95]).

"6. Regarding the recommendation for depositing intermediates into repositories (#3), is there one established for the community or are you referring to more general ones like Dryad, FigShare, Repbase, etc.? Providing an example genome project or two that shares these associated files might be helpful."

We were referring to general repositories. We have clarified this point in the section titled: "Deposit output results that were fundamental in any of the steps of the analysis" on line 446:

> A Medusozoa-centric database with long-term maintenance is still lacking for the community (e.g. Mollusca clade [104]); but many open repositories can serve this purpose with low or no costs considering the size of the aforementioned outputs. There are open topic-centric repositories (e.g Dfam [105] for repetitive DNA), general repositories (e.g. FigShare, Zenodo; or even NCBI for annotation tracks) as well as personal or institutional ones. Many of the reviewed genomic projects already made use of these repositories but failed to deposit some of the outputs. A solution for this inconvenience is to update submissions or create novel ones (e.g. submit annotations to NCBI or ENA) to deposit the missing outputs.

"7. There can be cost associated with hosting these resources. Do you see that as a barrier to researchers providing this sort of data?"

Although repositories can be expensive, the intermediates we mentioned in recommendation #3 (gene and repetitive models and tracks) are frequently below 1gb. These file sizes can be easily accommodated by repositories with no cost at all. Therefore, we do not find cost to be a barrier for deposit. One possible barrier is that in general the submission process is cumbersome, something that might improve as new workflows are developed (as mentioned in the final conclusions of the manuscript).

"8. A recommendation that is provided earlier in the paper is the call for lineage-specific single copy ortholog sets (Line 228). Should this be re-stated in the final recommendations as well?"

The determination of a single copy ortholog set for Medusozoa would depend on the availability of gene annotations for several species, the completeness of these annotationes, or availability of sufficient information enabling re-annotation of these genomes. We believe this might not be possible yet in Medusozoa, therefore this topic was restated together with suggestion #5 (starting on line 480).

"Minor Comments:"
"9. Line 31-33: This sentence seems to be constructed of two thoughts but missing a connector between them."

This error was corrected as follows in the abstract:

> Modern genomic DNA sequencing in this group started in 2010 with the publishing of the Hydra vulgaris genome **"and"** has experienced an exponential increase in the past three years.

"The following corrections were also done:"
"Line 98: … assembly statistics using the statswrapper.sh script …"

"Line 169: … [55], and the …"
"Line 315: Remove "of" between reusing and previously."
"Line 337: "reran" should be "rerun"."
"Line 389: Typo, "projects""

"10. Figures: The resolution of the figures provided made it difficult to review. Specifically Figure 3 was quite pixelated."

The figures are concordant with the journal’s requirements. The low quality of figures might be due to compression before the journal sent them to the reviewers. High quality versions of each version can be downloaded from the link available next to the figures in the pdf or svg files in Supplementary file S9. Leaving aside, Figure 2 and 3 (now re-numbered as Figure 3 and 4) were corrected to improve visualization; font size was increased and graph legend was repositioned.
