# Supplementary figures and images for: The state of Medusozoa genomics: current evidence and future challenges
Source: Gigascience. 2022 May 17;11:giac036. doi: 10.1093/gigascience/giac036 (PMC9112765; doi:10.1093/gigascience/giac036)

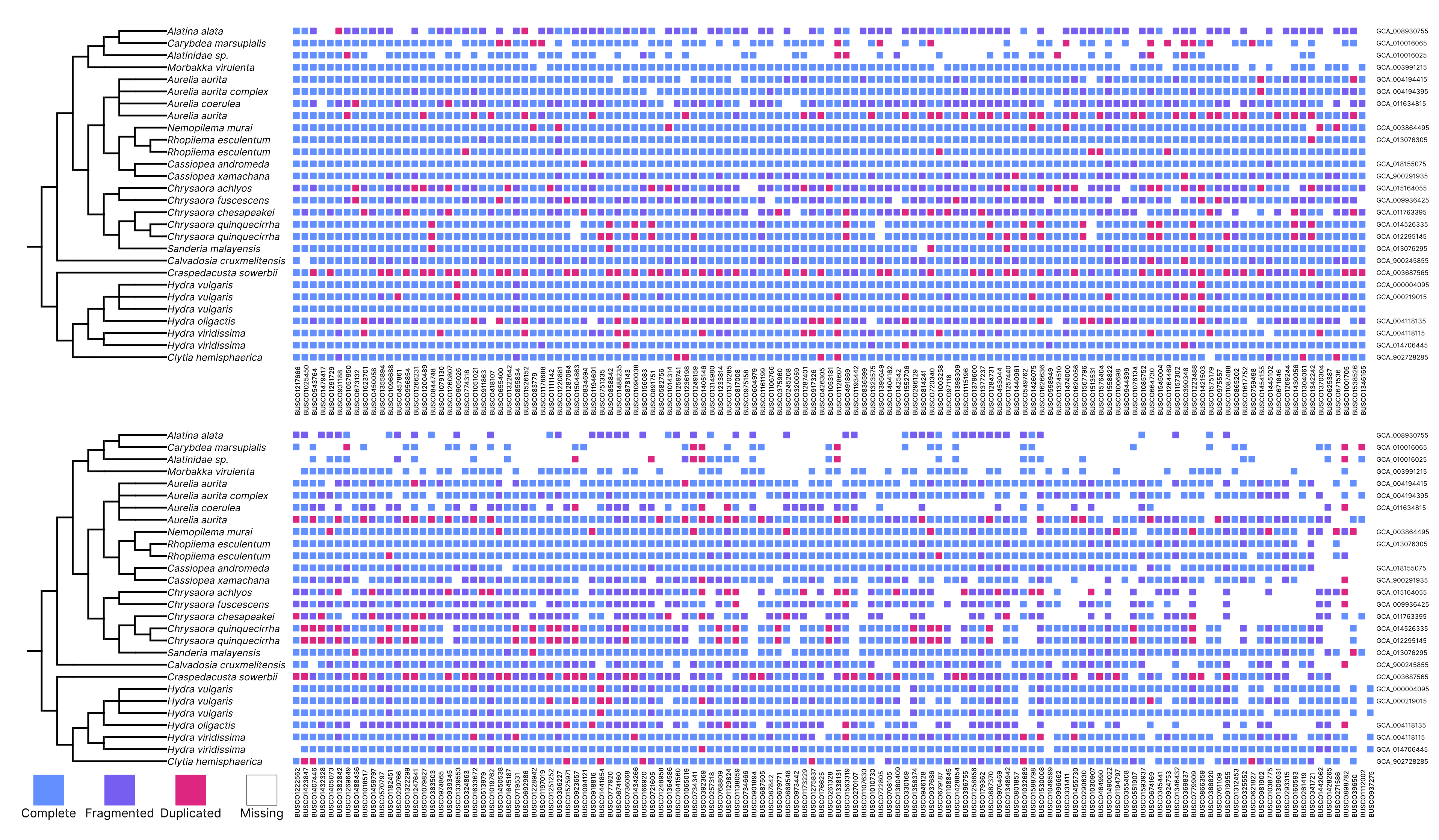

Supplement: giac036_Supplemental_Files [file giac036_supplemental_files.zip › Supplementary_file_S6_Figure_S1_EukaryotaBUSCO.png]
