# Supplementary material for: The state of Medusozoa genomics: current evidence and future challenges
Source: Gigascience. 2022 May 17;11:giac036. doi: 10.1093/gigascience/giac036 (PMC9112765; doi:10.1093/gigascience/giac036)
Supplement: giac036_Response_to_Reviewer_Comments_Original_Submission [file giac036_response_to_reviewer_comments_original_submission.pdf]

Author's Response To Reviewer Comments

Close

Editor

"Your manuscript "The state of Medusozoa genomics: past evidence and future challenges" (Review Article; GIGA-D-21-00404) has been assessed by three reviewers. Based on these reports, I am pleased to inform you that it is potentially acceptable for publication in GigaScience, once you have carried out some essential revisions suggested by our reviewers. Their reports are below.
I'd like to highlight three points:"

We are very appreciative of the excellent suggestions from the reviewers and editor. We have done our best to address each point and we feel that the manuscript has been greatly improved as a result of the review process. Thank you for the time dedicated to our manuscript. We provide a point-by-point answer to each suggestion. We also provide a new main text and a copy of the original text with all the changes kept as tracks. Line numbers in this letter are referenced to the new main text file in de submission PDF. Original comments made by the editor and the reviewers are indicated in bold or between quotation marks. We also provide a formated copy of the response to the reviewers as a separate file at the end of the submission PDF.

"1. Two of the reviewers mention that the "recommendations" would benefit if it would make clearer if there are any Medusozoa-specific recommendations (in addition to advice that is generally applicable to all animal genome projects)"

We have added the following to address this point generally on line 422:

The following are suggestions to enhance genome projects and outcomes, and to promote open and collaborative research. These suggestions can be broadly applied to any genome project and are in line with those proposed by many initiatives and consortia (e.g. [33,100,101]). Nevertheless, it is worth reinforcing and discussing them in the context of this review since genome projects are more and more often being initiated in research laboratories that have historically been more focused on other aspects of medusozoan biology and may not be as familiar with these general practices:

We have added the following to point #3 that refers to where to deposit data on lines 446:

A Medusozoa-centric database with long-term maintenance is still lacking for the community (e.g. Mollusca clade [104]); but many open repositories can serve this purpose with low or no costs considering the size of the aforementioned outputs. There are open topic-centric repositories (e.g Dfam [105] for repetitive DNA), general repositories (e.g. FigShare, Zenodo; or even NCBI for annotation tracks) as well as personal or institutional ones. Many of the reviewed genomic projects already made use of these repositories but failed to deposit some of the outputs. A solution for this inconvenience is to update submissions or create novel ones (e.g. submit annotations to NCBI or ENA) to deposit the missing outputs.

"2. Reviewer 1 recommends to make your code public, and I strongly support this, as it is also in line with our journal guidelines. You can also host code and supporting data in our repository GigaDB - our data curators will be happy to help. Please attach an open (OSI-compliant) licence to any scripts/code. (https://opensource.org/licenses)"

All the command lines used in this work were originally specified in the Supplementary File S7 of the original submission (Supplementary File S2 in the current version) but it was not properly indicated in the material and methods section. We corrected this issue by adding the following sentence on lines 122:

The command line used for retrieving genetic information and metadata, for statistics calculation and the

code used for graph generation are available at Supplementary file S2 and S3.

We have also added the scripts used for constructing graphs in Supplementary file S3 (as suggested by reviewer 1). All the software used in this work is open and was properly referenced.

We deposited all supplementary files in Figshare and GigaDB and included a statement of open license to scripts on lines 518:

Data availability
All collected information, outputs and scripts supporting new results are available in the supplementary files S1-S9 in Figshare [114] and in GigaDB [115].

"3. Although not mentioned by the reviewers, I feel your manuscript would be more interesting for readers from outside the medusozoa community if you explained in a bit more detail the actual biological questions that have been addressed with these genomes; such as toxins, metazoan evolution / body plan evolution, Hox genes, immunity, etc.. These topics are mentioned in the introduction, but I feel they could be picked up again in a bit more detail in the discussion, to illustrate the biological insights gained from the genome projects."

We have added two paragraphs that highlight the insight genome projects bring understanding medusozoa biology.

Starting on line 301:

The complex nature of Medusozoa venom has been investigated by a number of transcriptomic, proteomic and genomic studies (reviewed in [26]). Several putative toxin genes and domains have been identified, covering a significant part of the wide range of known toxins [20,22,59,73]. In Scyphozoa, toxin-like genes were often recovered as multicopy sets [20,59]. Moreover, in R. esculentum toxin-like genes were also tandemly arranged and several of them were located nearby in chromosome 7, suggesting that the observed organization might influence toxin co-expression[59]. Minicollagens, which are major components of nematocysts, also had a clustered organization and a pattern of co-expression in Aurelia [20]. These examples add to various clustered genes described in Cubozoa, Hydrozoa and Anthozoa, and would indicate that gene clustering and operon-like expression of toxin genes is widespread in Cnidaria ([20] and references therein).

and starting on line 329:

The complex life cycle of Medusozoa has resulted from the combination of both ancestral and novel features. Aurelia, Morbakka virulenta and Clytia hemisphaerica have significantly different patterns of gene expression across stages and during transitions [19–21]. Differentially expressed genes include many conserved ancestral families of transcription factors [19–21]; there is also a considerable amount of the putative lineage-restricted genes that show differential expression in the adult stages [20,21]. A few of these “novel” medusozoan genes have been described, such as novel myosin-tail proteins that are absent from Anthozoa and represent markers of the medusae striated muscles [20]. It was suggested that the evolution of the Medusozoa complex life cycle would therefore have involved the rewiring of regulatory pathways of ancestral genes and the contribution of new ones [19–21]. As such, the body plan and life cycle simplifications observed in Clytia and Hydra, respectively, would be the result of loss of transcription factors involved in their development [21]. Finally, the significance of many of the putative Medusozoa and species-specific genes remain to be elucidated.

"4. For a review article, please also feel free to add illustrations/photos of relevant medusozoa species, if you wish (but please check with any copyright holder, if applicable - images will be published under an open cc-by licence)."

We added a new figure (Figure 1) with photographs of example species of each Medusozoa class. Some photographs (Figure 1 A, B, D, E) were recovered from an online open database called Cifonauta, available under open cc-by license, and it was properly cited. The remaining photographs were provided by Marta Chiodin (Figure 1C), Joseph Ryan (co-author; Figure 1 F, G), with permission to publish under CC-BY license. As a result of the addition of a new Figure 1, all figures were renumbered accordingly.

Reviewer 1
"In this paper, Santander et al. review the field of medusozoan genomics, which has burgeoned in the

last three or so years. Overall, I found this a clear, interesting read. The manuscript is well-written, the figures are valuable, and the authors nicely describe the history of the research as well as the state of the field. The findings are not monumental, but it is a worthwhile exercise to survey the rapidly-increasing dataset of genomes in a systematic way, and this review will be a useful start for further work in medusozoan comparative genomics. I rarely suggest a paper should be accepted during the first round of review, and I usually try to provide more constructive feedback than I do here, but I really don't have much too much to quibble with. A couple thoughts are provided below:

1. The set of suggestions for future work near the end of the document are fine, but they could apply broadly to any genome project. I encourage the authors to consider whether there are specific problems related to medusozoan evolution that are hampered by inconsistencies between studies, and discuss how their recommendations (or additional ones) could help resolve them."

This comment also addresses reviewer #3's first point as well. We have added the following, which acknowledges that some of our recommendations are general to all genome projects and provides justification for why it is important to include these in this review on line 422:

The following are suggestions to enhance genome projects and outcomes, and to promote open and collaborative research. These suggestions can be broadly applied to any genome project and are in line with those proposed by many initiatives and consortia (e.g. [33,100,101]). Nevertheless, it is worth reinforcing and discussing them in the context of this review since genome projects are more and more often being initiated in research laboratories that have historically been more focused on other aspects of medusozoan biology and may not be as familiar with these general practices:

In the recommendation regarding depositing results in public databases we discussed its importance and how metadata can be improved when datasets were already made public on line 431:

Frequently, data and metadata that are described in the original articles or deposited in repositories are not submitted to public databases. Tracking information from multiple sources is time consuming and prone to error. Databases and repositories enable the improvement of metadata after the initial releases, by the addition of new or corrected information (e.g. publication information) from the authors. We believe that this kind of data curation would improve the state of Medusozoa genomics not only by enabling downstream analysis after the publication, but also enabling the detection of methodological options (e.g. tissue selection; sequencing technology) that would improve the quality of the results.

In the section about depositing intermediate outputs, we have added information on the state of relevant taxon-specific databases on line 446:

Medusozoa-centric database with long-term maintenance is still lacking for the community (e.g. Mollusca clade [104]); but many open repositories can serve this purpose with low or no costs considering the size of the aforementioned outputs.

We added a paragraph discussing potential problems and benefits related to proper method description on line 460.

The latter suggestions (3-6) are mainly related to providing detailed methodologies of bioinformatic analyses. First, proper method and results descriptions can help to recover metadata and criteria usually not available in large sequence repositories. Second, comparative analyses depend upon standardization at different levels and significant sample sizes. The inclusion of species in downstream analyses is limited by data availability and proper description of previous analyses, custom software and results.

We added a recommendation about engaging in community-wide discussions, and highlighted potential venues that would be appropriate for discussing medusozoan genomics standards starting on line 466:

7. Engage in community-driven conversations about standards, guidelines and species priorities. There are a number of taxon-specific meetings that would be appropriate venues to engage in these conversations including the International Conference on Coelenterate Biology (~decennial; [106]), the International Jellyfish Blooms Symposium (~triennial), Cnidofest (~biennial; [107]), Tutzing workshop (~biennial; [108]), and Cnidofest zoom seminar series. In addition, satellite meetings at larger annual meetings (e.g. the Society for Integrative and Comparative Biology (SICB) or the Global Invertebrate Genomics Alliance (GIGA [101])) could provide appropriate venues to facilitate discussions on how the community can best move forward as more and more genomic data come online.

We close the section with a paragraph that explains how adhering to standards will benefit the medusozoan community on line 475:

The adoption of best practices in the Medusozoa genomics community will pave the way for major breakthroughs regarding understanding the genomic basis for several evolutionary innovations that arose within and in the stem lineage of Medusozoa. Similar advances were achieved with extensive taxon sampling at broader scales, where 25 novel core gene groups enriched in regulatory functions might be underlying the emergence of animals [109,110]. Medusozoa innovations have puzzled the community for decades [5,7,11,111] and include the origin of the medusa, the loss of polyp structures, the establishment of symbiosis, the blooming potential, and the evolution of an extremely potent venom. A deeper understanding of the genomic events driving these innovations will require accurate identifications of a number of key genomic features including (but not limited to) single copy orthologs, gene losses, lineage-specific genes, gene family expansions and non-coding regulatory sequences.

Related to this last point, we also suggest to read the added sentences after reviewer #3 comment on line 314:

Recent evidence proved that the detection of lineage-specific genes, and other analyses relying on accurate annotation and orthology prediction, can be significantly biased by methodological artifacts [79–83]; several problems have been identified, such as low taxon sampling, heterogeneous gene predictions, and failure of detecting distant homology and fast-evolving orthologues. These considerations are highly relevant in Medusozoa, as comparisons are often made, by necessity, with distantly related species (e.g. Anthozoa has been estimated to have diverged from Medusozoa around 800 million years ago [84]).

"2. I would encourage the authors to practice what they preach in terms of transparency, and make the code they used in their methods public (e.g. statswrapper.sh, AGAT, BUSCO, ETE Toolkit, Matplotlib, Seaborn). The code does not need to be executable, but a supplemental text and/or repository with as much of the starting data and commands executed as possible would make it easier for others to replicate this work and apply it to future comparative genomics projects."

All the command line used in this work was originally specified in the Supplementary S7 of the original submission but we did not not properly indicate this in the material and methods section. We corrected this issue by adding a sentence in the corresponding section as indicated below (note: this required re-numbering the supplementary files so Supplementary file S7 is now S2). We also included the scripts used for constructing graphs. All the packages and softwares used in the command line and in the custom scripts (statswrapper.sh, AGAT, BUSCO, ETE Toolkit, Matplotlib, Seaborn) are open. We have added the following on line 122:

The command line used for retrieving genetic information and metadata, for statistics calculation and the code used for graph generation are available at Supplementary file S2 and S3.

"3. Line 236: "…ploidy level, heterochromatin contente." This should be changed to "…ploidy level, and heterochromatin content.""

This error was corrected.

"4. Line 253-254: "…evolution of genome size is a long-standing question that is included in the so-called C-value Enigma [40]." The authors provide a citation, but I think this sentence would be stronger with a brief explanation of what the C- value Enigma is. Medusozoans are a great example of this "enigma", so it's worth reinforcing."

We have added the following to clarify the C-value enigma on line 274:

… "C-value Enigma" [41]. This name stems from the difficulty elucidating the evolutionary forces (e.g. drift and natural selection) that have given rise and serve to maintain variations in genome size, the mechanisms of genome size change, and the consequences of these variations at an organismal level [41]. Several conflicting hypotheses have been postulated to explain this puzzle with most having experimental support in some but not all lineages (reviewed in [68]).

Reviewer 2

"This manuscript offers a reanalysis of all available nuclear genomic data published on medusozoans. It represents a well though, and timely review of the available data, systematically comparing genomic features (repeated elements, intro/exon/gene size and numbers, chromosome numbers...) and genomic assemblies (available data, assembly quality and size…) in the different medusozoan classes. It largely confirms the results obtained from analysis of single species. It also provides useful guidelines for future standardization of genomic projects focused on medusozoans."
Minor comments and suggested corrections:
1. Line 118: How was "compiled all genomic and HTS metadata reference in this review", manually? If not, please provide the scripts used for this task."

The information was collected by a combination of automatic and manual retrieval, as it was superficially mentioned in the first paragraph of the Material and Methods section. We added a few sentences to clarify this point as follows below. All of the command lines used for these analyses were originally specified in the Supplementary S7 of the original submission but this was not properly indicated in the material and methods section. We corrected this issue by adding a sentence in the corresponding section as indicated below (note: this required re-numbering the supplementary files so Supplementary file S7 is now S2).

First, we clarified the automatic and manual retrieval on line 91:
Our main source of genomic information and metadata was NCBI Genome (Assembly, Genomes, Nucleotide, Taxonomy and SRA; [27]). We retrieved data automatically using entrez-direct v.13.9 and NCBI datasets v. 12.12. For information not present in NCBI, we checked published articles for proper information collection, as well as personal repositories mentioned in the associated articles.
We clarified that the merging of manually and automatically retrieved information was merged/compiled manually, and specified the supplementary material where scripts and command lines were deposited on line 119:
We manually compiled all genomic information and HTS metadata referenced in this review using a report model based on previous works and public databases such as NCBI (Supplementary file S1; [29,41,42]). The command line used for retrieving genetic information and metadata, for statistics calculation and the code used for graph generation are available at Supplementary file S2 and S3.

"2. Line 236: correct contente"

This error was corrected.

"3. Line 326: The sentence starting with "Moreover, even…" is unclear. Please clarify or delete."

To clarify this point we deleted the original sentence and added the following on line 403:
In addition, submission to the large databases like SRA and GenBank can lead to the automatic detection of specific issues such as contamination or annotation errors that might otherwise not be detected.

"4. Line 389: correct "proyects""

This error was corrected.

"5. Figure 1: it would useful to indicate in this figure genome sizes calculated from genomic assemblies, in addition to genome sizes calculated from flow cytometry and feulgen densitometry estimations; either as a new column or using another color in C"

We prefer to maintain the original version of the figure. The following reasons were considered for not adding "assembly length" in figure 1 (now renumbered as Figure 2):

- Assembly length would not be a robust estimation of genome size because different causes can lead to biased results, especially for short reads projects. High heterozygosity and incomplete collapsing of haplotypes can lead to genome size overestimation. Sequencing bias, as well as repetitive DNA misassembly, can lead to underestimations of genome size (see https://doi.org/10.1371/journal.pone.0062856; 10.1111/1755-0998.12933; https://doi.org/10.1101/2021.04.09.438957; for further details)
- Adding this information in Figure 1 (now renumbered as Figure 2) could hinder visualization as already many variables are being simultaneously plotted.

• Distribution of assembly length was specified in Figure 2a (now renumbered as Figure 3a).

"6. SM_Table2: Suplementary Material S2 - Table S1 - please correct in the title "condidering"."

This error was corrected.

Reviewer 3
"Santander et al. review the state of genome assemblies and cytogenetics of Medusozoa. This review captures the progression of the sequencing efforts in the past decade and how the field is moving with new technological advances. From their assessment of the literature and unpublished data, they found that a weakness in their community is a general lack of standardization in analysis and limited availability of intermediate assembly components, such as the repeat libraries, and associated metadata. In the end they provide recommendations for standards to be applied to ongoing and future genomic projects.

1. I felt that these recommendations fell short of extending beyond basic requirements of publishing genomes today. While these recommendations are in line with recommendations of other genomic consortia (Vertebrate Genomes Project [Rhieet al. 2021, Nature], Sanger/Moore Aquatic Symbiosis Genomics, etc.) and most publishers including GigaScience (deposit data, reproducible methods, code availability statements, etc), they are quite general. I was left wondering if this was a commentary on the whole field of genomics. "

Reviewer #1 had a very similar comment. We have added the following, which acknowledges that some of our recommendations are general to all genome projects and provides justification for why it is important to include these in this review on lines 422:

The following are suggestions to enhance genome projects and outcomes, and to promote open and collaborative research. These suggestions can be broadly applied to any genome project and are in line with those proposed by many initiatives and consortia (e.g. [33,100,101]). Nevertheless, it is worth reinforcing and discussing them in the context of this review since genome projects are more and more often being initiated in research laboratories that have historically been more focused on other aspects of medusozoan biology and may not be as familiar with these general practices:

"2. To that end, are there specific recommendations regarding medusozoans that would enhance data usage community wide that could be stated here? "

As a response to point, which was also raised by reviewer #1 we added several sentences and paragraphs. Specifically, the manuscript now includes a discussion of how curational steps on database metadata could enhance data usage. It also includes a discussion about the lack of taxon-specific databases appropriate for Medusozoa, which may inspire such an effort in the near future. In addition, our recommendation that conversations regarding the state of medusozoan genomics take place at taxon-specific meetings should lead to enhanced data usage.

On line 431:

Frequently, data and metadata that are described in the original articles or deposited in repositories are not submitted to public databases. Tracking information from multiple sources is time consuming and prone to error. Databases and repositories enable the improvement of metadata after the initial releases, by the addition of new or corrected information (e.g. publication information) from the authors. We believe that this kind of data curation would improve the state of Medusozoa genomics not only by enabling downstream analysis after the publication, but also enabling the detection of methodological options (e.g. tissue selection; sequencing technology) that would improve the quality of the results.

On line 446:

A Medusozoa-centric database with long-term maintenance is still lacking for the community (e.g. Mollusca clade [94]); but many open repositories can serve this purpose with low or no costs considering the size of the aforementioned outputs.

On line 466:

7. Engage in community-driven conversations about standards, guidelines and species priorities. There

are a number of taxon-specific meetings that would be appropriate venues to engage in these conversations including the International Conference on Coelenterate Biology (~decennial; [106]), the International Jellyfish Blooms Symposium (~triennial), Cnidofest (~biennial; [107]), Tutzing workshop (~biennial; [108]), and Cnidofest zoom seminar series. In addition, satellite meetings at larger annual meetings (e.g. the Society for Integrative and Comparative Biology (SICB) or the Global Invertebrate Genomics Alliance (GIGA [101])) could provide appropriate venues to facilitate discussions on how the community can best move forward as more and more genomic data come online.

We also provided a link in the data availability statement to the online version of the Supplementary file 1 in Figshare. This table will be maintained and can be modified/corrected if authors from the original papers contact us. On line 522:

A copy of table S1 will be available upon publication [114] and can be updated upon the original author's request.

"3. Are there established assembly pipelines (i.e. tools that provide the highest quality assemblies from various species) or types of sequencing effort (i.e. long read + HiC maps, transcriptome-informed gene annotation) that should be endorsed as part of your assessment?"
A rigorous assessment of this issue was not possible because Medusozoa genomic datasets are quite heterogeneous (time-scales, technologies, objectives, methods and output quality; all with a small sampling). However, it is a highly relevant topic, and we opted to mention general trends in the main text with a proper citation to more specific bibliography on methods. We added the following paragraph on line 237:

Differences in sequencing strategy and platforms are expected to be linked with assembly quality, both in terms of continuity and completeness. For example, hybrid sequencing plus optical maps and combined evidence-based annotation should generate better results than a short-read sequencing and single-evidence annotation [61,62]. Although this general trend was observed in this review, with most Illumina-only datasets showing lower BGP-metric (Figure 3) and lower completeness (Figure 4), it is not a granted condition. Some punctual cases can exemplify biological and methodological issues that impose limitations to genome sequencing and assembly: e.g. the difficulty in obtaining chromosome-scale assemblies despite small genome sizes and combined sequencing strategies (Hi-C + short reads+ long reads) [63,64] or the difficulty in extracting high-molecular-weight DNA [20]. Because of the heterogeneity of Medusozoa genomic projects in terms of time periods, objectives, methods and resources, a proper quantitative analysis of the relationship between methods and outcome quality would not be feasible, and we prefer to refer to articles specialized in assessing methods (e.g. [61,62]).

"4. Are there specific taxonomic gaps that should be prioritized (starting Line 238)?"

There are taxonomic gaps in Medusozoa genomics that were mentioned in the "Genomic projects: whos and hows of Medusozoa" section. But we believe criteria for priority should come from community discussions as was carried on by other projects. To remark the importance of filling taxonomic gaps, we added the following sentences on line 466:

7. Engage in community-driven conversations about standards, guidelines and species priorities.

And on line 501:

The distribution of genetic and genomic information presented significant taxonomic gaps in Medusozoa. It is a reasonable scenario since genomic sequencing data is accumulating in many medusozoan lineages. Even so, some of the most species-rich clades with a diverse array of phenotypic and ecological traits have not yet had their genomes sequenced (e.g. Scyphozoa:Coronamedusae, Hydrozoa:Macrocolonia). These, and other, heretofore genomically underexplored lineages provide golden opportunities from which to make major contributions to understanding the evolution of Medusozoa genomes and would be a wonderful contribution to the rest of the Medusozoa research community. Defining candidate species for sequencing can avoid unnecessary doubled efforts. Different international projects recognized this situation and proposed a set of criteria for prioritizing species at other scales, such as the GIGA ([101]).

"5. The majority of the resources you identified only have short-read Illumina data which inevitably means that chromosome-scale assemblies are not possible yet. However, these assemblies are sufficient for gene model comparisons across species (starting on Line 187). Is there a way to standardize gene prediction for cases where short reads may be all that is available?
Re-analysis of gene predictions with different tools may lead to varying estimates and can lead to erroneous orthology assignments (see https://doi.org/10.1111/jpy.12947, https://doi.org/10.1371/journal.pbio.3000862, and https://www.biorxiv.org/content/10.1101/2022.01.13.476251v1). Re-analysis of Rhopilema gene content using different tools increases gene predictions closer to the median gene count you've found."

Based on this commentary, we have added several sentences to clarify the problem of comparative analysis based on heterogeneous annotations. This point was explored in the section "The state of Medusozoa genomics: inner and derived knowledge" in relation to articles' conclusions about lineage-specific genes and increases/decreases in gene content. Moreover, this point was also recapitulated at the final part of the recommendations, reinforcing the problem of comparative analysis.

We made the following additions on line 314:

Recent evidence proved that the detection of lineage-specific genes, and other analyses relying on accurate annotation and orthology prediction, can be significantly biased by methodological artifacts [79–83]; several problems have been identified, such as low taxon sampling, heterogeneous gene predictions, and failure of detecting distant homology and fast-evolving orthologues. These considerations are highly relevant in Medusozoa, as comparisons are often made, by necessity, with distantly related species (e.g. Anthozoa has been estimated to have diverged from Medusozoa around 800 million years ago [84]).
On line 460:
The latter suggestions (3-6) are mainly related to providing detailed methodologies of bioinformatic analyses. First, proper method and results descriptions can help to recover metadata and criteria usually not available in large sequence repositories. Second, comparative analyses depend upon standardization at different levels and significant sample sizes. The inclusion of species in downstream analyses is limited by data availability and proper description of previous analyses, custom software and results.
and on line 475:
The adoption of best practices in the Medusozoa genomics community will pave the way for major breakthroughs regarding understanding the genomic basis for several evolutionary innovations that arose within and in the stem lineage of Medusozoa. Similar advances were achieved with extensive taxon sampling at broader scales, where 25 novel core gene groups enriched in regulatory functions might be underlying the emergence of animals [109,110]. Medusozoa innovations have puzzled the community for decades [5,7,11,111] and include the origin of the medusa, the loss of polyp structures, the establishment of symbiosis, the blooming potential, and the evolution of an extremely potent venom. A deeper understanding of the genomic events driving these innovations will require accurate identifications of a number of key genomic features including (but not limited to) single copy orthologs, gene losses, lineage-specific genes, gene family expansions and non-coding regulatory sequences.
In relation to the question: "Is there a way to standardize gene prediction for cases where short reads may be all that is available?"
We are not aware of any pipeline specifically designed to standardize gene prediction for short-read assemblies. One solution would be to re-annotate and annotate all genomes by the same methodology. Another solution would be to use existing annotations and improve them by comparative analysis or by targeting specific gene families of interest. These considerations were added to "Prospects on genomic data and general resources'' but not as part of the final recommendations on line 390.
An alternative solution for comprehensive comparative analyses is to (re)annotate all genomes with the same pipeline, a task that is laborious and time consuming. Some programs were designed for achieving this task simultaneously in many related species (e.g. [89,90]). Another alternative is to use specific software developed to improve genome annotations by leveraging data from multiple species (e.g. [91,92]) or targeting specific gene families [93,94]. Finally, differences in annotation due to methodological artifacts can be accommodated in comparative analysis if considered as a variable in the statistical tests (e.g. comparing tRNA genes in high and low quality avian genomes [95]).

"6. Regarding the recommendation for depositing intermediates into repositories (#3), is there one established for the community or are you referring to more general ones like Dryad, FigShare, Repbase, etc.? Providing an example genome project or two that shares these associated files might be helpful."

We were referring to general repositories. We have clarified this point in the section titled: "Deposit

output results that were fundamental in any of the steps of the analysis" on line 446:

A Medusozoa-centric database with long-term maintenance is still lacking for the community (e.g. Mollusca clade [104]); but many open repositories can serve this purpose with low or no costs considering the size of the aforementioned outputs. There are open topic-centric repositories (e.g Dfam [105] for repetitive DNA), general repositories (e.g. FigShare, Zenodo; or even NCBI for annotation tracks) as well as personal or institutional ones. Many of the reviewed genomic projects already made use of these repositories but failed to deposit some of the outputs. A solution for this inconvenience is to update submissions or create novel ones (e.g. submit annotations to NCBI or ENA) to deposit the missing outputs.

"7. There can be cost associated with hosting these resources. Do you see that as a barrier to researchers providing this sort of data?"

Although repositories can be expensive, the intermediates we mentioned in recommendation #3 (gene and repetitive models and tracks) are frequently below 1gb. These file sizes can be easily accommodated by repositories with no cost at all. Therefore, we do not find cost to be a barrier for deposit. One possible barrier is that in general the submission process is cumbersome, something that might improve as new workflows are developed (as mentioned in the final conclusions of the manuscript).

"8. A recommendation that is provided earlier in the paper is the call for lineage-specific single copy ortholog sets (Line 228). Should this be re-stated in the final recommendations as well?"

The determination of a single copy ortholog set for Medusozoa would depend on the availability of gene annotations for several species, the completeness of these annotationes, or availability of sufficient information enabling re-annotation of these genomes. We believe this might not be possible yet in Medusozoa, therefore this topic was restated together with suggestion #5 (starting on line 480).

"Minor Comments:"
"9. Line 31-33: This sentence seems to be constructed of two thoughts but missing a connector between them."

This error was corrected as follows in the abstract:

Modern genomic DNA sequencing in this group started in 2010 with the publishing of the Hydra vulgaris genome "and" has experienced an exponential increase in the past three years.

"The following corrections were also done:"
"Line 98: … assembly statistics using the statswrapper.sh script …"
"Line 169: … [55], and the …"
"Line 315: Remove "of" between reusing and previously."
"Line 337: "reran" should be "rerun"."
"Line 389: Typo, "projects""

"10. Figures: The resolution of the figures provided made it difficult to review. Specifically Figure 3 was quite pixelated."

The figures are concordant with the journal’s requirements. The low quality of figures might be due to compression before the journal sent them to the reviewers. High quality versions of each version can be downloaded from the link available next to the figures in the pdf or svg files in Supplementary file S9. Leaving aside, Figure 2 and 3 (now re-numbered as Figure 3 and 4) were corrected to improve visualization; font size was increased and graph legend was repositioned.

Close
