# Supplementary material for: The state of Medusozoa genomics: current evidence and future challenges
Source: Gigascience. 2022 May 17;11:giac036. doi: 10.1093/gigascience/giac036 (PMC9112765; doi:10.1093/gigascience/giac036)
Supplement: giac036_Reviewer_1_Report_Original_Submission — David A. Gold -- 1/9/2022 Reviewed [file giac036_reviewer_1_report_original_submission.pdf]

Reviewer Report

Title: The state of Medusozoa genomics: current evidence and future challenges

Version: Original Submission **Date:** 1/9/2022

Reviewer name: David A. Gold

Reviewer Comments to Author:

In this paper, Santander et al. review the field of medusozoan genomics, which has burgeoned in the last three or so years. Overall, I found this a clear, interesting read. The manuscript is well-written, the figures are valuable, and the authors nicely describe the history of the research as well as the state of the field. The findings are not monumental, but it is a worthwhile exercise to survey the rapidly-increasing dataset of genomes in a systematic way, and this review will be a useful start for further work in medusozoan comparative genomics. I rarely suggest a paper should be accepted during the first round of review, and I usually try to provide more constructive feedback than I do here, but I really don't have much too much to quibble with. A couple thoughts are provided below:
1) The set of suggestions for future work near the end of the document are fine, but they could apply broadly to any genome project. I encourage the authors to consider whether there are specific problems related to medusozoan evolution that are hampered by inconsistencies between studies, and discuss how their recommendations (or additional ones) could help resolve them.
2) I would encourage the authors to practice what they preach in terms of transparency, and make the code they used in their methods public (e.g. statswrapper.sh, AGAT, BUSCO, ETE Toolkit, Matplotlib, Seaborn). The code does not need to be executable, but a supplemental text and/or repository with as much of the starting data and commands executed as possible would make it easier for others to replicate this work and apply it to future comparative genomics projects.
Other than that, I found a couple minor issues:
3) Line 236: "…ploidy level, heterochromatin contente." This should be changed to "…ploidy level, and heterochromatin content."
4) Line 253-254: "…evolution of genome size is a long-standing question that is included in the so-called C-value Enigma [40]." The authors provide a citation, but I think this sentence would be stronger with a brief explanation of what the C-value Enigma is. Medusozoans are a great example of this "enigma", so it's worth reinforcing.
Congratulations to Santander et al. for a wonderful review!

Methods

Are the methods appropriate to the aims of the study, are they well described, and are necessary controls included? Choose an item.

Conclusions

Are the conclusions adequately supported by the data shown? Choose an item.

Reporting Standards

Does the manuscript adhere to the journal's guidelines on minimum standards of reporting? Choose an item.

Choose an item.

Statistics

Are you able to assess all statistics in the manuscript, including the appropriateness of statistical tests used? Choose an item.

Quality of Written English

Please indicate the quality of language in the manuscript: Choose an item.

Declaration of Competing Interests

Please complete a declaration of competing interests, considering the following questions:

- Have you in the past five years received reimbursements, fees, funding, or salary from an organisation that may in any way gain or lose financially from the publication of this manuscript, either now or in the future?
- Do you hold any stocks or shares in an organisation that may in any way gain or lose financially from the publication of this manuscript, either now or in the future?
- Do you hold or are you currently applying for any patents relating to the content of the manuscript?
- Have you received reimbursements, fees, funding, or salary from an organization that holds or has applied for patents relating to the content of the manuscript?
- Do you have any other financial competing interests?
- Do you have any non-financial competing interests in relation to this paper?

If you can answer no to all of the above, write 'I declare that I have no competing interests' below. If your reply is yes to any, please give details below.

I declare that I have no competing interests'

I agree to the open peer review policy of the journal. I understand that my name will be included on my report to the authors and, if the manuscript is accepted for publication, my named report including any attachments I upload will be posted on the website along with the authors' responses. I agree for my report to be made available under an Open Access Creative Commons CC-BY license (http://creativecommons.org/licenses/by/4.0/). I understand that any comments which I do not wish to be included in my named report can be included as confidential comments to the editors, which will not be published.

Choose an item.

To further support our reviewers, we have joined with Publons, where you can gain additional credit to further highlight your hard work (see: https://publons.com/journal/530/gigascience). On publication of this paper, your review will be automatically added to Publons, you can then choose whether or not to claim your Publons credit. I understand this statement.

Yes Choose an item.
