# Supplementary material for: The state of Medusozoa genomics: current evidence and future challenges
Source: Gigascience. 2022 May 17;11:giac036. doi: 10.1093/gigascience/giac036 (PMC9112765; doi:10.1093/gigascience/giac036)
Supplement: giac036_Reviewer_2_Report_Original_Submission — Lucas LeclÃ¨re -- 1/13/2022 Reviewed [file giac036_reviewer_2_report_original_submission.pdf]

Reviewer Report

Title: The state of Medusozoa genomics: current evidence and future challenges

Version: Original Submission **Date:** 1/13/2022

Reviewer name: Lucas LeclÃ¨re

Reviewer Comments to Author:

This manuscript offers a reanalysis of all available nuclear genomic data published on medusozoans. It represents a well though, and timely review of the available data, systematically comparing genomic features (repeated elements, intro/exon/gene size and numbers, chromosome numbers...) and genomic assemblies (available data, assembly quality and size…) in the different medusozoan classes. It largely confirms the results obtained from analysis of single species. It also provides useful guidelines for future standardization of genomic projects focused on medusozoans.
Minor comments and suggested corrections:
Line 118: How was "compiled all genomic and HTS metadata reference in this review", manually? If not, please provide the scripts used for this task.
Line 236: correct "contente"
Line 326: The sentence starting with "Moreover, even…" is unclear. Please clarify or delete.
Line 389: correct "proyects"
Figure 1: it would useful to indicate in this figure genome sizes calculated from genomic assemblies, in addition to genome sizes calculated from flow cytometry and feulgen densitometry estimations; either as a new column or using another color in C
SM_Table2: Suplementary Material S2 - Table S1 - please correct in the title "condidering".

Methods

Are the methods appropriate to the aims of the study, are they well described, and are necessary controls included? Choose an item.

Conclusions

Are the conclusions adequately supported by the data shown? Choose an item.

Reporting Standards

Does the manuscript adhere to the journal's guidelines on minimum standards of reporting? Choose an item.

Choose an item.

Statistics

Are you able to assess all statistics in the manuscript, including the appropriateness of statistical tests used? Choose an item.

Quality of Written English

Please indicate the quality of language in the manuscript: Choose an item.

Declaration of Competing Interests

Please complete a declaration of competing interests, considering the following questions:

- Have you in the past five years received reimbursements, fees, funding, or salary from an organisation that may in any way gain or lose financially from the publication of this manuscript, either now or in the future?
- Do you hold any stocks or shares in an organisation that may in any way gain or lose financially from the publication of this manuscript, either now or in the future?
- Do you hold or are you currently applying for any patents relating to the content of the manuscript?
- Have you received reimbursements, fees, funding, or salary from an organization that holds or has applied for patents relating to the content of the manuscript?
- Do you have any other financial competing interests?
- Do you have any non-financial competing interests in relation to this paper?

If you can answer no to all of the above, write 'I declare that I have no competing interests' below. If your reply is yes to any, please give details below.

I declare that I have no competing interests

I agree to the open peer review policy of the journal. I understand that my name will be included on my report to the authors and, if the manuscript is accepted for publication, my named report including any attachments I upload will be posted on the website along with the authors' responses. I agree for my report to be made available under an Open Access Creative Commons CC-BY license (http://creativecommons.org/licenses/by/4.0/). I understand that any comments which I do not wish to be included in my named report can be included as confidential comments to the editors, which will not be published.

Choose an item.

To further support our reviewers, we have joined with Publons, where you can gain additional credit to further highlight your hard work (see: https://publons.com/journal/530/gigascience). On publication of this paper, your review will be automatically added to Publons, you can then choose whether or not to claim your Publons credit. I understand this statement.

Yes Choose an item.
