# Supplementary material for: The state of Medusozoa genomics: current evidence and future challenges
Source: Gigascience. 2022 May 17;11:giac036. doi: 10.1093/gigascience/giac036 (PMC9112765; doi:10.1093/gigascience/giac036)
Supplement: giac036_Reviewer_3_Report_Original_Submission — Sheila Kitchen -- 1/17/2022 Reviewed [file giac036_reviewer_3_report_original_submission.pdf]

Reviewer Report

Title: The state of Medusozoa genomics: current evidence and future challenges

Version: Original Submission **Date:** 1/17/2022

Reviewer name: Sheila Kitchen

Reviewer Comments to Author:

Santander et al. review the state of genome assemblies and cytogenetics of Medusozoa. This review captures the progression of the sequencing efforts in the past decade and how the field is moving with new technological advances. From their assessment of the literature and unpublished data, they found that a weakness in their community is a general lack of standardization in analysis and limited availability of intermediate assembly components, such as the repeat libraries, and associated metadata. In the end they provide recommendations for standards to be applied to ongoing and future genomic projects.

I felt that these recommendations fell short of extending beyond basic requirements of publishing genomes today. While these recommendations are in line with recommendations of other genomic consortia (Vertebrate Genomes Project [Rhie et al. 2021, Nature], Sanger/Moore Aquatic Symbiosis Genomics, etc.) and most publishers including GigaScience (deposit data, reproducible methods, code availability statements, etc), they are quite general. I was left wondering if this was a commentary on the whole field of genomics. To that end, are there specific recommendations regarding medusozoans that would enhance data usage community wide that could be stated here? Are there established assembly pipelines (i.e. tools that provide the highest quality assemblies from various species) or types of sequencing effort (i.e. long read + HiC maps, transcriptome-informed gene annotation) that should be endorsed as part of your assessment? Are there specific taxonomic gaps that should be prioritized (starting Line 238)?

The majority of the resources you identified only have short-read Illumina data which inevitably means that chromosome-scale assemblies are not possible yet. However, these assemblies are sufficient for gene model comparisons across species (starting on Line 187). Is there a way to standardize gene prediction for cases where short reads may be all that is available? Re-analysis of gene predictions with different tools may lead to varying estimates and can lead to erroneous orthology assignments (see https://doi.org/10.1111/jpy.12947, https://doi.org/10.1371/journal.pbio.3000862, and https://www.biorxiv.org/content/10.1101/2022.01.13.476251v1). Re-analysis of Rhopilema gene content using different tools increases gene predictions closer to the median gene count you've found.

Regarding the recommendation for depositing intermediates into repositories (#3), is there one established for the community or are you referring to more general ones like Dryad, FigShare, Repbase, etc.? Providing an example genome project or two that shares these associated files might be helpful. There can be cost associated with hosting these resources. Do you see that as a barrier to researchers providing this sort of data?

A recommendation that is provided earlier in the paper is the call for lineage-specific single copy ortholog sets (Line 228). Should this be re-stated in the final recommendations as well?

Minor Comments:
Line 31-33: This sentence seems to be constructed of two thoughts but missing a connector between them.
Line 98: … assembly statistics using the statswrapper.sh script …
Line 169: … [55], and the …
Line 315: Remove "of" between reusing and previously.
Line 337: "reran" should be "rerun".
Line 389: Typo, "projects"
Figures: The resolution of the figures provided made it difficult to review. Specifically Figure 3 was quite pixelated.

Methods

Are the methods appropriate to the aims of the study, are they well described, and are necessary controls included? Choose an item.

Conclusions

Are the conclusions adequately supported by the data shown? Choose an item.

Reporting Standards

Does the manuscript adhere to the journal's guidelines on [minimum standards of reporting?](#) Choose an item.

Choose an item.

Statistics

Are you able to assess all statistics in the manuscript, including the appropriateness of statistical tests used? Choose an item.

Quality of Written English

Please indicate the quality of language in the manuscript: Choose an item.

Declaration of Competing Interests

Please complete a declaration of competing interests, considering the following questions:

- Have you in the past five years received reimbursements, fees, funding, or salary from an organisation that may in any way gain or lose financially from the publication of this manuscript, either now or in the future?

- Do you hold any stocks or shares in an organisation that may in any way gain or lose financially from the publication of this manuscript, either now or in the future?
- Do you hold or are you currently applying for any patents relating to the content of the manuscript?
- Have you received reimbursements, fees, funding, or salary from an organization that holds or has applied for patents relating to the content of the manuscript?
- Do you have any other financial competing interests?
- Do you have any non-financial competing interests in relation to this paper?

If you can answer no to all of the above, write 'I declare that I have no competing interests' below. If your reply is yes to any, please give details below.

I declare that I have no competing interests

I agree to the open peer review policy of the journal. I understand that my name will be included on my report to the authors and, if the manuscript is accepted for publication, my named report including any attachments I upload will be posted on the website along with the authors' responses. I agree for my report to be made available under an Open Access Creative Commons CC-BY license (http://creativecommons.org/licenses/by/4.0/). I understand that any comments which I do not wish to be included in my named report can be included as confidential comments to the editors, which will not be published.

Choose an item.

To further support our reviewers, we have joined with Publons, where you can gain additional credit to further highlight your hard work (see: https://publons.com/journal/530/gigascience). On publication of this paper, your review will be automatically added to Publons, you can then choose whether or not to claim your Publons credit. I understand this statement.

Yes Choose an item.
